# In-Plant Persistence and Systemic Transport of *Nicotiana benthamiana* Retrozyme RNA

**DOI:** 10.3390/ijms232213890

**Published:** 2022-11-11

**Authors:** Alexander A. Lezzhov, Eugene A. Tolstyko, Anastasia K. Atabekova, Denis A. Chergintsev, Sergey Y. Morozov, Andrey G. Solovyev

**Affiliations:** 1 A. N. Belozersky Institute of Physico-Chemical Biology, Moscow State University, 119992 Moscow, Russia; 2 Konstantinov St.-Petersburg Nuclear Physics Institute, National Research Center «Kurchatov Institute», 188300 Gatchina, Russia; 3 Department of Virology, Biological Faculty, Moscow State University, 119234 Moscow, Russia

**Keywords:** retrozyme, retroelements, long terminal repeat, circRNA, ncRNA, ribozyme, catalytic RNA, RNA systemic transport, RNA replication

## Abstract

Retrozymes are nonautonomous retrotransposons with hammerhead ribozymes in their long terminal repeats (LTRs). Retrozyme transcripts can be self-cleaved by the LTR ribozyme, circularized, and can undergo RNA-to-RNA replication. Here, we demonstrate that the *Nicotiana benthamiana* genome contains hundreds of retrozyme loci, of which nine represent full-length retrozymes. The LTR contains a promoter directing retrozyme transcription. Although retrozyme RNA is easily detected in plants, the LTR region is heavily methylated, pointing to its transcriptional silencing, which can be mediated by 24 nucleotide-long retrozyme-specific RNAs identified in *N. benthamiana*. A transcriptome analysis revealed that half of the retrozyme-specific RNAs in plant leaves have no exact matches to genomic retrozyme loci, containing up to 13% mismatches with the closest genomic sequences, and could arise as a result of many rounds of RNA-to-RNA replication leading to error accumulation. Using a cloned retrozyme copy, we show that retrozyme RNA is capable of replication and systemic transport in plants. The presented data suggest that retrozyme loci in the *N. benthamiana* genome are transcriptionally inactive, and that circular retrozyme RNA can persist in cells due to its RNA-to-RNA replication and be transported systemically, emphasizing functional and, possibly, evolutionary links of retrozymes to viroids—noncoding circular RNAs that infect plants.

## 1. Introduction

Circular RNAs (circRNAs) represent a distinct type of long noncoding RNA [1]. In most cases, circRNAs are products of “back-splicing”, a pre-mRNA splicing event in which a donor site is joined to an upstream acceptor site [1,2]. In mammalian cells, circRNAs, initially regarded as products of occasional splicing errors, appear to be involved in the regulation of a number of biological processes [3,4,5]. Two basic mechanisms of circRNA action are known. First, circRNAs can interact with proteins, thereby either influencing their decay/accumulation or modulating their functions, as in the case of circRNAs binding to RNA polymerase II and certain transcription factors resulting in the regulation of transcription [6]. Second, circRNAs can specifically interact with other RNAs. Some circRNAs have been shown to serve as “miRNA (micro-RNA) sponges” that bind to miRNAs and thus counteract the suppression of miRNA-targeted mRNAs, as shown, for example, for human CiRS-7 circRNA, which contains dozens of miR-7-binding sites and inhibits miR-7 functions [7,8]. Some other circRNAs interact with mRNAs, serving as “mRNA traps” that remove bound molecules from the pool of translatable mRNAs [9].

The investigations of plant-encoded circRNAs are at their very beginning. High-throughput sequencing of circRNA-enriched fractions of plant RNA and data analyses aimed at searching for back-spliced sites have resulted in the identification of tens to thousands of circRNAs in different plant species [10]. However, the currently available information on plant circRNA functions is very limited. Transcriptomic analyses reveal that circRNAs in all plant species analyzed can be differentially expressed under conditions of various stresses, including viral, bacterial, and fungal infections, as well as unfavorable temperatures, draught, and nutrient depletion [11], suggesting circRNA regulatory functions in response to different stimuli. Experimental evidence demonstrating their functional role is currently available for only a few plant circRNAs. In particular, circRNAs back-spliced from certain pre-mRNAs have been shown to downregulate the respective mRNAs. For example, the abundance of circRNAs derived from pre-mRNAs of tomato phytoene synthase 1 and phytoene desaturase is found to increase upon tomatoes ripening, and overexpression of any of these circRNAs results in decreased levels of the respective mRNA and the changed color of the tomato fruits [12]. In a similar manner, the delivery of an in vitro synthesized circRNA, which corresponds to a natural circRNA derived from exon regions of two RuBisCO small subunit genes, into *Arabidopsis thaliana* plants significantly suppresses the expression of these genes [13]. The mechanism of circRNA influence on the expression of a circRNA-encoding genomic locus has been demonstrated for a SEPALLATA3 gene-derived circRNA, which can bind to the gene forming an RNA–DNA hybrid that results in transcriptional pausing and alternative splicing [14]. Importantly, circRNAs can also regulate the expression of unrelated genes. For example, the overexpression of a grapevine circRNA termed circATS1 enhances cold tolerance and induces changes in the expression levels of more than two hundred protein- and miRNA-coding genes, including those involved in stress responses [15]. In addition, the downregulation of a tomato circRNA called Slcirc107 by gene silencing results in the considerable suppression of *Tomato yellow leaf curl virus* infection in tomato plants [16], suggesting that circRNAs may influence viral replication or transport in plants. It should be noted that the “miRNA sponge” function reported for animal systems has not been experimentally demonstrated for plant circRNAs, although some of those are predicted to contain potential miRNA-binding sites [11].

A specific class of plant genome-encoded circRNAs is represented by retrozyme RNA. Retrozymes (retrotransposons with hammerhead ribozymes) are retroelements found in the genomes of many plant and metazoan species [17,18,19]. Retrozymes, which encode no proteins, are nonautonomous transposons dependent on autonomous retroelements providing enzymatic transposition machinery and sharing with cognate retrozymes regulatory sequences essential for reverse transcription [17]. In general, the hammerhead ribozymes, as well as other, structurally distinct types of ribozymes including hepatitis delta virus-like ribozymes and twister ribozymes, are widespread in both prokaryotic and eukaryotic genomes and have been reported as conserved elements in different mobile genetic elements [18,20]. In particular, the hepatitis delta virus-type ribozymes are found in LINE retroelements, while the twister-type ribozymes are detected in Perere retrotransposons [21,22,23]. The hammerhead ribozymes, in addition to nonautonomous transposons such as retrozymes, are found in fully functional Penelope-like elements and Terminon giant retrotransposons [24,25]. Interestingly, the plant retrozymes are long terminal repeat (LTR)-containing genetic elements with the Type III hammerhead ribozyme, while the retrozymes in animals represent non-LTR transposons with the Type I hammerhead ribozyme [18], suggesting that retrozyme-containing genome elements in different kingdoms of life are evolutionarily distant.

Although retrozymes from different plant species have only moderate sequence similarity, in all cases they are delimited by four-base target-site duplications and display a similar sequence organization, including two flanking LTRs of approx. 300–400 bp with embedded ribozymes and a unique central region, which is flanked by the primer-binding site (PBS) complementary to the tRNA-Met 3′-terminal sequence and a polypurine tract (PPT) [17]. The RNA transcribed from retrozyme genomic loci undergoes self-cleavage by LTR ribozymes and circularization of the resulting monomeric retrozyme RNA. The PBS and PPT sequences are necessary for priming reverse transcription on the template of retrozyme RNA and the formation of double-stranded DNA during retrozyme mobilization for transposition, which may occur in the presence of a helper autonomous retrotransposon of the Ty3-gypsy family [17]. In addition to being a template for reverse transcription, retrozyme RNA has been suggested to undergo RNA-to-RNA replication, as concluded from the presence of both (+) and (−) strands of retrozyme RNA in plants [17,18].

In this paper, we report that the *N. benthamiana* genome contains hundreds of retrozyme loci, of which nine represent full-length retrozymes. We demonstrate that the retrozyme LTR region contains a promoter capable of directing retrozyme RNA synthesis and that the retrozyme LTR is heavily methylated in *N. benthamiana* leaves, suggesting that it is transcriptionally inactive. Accordingly, linear retrozyme-specific RNA is undetectable in plant leaves. We further show that the fraction of *N. benthamiana* small RNA contains 24-nucleotide-long retrozyme-derived RNAs that can be involved in the transcriptional silencing of the LTR promoter. Using transcriptome analysis, we demonstrate that 49% of retrozymes in the RNA population of *N. benthamiana* leaves have no exact matches in the genome and could arise as a result of RNA-to-RNA replication, leading to the accumulation of errors. Additionally, we provide evidence that retrozyme RNA is capable of replication and systemic transport in plants.

## 2. Results

### 2.1. Retrozymes in the N. benthamiana Genome

The genome of *N. benthamiana* has been reported to contain a single hammerhead ribozyme sequence and no retrozymes [17]. For a more in-depth analysis, we performed a BLAST search of *N. benthamiana* genome scaffolds (draft of the *N. benthamiana* genome, v.1.0.1; https://solgenomics.net, accessed on 16 September 2021) using, as a query, the 352-nucleotide-long LTR sequence of a previously characterized *Fragaria ananassa* retrozyme [17]. The search output contained 83 sequences with significant similarity to the query (e-value < 1 × 10^10^) that corresponded to full-length retrozyme LTRs or their fragments containing the LTR-embedded ribozyme sequence (Supplementary Figure S1) known to be conserved among retrozymes of different plant species [17]. A further BLAST search of the *N. benthamiana* genome, carried out with the newly identified *N. benthamiana* retrozyme LTR as a query, revealed 304 similar sequences (e-value < 1 × 10^10^) in 191 genomic scaffolds (Supplementary Table S1).

The individual analysis of *N. benthamiana* scaffolds containing two or more copies of LTR-related sequences revealed nine complete retrozymes named NbRZ1 to NbRZ9 (Supplementary Figure S2). According to comparative sequence analysis, the full-length *N. benthamiana* retrozymes fall into two groups: “Group 1” containing NbRZ1 to NbRZ7 and “Group 2” consisting of NbRZ8 and NbRZ9. Within each group, retrozyme sequences could be aligned along their entire length (Supplementary Figures S3 and S4). However, the similarity between the retrozymes of the two groups was detected mostly in a ribozyme-containing portion of LTR, whereas the retrozyme central region (CR) located between two LTRs exhibited no significant similarity between groups (Supplementary Figure S5). BLAST searches of the *N. benthamiana* genome with CR sequences as queries revealed 51 and 60 matches to Group 1- and Group 2-specific CRs, respectively (e-value < 1 × 10^10^), of which nine corresponded to previously identified complete retrozymes NbRZ1–NbRZ9, whereas others corresponded to incomplete retrozyme sequences. Collectively, these observations show that the *N. benthamiana* genome contains two groups of retrozymes, with each group being represented by full-length retrozymes, incomplete CR-containing retrozymes, and smaller retrozyme fragments containing mostly one LTR sequence.

Interestingly, NbRZ3 and NbRZ9 represented incomplete tandem repeats of the retrozyme sequence (Supplementary Figure S2), supporting the earlier suggestion that retrozymes may propagate in plants by RNA-to-RNA replication involving a rolling circle mechanism [17,18].

**Figure 1:**
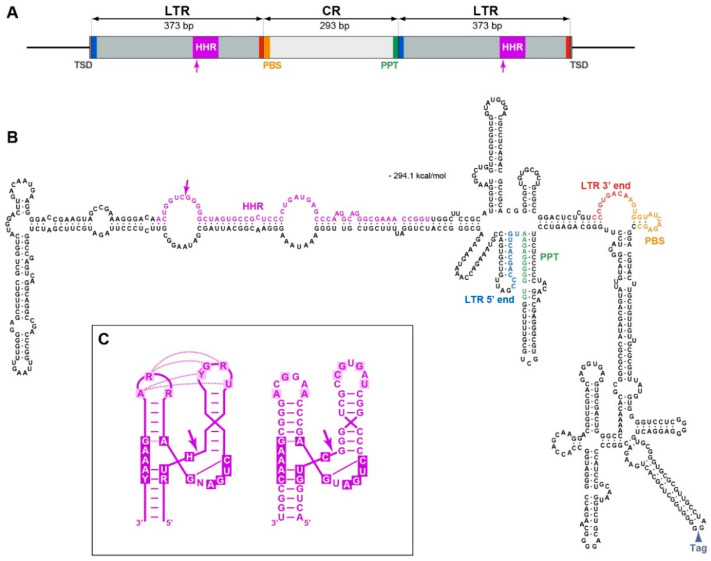
Structure of NbRZ1. (**A**) Schematic representation of the NbRZ1 genomic locus. The box indicates the NbRZ1 sequence, and flanking genomic regions are shown by lines. Lengths of LTRs and the CR are indicated. The position of the hammerhead ribozyme is shown in magenta, and the arrow points to the site of autocatalytic cleavage. PBS and PPT sequences are shown in yellow and green, respectively. (**B**) Predicted secondary structure of circular NbRZ1 RNA. Key structural elements are marked with the same colors as in (**A**). The arrowhead points to the site of tag insertion in the construct NbRZ1-Tag. Terminal LTR sequences are marked with blue and red in both (**A**) and (**B**) to facilitate their localization in the NbRZ1 secondary structure model. (**C**) Structural model of the NbRZ1 ribozyme. Left, base-pairing and key residues typical for Type III ribozymes. Residues forming the catalytic center are shown in white in magenta boxes, and dotted lines show noncanonical interactions in the ribozyme tertiary structure. Right, NbRZ1 ribozyme showing all essential features of Type III ribozymes.

The identified full-length *N. benthamiana* retrozymes were found to comprise characteristic sequence elements conserved in retrozymes of other species. As illustrated for NbRZ1 (Figure 1A), taken as a prototype Group 1 retrozyme, these characteristic genomic sequences include two copies of the LTR with a typical ribozyme sequence containing a self-cleavage site, a central region with a primer-binding site and a polypurine tract at its ends, and flanking four-base target site duplications typical of LTR retrotransposons. Similar to the retrozymes of other plant species, the *N. benthamiana* retrozyme RNAs of both groups potentially form extensive secondary structures (Figure 1B). The ribozyme sequences identical in both LTRs of NbRZ1, NbRZ2, NbRZ3, NbRZ6, and NbRZ7 were predicted to form folds perfectly matching the consensus structure of type-III hammerhead ribozymes (Supplementary Figure S6) and contained all nucleotide residues essential for the formation of specific tertiary structures and the enzymatic activity of such ribozymes (Figure 1C), suggesting their capacity for autocatalytic self-cleavage. In the other four retrozymes, substitutions that might affect the functions of ribozymes were found, and it remains unclear whether these ribozymes could be capable of self-cleavage (Supplementary Figure S6). Regardless of the functional competence of ribozymes, as all nine full-length *N. benthamiana* retrozymes were found to contain sequences required for reverse transcription, they might be mobilized for transposition in the presence of an active helper retrotransposon, presumably belonging, as suggested earlier, to the Ty3-gypsy family [17].

The two LTR sequences appeared to be identical only in NbRZ2 and NbRZ8, whereas other full-length *N. benthamiana* retrozymes had up to 32 mismatches between their two LTRs (Supplementary Figure S6). At the moment of genome integration, the two LTRs of a retrozyme should be identical due to the reverse transcription mechanism used by LTR-containing retrotransposons [18,26]; therefore, the mismatches between two LTRs of a single element could result from errors during the replication of the plant genome, and the number of accumulated mutations could reflect the evolutionary time since the retrozyme genome integration event. Thus, NbRZ4-NbRZ7 and NbRZ9 with 7 to 32 mismatches resulted from transposition events that occurred much earlier than those for NbRZ1–NbRZ3 and NbRZ8 (0 to 2 mismatches) (Supplementary Figure S6).

### 2.2. Retrozymes in the N. benthamiana Transcriptome

**Figure 2:**
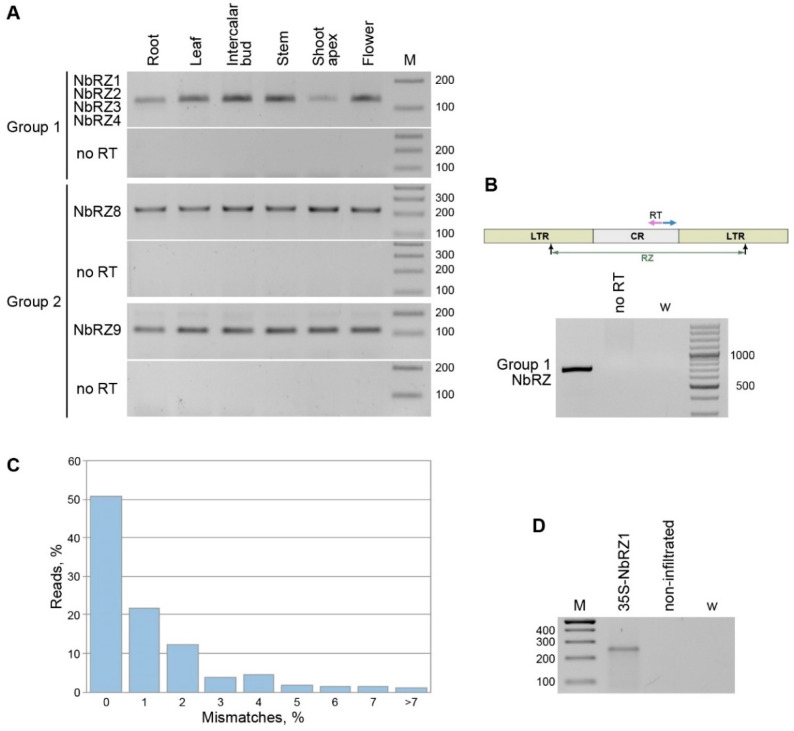
Analysis of retrozyme RNA in *N. benthamiana* plants. (**A**) Detection of retrozymes RNA in different plant parts, which are indicated above the gel. The specificity of the primers used and the retrozyme groups analyzed are indicated on the left. M—DNA size markers. (**B**) Percentage of retrozyme-specific reads with different numbers of mismatches with the closest genomic sequences. (**C**) Detection of full-length circular/concatemeric retrozyme RNA with a pair of divergent primers specific for Group 1 NbRZ. Positions of primers are shown by arrows on the retrozyme schematic representation above the gel. RT indicates the primer used for reverse transcription. (**D**) 5′-RACE analysis of NbRZ1-specific linear RNA in *N. benthamiana* leaves infiltrated with agrobacteria carrying the 35S-NbRZ1 construct and non-infiltrated leaves. W (water)—a control PCR without template added. M—DNA size markers.

To analyze the presence of retrozyme RNA in *N. benthamiana* plants, the total RNA preparations from the roots, stem, leaves, flowers, intercalary buds, and shoot apex were analyzed by reverse transcription-PCR with a pair of primers specific for CR sequences identical in the Group 1 retrozymes NbRZ1–NbRZ4 (Supplementary Table S4). To detect the Group 2 retrozymes, primers specific for NbRZ8 and NbRZ9 were used (Supplementary Table S4). The RNA of both retrozyme groups was easily detected in all the plant parts tested (Figure 2A), suggesting its ubiquitous presence in *N. benthamiana* plants. To verify the presence of full-length retrozyme RNA, reverse transcription-PCR with a pair of divergent primers specific for Group 1 retrozymes (Supplementary Table S4, Figure 2B) was carried out on the leaf RNA. The detection of a specific product (Figure 2B), the identity of which was confirmed by sequencing, demonstrated that *N. benthamiana* retrozymes can exist in the form of full-length circular/concatemeric RNA, like retrozymes in other plant species [17].

To further analyze retrozyme-specific RNA, a total preparation of leaf RNA was subjected to high-throughput sequencing (HTS). In the HTS raw data, reads that could be aligned to NbRZ1–NbRZ9 were identified. Initial analysis revealed that 14.8% of the retrozyme-specific reads aligned to the CR of the Group 1 retrozymes, 22.2% to the Group 2 CR, and 63.0% to LTR regions, confirming that both groups of *N. benthamiana* retrozymes were present in the plants in the RNA form.

Next, to identify transcriptionally active genomic retrozyme loci, the reads were assembled into contigs for comparison to identified genomic retrozymes. As the *N. benthamiana* genome contains many retrozymes that are close in terms of sequence, special precautions have been taken to avoid the assembly of reads corresponding to different RNAs into a single contig (see Section 4.7). As a result, 212 contigs were generated (Supplementary Table S2). An individual comparison of the contigs to the *N. benthamiana* genome revealed that only 88 (41.5%) contigs were identical to genomic sequences, whereas others had the closest genome matches with 88.4% to 99.7% identity. To verify that the observed contig differences from the genomic retrozyme copies did not result from an erroneous contig assembly, BLAST searches of the *N. benthamiana* genome were carried out using every retrozyme-specific read as a query. As a result, only 51.0% of the reads were found to have perfect matches in the *N. benthamiana* genome, whereas others had, relative to the closest genomic sequences, 1% mismatches (21.8% reads), 2% mismatches (12.4% reads), or 3 to 13% mismatches (14.8% reads) (Figure 2B). Thus, our transcriptome analyses demonstrate that only a portion of the retrozymes in the RNA population of *N. benthamiana* leaves corresponds to retrozyme genomic sequences, whereas others have no exact matches in the genome. We hypothesize that the latter subpopulation could arise as a result of the RNA-to-RNA replication of retrozymes in plants, leading to the accumulation of replication errors.

We further analyzed whether retrozyme genomic loci could be transcribed in *N. benthamiana*. Therefore, the rapid amplification of cDNA ends (5′-RACE) with Group 1 retrozyme-specific primers (Supplementary Table S4) was used to determine the presence of linear retrozyme transcripts in the total RNA preparations from the *N. benthamiana* leaves. As a positive control, 5′-RACE was carried out on the RNA isolated from *N. benthamiana* plants infiltrated with an agrobacterial culture carrying the 35S-NbRZ1 construct, which is a binary vector in which the full-length NbRZ1 sequence including two flanking LTRs was cloned under the control of the 35S promoter. A retrozyme-specific band was found in the control sample but not in the RNA from the non-infiltrated leaves (Figure 2C). By cloning and sequencing the amplification product, the 5′-end of the corresponding retrozyme linear RNA was mapped exactly to the guanosine residue predicted to be located at the 5′-terminus of the RNA product of retrozyme self-processing by the ribozyme located in the LTR (Figure 1B). Therefore, as the primary 35S promoter-driven NbRZ1 transcript was not detected by 5′-RACE, it should be entirely self-processed by the NbRZ1 ribozyme, confirming the functional competence of the predicted NbRZ1 ribozyme. Importantly, the absence of amplification products in the non-infiltrated leaves suggested that linear retrozyme-specific RNAs, including those derived from the transcription of genomic retrozyme loci and anticipated rolling-circle replication intermediates [17], could be present at very low levels, thereby preventing their detection. Importantly, the retrozyme transcripts self-cleaved by ribozymes, as well as the linear monomeric retrozyme RNA derived from the concatemeric RNA-to-RNA replication intermediates as a result of ribozyme processing, lack both the cap structure and the 3′-poly(A) and may be degraded by cellular exoribonucleases, unless these RNAs are circularized [27]. We hypothesize that the absence of retrozyme amplification products in the 5′-RACE analysis of samples from the non-infiltrated leaves can reflect the fast degradation of monomeric retrozyme RNA in plant cells, an effect that may be overwhelmed by the 35S promoter-driven high-level retrozyme expression in the infiltrated leaves. Thus, we assume that most retrozyme-specific RNAs detected by PCR and NGS are represented by circular retrozyme molecules persisting in the *N. benthamiana* leaves.

### 2.3. Retrozyme Transcriptional Silencing

**Figure 3:**
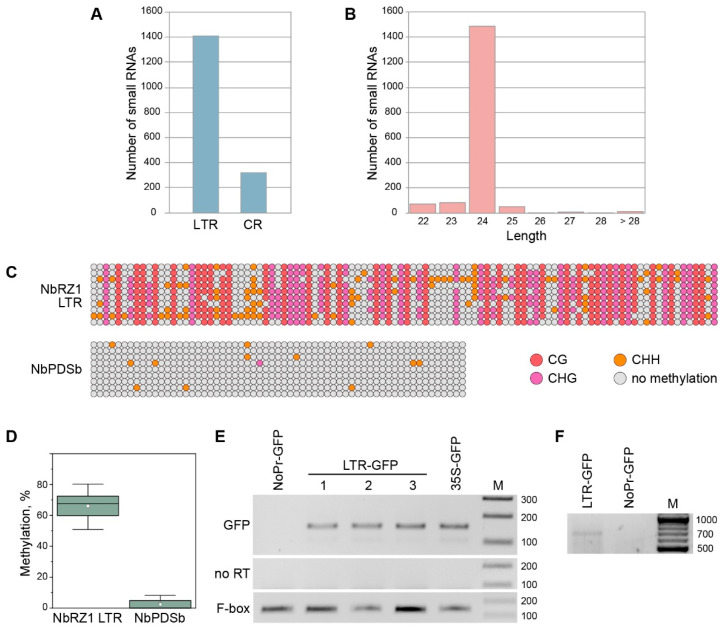
Silencing and transcription of retrozyme genomic loci. (**A**) Distribution of retrozyme-specific small RNAs between the CR and LTR regions. (**B**) Length distribution of retrozyme-specific small RNAs. Length in nucleotides is indicated. (**C**) Bisulfite sequencing analysis of cytosine methylation of the LTR region of the NbRZ1 genomic locus. A region of the NbPDSb promoter was analyzed in parallel as a control. Each row of circles represents one sequenced clone and shows cytosine residues in the analyzed regions as circles. Nonmethylated cytosine residues are shown in gray, and methylated cytosine residues are shown in color, as indicated. (**D**) Percentage of methylated cytosine residues in the NbRZ1 LTR region. The box represents the interquartile range (IQR), the horizontal line represents the median, white squares show the mean, and whiskers show the range within 1.5 IQR. (**E**) Analysis of the ability of the NbRZ1 LTR region to direct transcription. Total RNA from *N. benthamiana* leaves agroinfiltrated for the expression of constructs indicated above the gel was analyzed by RT–PCR. No RT—amplification carried out without reverse transcription. Primer specificity is indicated on the left. F-box primers were used as a control. (**F**) 5′-RACE analysis of RNA isolated from *N. benthamiana* leaves infiltrated with agrobacteria carrying either the LTR-GFP construct or the NoPr-GFP construct. M—DNA size markers.

Conceivably, highly structured retrozyme RNAs, similar to miRNA precursors, may undergo processing, giving rise to small RNAs acting in an miRNA-like manner to regulate the expression of particular genes, as shown for viroidstructured circular RNAs [28,29]. Therefore, we used publicly available libraries of HTS reads obtained for short RNA fractions isolated from different organs of *N. benthamiana* plants to determine whether retrozyme-derived small RNAs could exist in *N. benthamiana*. Searches with NbRZ1–NbRZ9 as queries revealed that small RNAs exactly matching retrozyme sequences could be found in all the plant organs (Supplementary Table S3). Only a minor number of retrozyme-specific small RNAs derived from CR, with most of them (81.6%) being fragments of the LTR region (Figure 3A). A length distribution analysis revealed that the vast majority of the retrozyme-specific small RNAs were 24 nucleotides in length (Figure 3B), suggesting that retrozyme RNA is processed by DCL3, which is known to generate small interfering RNAs (siRNAs) of this particular size [30,31].

The 24-nucleotide siRNAs direct the specific methylation of corresponding genomic sequences [31]. As the majority of retrozyme-specific small RNAs are derived from LTR, we carried out bisulfite sequencing to analyze the methylation status of retrozyme LTR in *N. benthamiana* genomic DNA using NbRZ1 as a model. After the bisulfite conversion of *N. benthamiana* DNA, the NbRZ1 LTR was amplified with a pair of flanking primers (Supplementary Table S4). As a control, a region of the NbPDSb (phytoene desaturase isoform b) promoter known to be methylated at a low level under normal plant-growing conditions [32] was amplified with the corresponding primers (Supplementary Table S4). The sequencing of the cloned amplification products revealed that the NbRZ1 LTR was heavily methylated (Figure 3C), whereas the NbPDSb control exhibited a minimal level of methylation, in agreement with a previous report [32]. In fact, 66.1% of the cytosine residues were methylated in the analyzed NbRZ1 LTR, showing a statistically significant difference from the control (Figure 3D).

The suppression of plant gene expression is often mediated by the methylation of promoter regions [33,34]. As the NbRZ1 LTR was found to be extensively methylated, we further analyzed whether the LTR region could contain a promoter able to direct the transcription of the NbRZ1 genomic locus. Therefore, we used a 35S-GFP construct in which the green fluorescent protein (GFP) gene was cloned under the control of the *Cauliflower mosaic virus* 35S promoter to replace the promoter region with the NbRZ1 LTR sequence. As a negative control, a 35S-GFP construct with a deleted 35S promoter region (NoPr-GFP) was generated. A reverse transcription-PCR analysis of the *N. benthamiana* leaves infiltrated with agrobacteria carrying the constructs LTR-GFP, 35-GFP, and NoPr-GFP revealed the presence of a GFP transcript directed by the NbRZ1 LTR (Figure 3E), suggesting that the LTR region had promoter activity. To characterize the LTR-directed transcript, 5′-RACE with GFP-specific primers was carried out on the RNA isolated from *N. benthamiana* leaves infiltrated with agrobacteria carrying the LTR-GFP construct. As expected, an amplification product was found for the LTR-GFP sample but not for the NoPr-GFP sample (Figure 3F). The cloning and sequencing of the LTR-GFP-derived product revealed that the 5′-terminus of the corresponding RNA mapped exactly to the 5′-guanosine residue of the RNA generated upon the ribozyme self-cleavage of the LTR RNA (Supplementary Figure S7). Thus, the transcription of LTR-GFP is initiated at a site upstream of the LTR-embedded ribozyme sequence, and then the primary transcript is self-cleaved to give rise to the RNA identified by the 5′-RACE analysis. As the self-cleaved RNA apparently lacked a 5′-cap structure, it could not be translated, that is in agreement with the absence of either GFP fluorescence in the agroinfiltrated leaves. Thus, due to the promoter activity of the LTR region and the location of the transcription start upstream of the ribozyme sequence, the full-length *N. benthamiana* retrozyme loci with two LTRs are self-sufficient for generating circularization-ready, unit-length retrozyme RNA. However, according to a small RNA analysis and the results of the LTR bisulfite sequencing, the LTR-driven transcription of retrozyme genomic loci can be considerably suppressed by the methylation of the LTR region mediated by the 24-nucleotide RNAs derived from retrozyme RNA.

### 2.4. Retrozyme Replication and Systemic Transport in N. benthamiana

**Figure 4:**
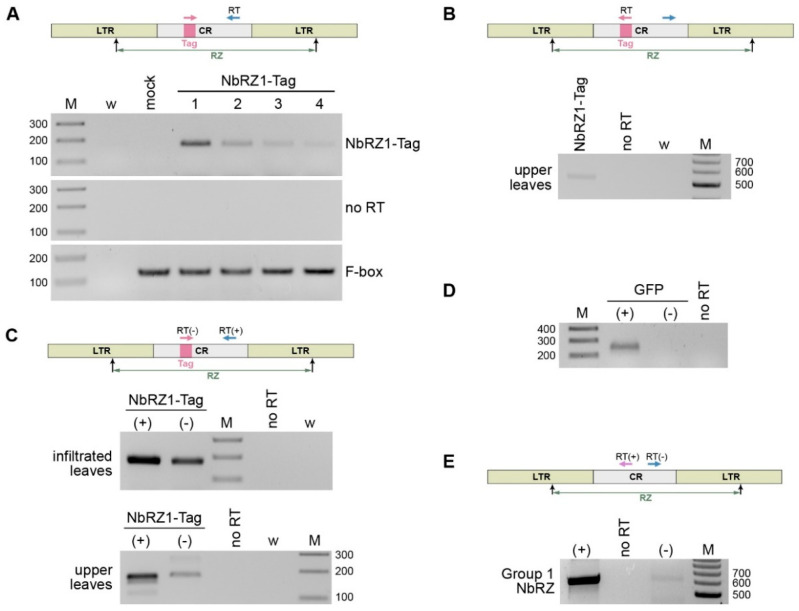
Systemic transport and replication of NbRZ1-Tag in *N. benthamiana* plants. (**A**) Reverse transcription-PCR analysis of the upper non-infiltrated leaves of plants, the lower leaves of which were agroinfiltrated for the expression of NbRZ1-Tag. Samples of four agroinfiltrated plants (numbered 1 to 4) and a non-infiltrated plant taken as a negative control (mock) were analyzed. Specificity of primers is indicated on the right. F-box primers were used as a control. No RT—amplification carried out without reverse transcription. W (water)—a control PCR without template added. M—DNA size markers. (**B**) Detection of circular/concatemeric NbRZ1-Tag RNA in the upper non-infiltrated leaves of plants, the lower leaves of which were agroinfiltrated for the expression of NbRZ1-Tag. Detection was carried out with a pair of divergent primers, one of which was specific for the tag. Location of the tag is shown on the retrozyme schematic representation above the gel; positions of primers are indicated by arrows. RT indicates the primer used for reverse transcription. (**C**) Detection of (+) and (−) RNA strands of NbRZ1-Tag in leaves infiltrated with agrobacteria carrying the NbRZ1-Tag construct and in upper non-infiltrated leaves. Reactions specific for (+) and (−) strands are indicated above the gel. The location of tag is shown on the retrozyme schematic representation above the gel; positions of primers are indicated by arrows. RT(+) and RT(−) indicate primers used for reverse transcription carried out for detection of the (+) and (−) strands of NbRZ1-Tag RNA, respectively. (**D**) Detection of (+) and (−) RNA strands of GFP transcript in leaves agroinfiltrated for GFP expression. One pair of GFP-specific primers was used for detection of both strands, with reverse and forward primers being used for reverse transcription carried out for detection of (+) and (−) GFP strands, respectively. (**E**) Detection of (+) and (−) RNA strands of circular/concatemeric Group 1 retrozymes in leaves of *N. benthamiana* plants. Detection was carried out with a pair of divergent primers. Positions of primers are shown by arrows on the retrozyme schematic representation above the gel. RT(+) and RT(−) indicate primers used for reverse transcription carried out for detection of the (+) and (−) strands of retrozyme RNA, respectively. No RT—PCR carried out on the same RNA sample without reverse transcription. W (water)—a control PCR without template added. M—DNA size markers.

Many RNAs with extensive secondary structures were found to be capable of long-distance transport via phloem [35]. As *N. benthamiana* retrozymes were predicted to be highly structured (Figure 1B), NbRZ1 was used as a model to study whether retrozymes could be spread throughout the plant body. For these experiments, to distinguish NbRZ1 transiently expressed by agroinfiltration from plant genome-encoded NbRZ1, the 35S-NbRZ1 construct (see above) was modified to carry a 24-nucleotide-long artificial tag sequence (35S-NbRZ1-Tag construct). As a tag insertion site, a loop of one of the predicted hairpins in the NbRZ1 CR was selected (Figure 1B), as this region was found to contain insertions in NbRZ5, NbRZ6, and NbRZ7 (Supplementary Figure S3). The lower leaves of the *N. benthamiana* plants were agroinfiltrated for the NbRZ1-Tag expression. Three days after infiltration, the upper non-infiltrated leaves of these plants were analyzed for the presence of the NbRZ1-Tag using reverse transcription-PCR with a pair of primers, of which one was complementary to the tag sequence (Supplementary Table S4). A product of the expected size was found in the plants agroinfiltrated for the expression of the NbRZ1-Tag but not in the control non-infiltrated plants (Figure 4A). Additionally, an analysis with a pair of divergent NbRZ1-Tag-specific primers (Supplementary Table S4, Figure 4B) revealed that the upper leaves contained circular/concatemeric NbRZ1-Tag RNA (Figure 4B), ruling out the possibility that only fragments of retrozyme RNA could be transported to the upper leaves. The cloning and sequencing of both amplification products (Figure 4A,B) confirmed their identity. Thus, the NbRZ1-Tag transiently expressed in the lower leaves of *N. benthamiana* plants is capable of systemic transport into the upper leaves.

As concatenated retrozyme copies were found in the *N. benthamiana* genome (NbRZ3 and NbRZ9, Supplementary Figure S2), *N. benthamiana* retrozyme RNA could likely undergo RNA-to-RNA replication through the rolling circle mechanism, as has been previously suggested for retrozymes from other plant species [17]. To analyze this possibility, the leaves agroinfiltrated for the expression of the NbRZ1-Tag were analyzed by reverse transcription-PCR for the presence of (+) and (−) strands of NbRZ1 RNA using either reverse or forward primer, respectively, for reverse transcription. One of the two primers used was specific for the tag sequence (Figure 4C). In this experiment, the (−) strand RNA of NbRZ1-Tag was easily detected (Figure 4C). Potentially, the appearance of (−) RNA in the agroinfiltrated leaves might result from the activity of RNA-dependent RNA polymerases, which are involved in RNA silencing and generate double-stranded RNAs in mRNAs expressed at high levels from introduced binary vectors [36]. To discard this possibility, the control leaves agroinfiltrated for the expression of GFP were analyzed. Using reverse transcription-PCR, the GFP mRNA was detected, whereas no product was found for the (−) strand of the GFP RNA (Figure 4D). Therefore, the detection of the NbRZ1-Tag (−) strand RNA supports the notion that retrozymes can undergo replication.

To determine whether the replication of native retrozyme RNA could occur, the presence of (+) and (−) strands of retrozyme RNA was analyzed in the *N. benthamiana* leaves with a pair of divergent primers specific for the Group 1 retrozymes (Supplementary Table S4, Figure 4E). In this experiment, retrozyme RNAs of both polarities were detected, with the (+) strand being more abundant (Figure 4E), suggesting the presence of circular/concatemeric forms of native retrozyme RNA in *N. benthamiana.* The sequencing of the cloned (−) strand-specific product confirmed its identity and revealed that all sequenced clones had no exact matches to retrozyme genomic loci (Supplementary Figure S8 that was in favor of the proposed error-prone retrozyme replication.

In line with this assumption, the reverse transcription-PCR analysis of the upper leaves of the plants agroinfiltrated for the expression of the NbRZ1-Tag carried out with a pair of primers, in which one was complementary to the tag sequence (Supplementary Table S4, Figure 4C), revealed both (+) and (−) strand-specific products (Figure 4C). The sequencing of cloned (−) strand-specific fragment demonstrated that all the analyzed clones contained mismatches to the NbRZ1-Tag sequence (Supplementary Figure S9A). Accordingly, the sequencing of the cloned (+) strand-specific PCR product obtained with divergent primers for the upper leaves of the plants agroinfiltrated for the expression of NbRZ1-Tag (see above, Figure 4B) revealed that two of three clones contained substitutions in the NbRZ1-Tag sequence (Supplementary Figure S9B). Collectively, the presented data support the hypothesis of the RNA-to-RNA replication of *N. benthamiana* retrozymes, and we suggest that retrozyme RNA transported to distant plant parts can replicate after unloading from the phloem.

## 3. Discussion

In this paper, we reveal that the *N.* benthamiana genome contains hundreds of retrozyme loci representing full-length retrozymes, incomplete retrozymes with truncated terminal regions, and retrozyme fragments containing mostly one LTR sequence. Interestingly, the retrozymes in the *N. benthamiana* genome fall into two distinct groups, in which central regions located between two LTRs are conserved within each group and dissimilar between groups. As both types of central regions have no sequence counterparts in other plants, the two retrozyme groups have likely evolved in the *N. benthamiana* genome.

The detection of retrozymes in the plant RNA preparations has led to the conclusion that genomic retrozyme loci are actively transcribed [17]. Indeed, we show here that retrozyme-specific RNAs can be easily detected in all the analyzed parts of *N. benthamiana* plants. However, we report data suggesting that *N. benthamiana* retrozyme-specific RNAs are largely products of RNA-to-RNA replication rather than transcripts of genomic retrozyme copies.

First, using both genome- and transcriptome-wide analyses, we demonstrated that half of the retrozyme-specific RNAs in the plant leaves do not match the genomic retrozyme copies. Similarly, in a limited set of 16 analyzed *Jatropha curcas* retrozyme cDNA clones, none were found to perfectly match known genomic retrozymes [17]. We assume that differences in the retrozyme-specific RNAs from genomic sequences are a result of retrozyme RNA-to-RNA replication leading to the accumulation of replication errors. As retrozyme RNAs can have a high percentage of substitutions compared to genomic sequences (up to 13% in retrozyme-specific sequence reads), presumably resulting from numerous rounds of RNA-to-RNA replication, it seems quite possible that retrozymes can persist and propagate in plant RNA populations for long periods of time.

Second, using the bisulfite sequencing of *N.* benthamiana genomic DNA, we showed that the LTR region of the prototype retrozyme NbRZ1 is heavily methylated. As the retrozyme LTR is shown to contain a promoter, LTR methylation likely suppresses the transcription of retrozyme genomic loci. This observation is in agreement with the view that the transcription of transposable retroelements in somatic cells is normally repressed by the methylation of respective genomic regions [37]. The transcriptional silencing of retrozyme DNA can be mediated by 24-nucleotide-long retrozyme-specific RNAs identified among the sequenced *N. benthamiana* small RNAs. Therefore, retrozyme transcription appears to be controlled by a negative feedback loop when the transcribed retrozyme RNA is processed into small RNAs directing the transcriptional silencing of retrozyme loci. The retrozyme RNA produced by RNA-to-RNA replication can be an additional source of retrozyme-specific small RNAs, which contribute to the further down-regulation of retrozyme genomic loci transcription.

We hypothesize that the population of retrozyme RNA may persist in plants and propagate through RNA-to-RNA replication, while the transcription of retrozyme DNA is suppressed. Conceivably, retrozyme transcription can be activated when the transcriptional suppression of transposable elements is released [38]. As a result, retrozyme genomic loci and a helper LTR retrotransposon can be simultaneously transcribed. In this case, the population of ribozyme RNA is replenished with transcripts of genomic retrozymes, and both newly transcribed and “old” molecules, which have undergone cycles of RNA-to-RNA replication, can be reverse-transcribed by a helper retrotransposon-encoded enzyme into DNA capable of integration into the plant genome. Indeed, as evident from the presence of concatenated retrozymes in the *N. benthamiana* genome, circular retrozyme RNAs and/or linear concatemer products of their rolling-circle replication can serve as templates for reverse transcription, giving rise to integration-competent DNA copies. The genomic integration of retrozymes carrying mutations accumulated during multiple cycles of RNA-to-RNA replication may (i) considerably expedite the evolutionary rate of these retroelements and account, at least in part, for the observed diversity of the genomic retrozyme copies and (ii) provide a mechanism for the fixation of mutations emerging in retrozyme RNA that might contribute to plant fitness if retrozyme RNA has an adaptive function.

Using a cloned copy of a full-length retrozyme, we have demonstrated that retrozyme RNA is capable of systemic transport from the lower leaves into the upper leaves of *N. benthamiana* plants. This finding is consistent with reports showing that many plant RNAs, including both mRNAs and noncoding RNAs, are transported through the phloem to distant plant parts [39] and that RNAs with pronounced secondary structures may contain signals of long-distance transport [35]. Retrozymes have been previously noted to be similar to viroids, noncoding circular RNAs that infect plants [40,41], in their extensive secondary structure, replication through the rolling-circle mechanism, and the presence, in the case of viroids of the *Avsunviroidae* family, of Type III hammerhead ribozymes [17,18]. Therefore, the discovery of retrozyme systemic transport shows that retrozymes and viroids have, in addition to structural similarities, a common functional feature that supports their suggested evolutionary link [17,18]. As proposed, the origin of viroids from retrozymes may be the most likely evolutionary scenario [20]. We hypothesize that retrozyme replication can be similar in its mechanism to the viroid RNA-to-RNA replication, which involves cellular DNA-dependent RNA polymerases, either RNA polymerase II in the case of nuclear viroids (family *Pospiviroidae*) or the RNA polymerase NEP in the case of viroids replicating in chloroplasts (family *Avsunviroidae*) [40,41].

While viroids, unlike retrozymes, have no genome-integrated DNA copies, a DNA counterpart has been found for carnation small viroid-like RNA (CarSV RNA), a ribozyme-containing circular RNA that is not transmitted from plant to plant and presumably replicates through a rolling-circle mechanism [42]. Multimeric CarSV DNA has been found to be integrated into the *Carnation etched ring virus* (CERV, a pararetrovirus) genome, which exists in infected cells as a circular episome [42], and, in some carnation cultivars, into microsatellite-like genome sequences [43]. Remarkably, CarSV RNA is nonidentical to CERV genome-integrated CarSV DNA copies [42], suggesting that the CarSV RNA detected in plants, similar to retrozymes, results from multiple rounds of RNA-to-RNA replication leading to the accumulation of errors, rather than represents a primary product of CarSV DNA transcription. Therefore, along with retrozymes, CarSV RNA can be considered another example of endogenous circular RNA that persists in plants.

Currently, the functions of retrozyme RNA in plants are unknown. Retrozyme RNAs might serve as molecular ‘sponges’ sequestering certain plant miRNAs and thus affecting miRNA-regulated pathways, as shown for circRNAs in animals [7,8]. However, this possibility seems unlikely, as NbRZ1–NbRZ9 have been found to contain no sequences with less than five mismatches to the *N. tabacum* miRNAs available in miRBase (http://www.mirbase.org, accessed on 16 September 2021) taken for analysis in the absence of annotated *N. benthamiana* miRNAs. Therefore, we hypothesize that the potential function(s) of retrozyme RNA in plants can be linked to its systemic transport abilities. If retrozyme RNA can serve as a signaling molecule, a target of such regulation remains to be identified.

## 4. Materials and Methods

### 4.1. RNA Detection in N. benthamiana Plants

Detection of NbRZ1–NbRZ4, NbRZ8, and NbRZ9 in different parts of *N. benthamiana* plants, as well as detection of GFP and NbRZ1-Tag RNA in agroinfiltrated plants, was carried out by reverse transcription-PCR with specific primers (Supplementary Table S4). As an input control, mRNA of F-box protein, which is ubiquitously expressed in *N. benthamiana* and used as a reference gene [44], was detected by reverse transcription-PCR with specific primers (Supplementary Table S4). Total RNA was extracted from plants using TRIzol reagent according to manufacturer’s instructions (ThermoFisher Scientific, Waltham, MA, USA) and treated with RNase-free DNAse I (ThermoFisher Scientific). For detection of retrozyme-specific RNA, to ensure reliable reverse transcription of highly structured templates, the reaction was carried out at 53 °C using RevertAid H Minus reverse transcriptase (ThermoFisher Scientific). Prior to reverse transcription, RNA was incubated at 85 °C for 5 min. Amplification was carried out with the high-fidelity Encyclo polymerase possessing proofreading activity (Evrogen, Moscow, Russia).

### 4.2. High-Throughput Sequencing

For high-throughput sequencing, RNA samples treated with RNase-free DNAse I (ThermoFisher Scientific) and devoid of contaminating genomic DNA were used. Depletion of ribosomal RNA was carried out with Ribozero Plant kit (Illumina, San Diego, CA, USA), while cDNA synthesis was carried out with Illumina TruSeq Stranded Total RNA kit using random primers. The quality of cDNA libraries was verified on Fragment Analyzer, while cDNA quantity was estimated by qPCR. cDNA libraries were sequenced on Illumina NovaSeq 6000 producing 100 nucleotide-long single reads. In total, 270 M reads were obtained.

### 4.3. 5′-RACE

The 5′-RACE analysis was carried out using Mint RACE kit (Evrogen) suitable for 5′-RACE on both capped and non-capped transcripts. This protocol employs the Step-Out RACE procedure described in detail earlier [45]. Reverse transcription and subsequent amplification steps were performed according to manufacturer’s instructions using primers included in the kit and gene-specific primers (Supplementary Table S4). Amplification products were cloned into the pAL2-T vector (Evrogen) and sequenced.

### 4.4. Bisulfite Sequencing

The *N. benthamiana* genomic DNA was isolated using a modified CTAB method [46]. Bisulfite conversion and cleanup of DNA was carried out with EpiTect Bisulfite Kit (QIAGEN, Hilden, Germany). The NbRZ1 LTR and the NbPDSb (phytoene desaturase isoform b) promoter region were amplified on the template of bisulfite-treated DNA with specific primers (Supplementary Table S4). Amplification products were cloned into the pAL2-T vector (Evrogen) and sequenced.

### 4.5. Molecular Cloning and Recombinant Constructs

To obtain the 35S-NbRZ1 construct, the NbRZ1 genomic locus was amplified on *N. benthamiana* genomic DNA with specific primers (Supplementary Table S4). The resulting amplification product was digested with appropriate restriction endonucleases and cloned into the pLH* binary vector [47]. The 35S-GFP construct was described previously as pLH-GFPC3 [48]. To obtain the LTR-GFP construct, the LTR region was amplified on the 35S-NbRZ1 template with LTR-specific primers (Supplementary Table S4) and cloned into the 35S-GFP construct to replace the 35S promoter. To obtain the NoPr-GFP construct, 35S-GFP was digested with EcoRI to excise the 35S promoter region, and the resulting plasmid was self-ligated. To obtain the 35S-NbRZ1-Tag construct containing an artificial tag sequence (GATTACAAGGATGACGATGACAAG), overlap-PCR with a pair of specific primers (Supplementary Table S4) was carried out. All constructs were verified by sequencing.

### 4.6. Agroinfiltration of Plants

Transformation of *Agrobacterium tumefaciens* (C58C1) cells with recombinant binary vectors was performed using the freeze–thaw method. For agroinfiltration, 5–6-week-old *Nicotiana benthamiana* plants kept in growth chambers (24/20 °C day/night temperatures, 16-h/8-h day/night periods, and 50% humidity) were used. In experiments with long-distance transport, plants with six leaves were used. The 2nd and 3rd leaves were agroinfiltrated for the NbRZ1-Tag expression; the 5th and 6th leaves were analyzed for the presence of NbRZ1-Tag at 3 days post infiltration. Agrobacteria for infiltration were prepared essentially as described (Solovyev et al., 2013). Agrobacterial cultures were grown overnight at 28 °C in LB medium containing 20 mM acetosyringone, 10 mM 2-(N-morpholino)ethanesulfonic acid (MES, pH 5.5), and appropriate antibiotics. Agrobacterial cells were centrifugated, resuspended in infiltration buffer (10 mM MES, pH 5.5, 10 mM MgCl_2_, and 150 mM acetosyringone), and incubated at room temperature for 3 to 4 h. Agrobacterial cell suspensions were diluted to the final optical density of 0.3 at 600 nm and infiltrated into abaxial surface of fully expanded leaves using a 2 mL needleless syringe.

### 4.7. Sequence Analysis

BLAST searches of *N.* benthamiana genome scaffolds were carried out at https://solgenomics.net (accessed on 16 September 2022). Multiple sequence alignments were generated with Clustal Omega (https://www.ebi.ac.uk/Tools/msa/clustalo/, accessed on 16 September 2022). RNA secondary structure predictions were made using RNAfold (http://rna.tbi.univie.ac.at//cgi-bin/RNAWebSuite/RNAfold.cgi, accessed on 16 September 2022).

The HTS libraries were analyzed for read quality using FastQC (https://www.bioinformatics.babraham.ac.uk/projects/fastqc/, accessed on 16 September 2022) and potential contamination using FastQ Screen [49]. To identify retrozyme-specific reads, read libraries were aligned to sequences of nine full-length *N. benthamiana* retrozymes (NbRZ1–NbRZ9) with Bowtie-2 [50], while the alignments were handled using SAMtools [51]. For comparison to genomic sequences, the reads were assembled into contigs. As many *N. benthamiana* retrozymes are close in terms of their sequences, we developed an algorithm for contig assembly that did not allow read mismatches and limited contig extension to 25 nucleotides at the addition of each following retrozyme-specific read identified as described above to prevent further anchoring of foreign reads to long single-read overhangs at contig ends. The generated contigs and the primary retrozyme-specific reads were aligned to *N. benthamiana* genome scaffolds using BLASTN [52]. 

For identification of retrozyme-specific small RNAs, publicly available small RNA HTS libraries (SRX1081183, SRX1081184, SRX1081185, SRX1081187, SRX1081188, SRX2502223, SRX2502225, and SRX2502227) were used. The libraries were downloaded using NCBI SRA Toolkit (http://ncbi.github.io/sra-tools/, accessed on 16 September 2022). The initial processing of the reads included quality control with FastQC and removal of adapter sequences with Trimmomatic [53]. The libraries were aligned to nine full-length *N. benthamiana* retrozymes (NbRZ1–NbRZ9) using Bowtie-2 [50]. Alignment processing was performed using SAMtools [51], alignment visualization was carried out with IGV [54].

## 5. Conclusions

Retrozymes have a dual nature, being both nonautonomous retrotransposons, which transiently go through the RNA phase of their life cycle, and replicating circRNAs. The findings reported in this paper, including the long-lasting persistence of retrozyme RNA in plants and its ability to systemically spread over the plant body, suggest that retrozyme RNA has potential undiscovered functions.

## Data Availability

High-throughput sequencing data can be found in GeneBank under BioProject accession number PRJNA767075.

## Supplementary Materials

The following supporting information can be downloaded at: https://www.mdpi.com/article/10.3390/ijms232213890/s1.
