# Supplementary material for: In-Plant Persistence and Systemic Transport of Nicotiana benthamiana Retrozyme RNA
Source: Int J Mol Sci. 2022 Nov 11;23(22):13890. doi: 10.3390/ijms232213890 (PMC9695139; doi:10.3390/ijms232213890)
Supplement: Supplementary file 1 [file ijms-23-13890-s001.zip › ijms-2030913-supplementary.pdf]

Supporting Information

In-Plant Persistence and Systemic Transport of *Nicotiana benthamiana* Retrozyme RNA

The following Supporting Information is available for this article:

Figure S1. Sequence similarity between LTR regions of *F. ananassa* and *N. benthamiana* retrozymes.

Figure S2. Sequences of full-length *N. benthamiana* retrozymes.

Figure S3. Sequence alignment of *N. benthamiana* Group 1 retrozymes.

Figure S4. Sequence alignment of *N. benthamiana* Group 2 retrozymes.

Figure S5. Conserved region in the LTRs of Group 1 and Group 2 retrozymes.

Figure S6. Analysis of potential ribozyme structures in *N. benthamiana* retrozymes.

Figure S7. 5'-RACE analysis of LTR-GFP transcription product.

Figure S8. Sequence analysis of Group 1 retrozyme (-) strand-specific amplification product obtained with divergent primers.

Figure S9. Sequence analysis of NbRZ1-Tag amplification products obtained for upper leaves of plants agroinfiltrated for expression of NbRZ1-Tag.

Table S1. Retrozyme-containing *N. benthamiana* genomic scaffolds.

Table S2. Contigs assembled from retrozyme-specific HTS reads.

Table S3. Retrozyme-specific small RNAs in *N. benthamiana* plants.

Table S4. Primers used in this study.

```
F.ananassa_Rtz-Fa-49c_LTR    TGTCACGTTCCGCTTTTGGGAGTGTCATCATGGGCGACACTGGTTGTCAAGCTGCATGGA
NbRZ1(Niben101Scf01887)      TGTCACGACCCGATTGTCG---TGACCGTAAAGACCAAATGAAAGAGCGGGCCATCTGGA
                             *******  *** ** * *   ** *   *  * * * *        *  **    ****

F.ananassa_Rtz-Fa-49c_LTR    TATGCGGGGTTGCGCTCCAAGATCAAGGAGTGTAGGGGCATACGTGCAGTCCCTTTGGAG
NbRZ1(Niben101Scf01887)      TTTTCGTCGTTGGGAAATAAAGGGAACGGCATTAGC-GATAAGGCGTTCTCCCTTAGATG
                             * * **  **** *    **    **      ***  * * * * *   ****** *  *

F.ananassa_Rtz-Fa-49c_LTR    T---------------TCAGGCAGGCCGACCCGGTTGAGTTTGGGGAGCTTGTCGCTGGT
NbRZ1(Niben101Scf01887)      CTTCGATCTGGCCGAGTCAGGCAGGCCGACCCGGTTAAGTTTGGGGAGCTTGTCGCTGGT
                                             ******************** ***********************

F.ananassa_Rtz-Fa-49c_LTR    CGAGGACTTAACGCTGAAGAGGGGACTAGGCCATCCCTACACAAACTGGTCGGGGTCTGG
NbRZ1(Niben101Scf01887)      CATGGACTTGACAATGAAGGGGACCGAAGTAGCCGAAAGGACAACTGGTCGGGGGCTAG
                             *  ****** **  ***** **     **              ************ ** *

F.ananassa_Rtz-Fa-49c_LTR    AGCCGATCCCCTGACGAGCCCAAGGCAGGGCGAAACCAGT-CGTTGCCTCGGTTGATAAG
NbRZ1(Niben101Scf01887)      TGCCGCTCCCCTGATGAGCCCAAGGCAGGGCGAAACCGGTTGGCTTCCGCATGTGGTAAG
                              **** ******** ********************** **  * * ** *   ** ****

F.ananassa_Rtz-Fa-49c_LTR    GCTGG-CTCCC-TGGCATGGGA--------GCGCCGCATGGGCCAAGTGTGGTGGCCGAA
NbRZ1(Niben101Scf01887)      GCTGGTCTGGGGTGGTATGGGACGCCTCAGACGCCGCACGGGCGCTGTGCGCTGCGC-CG
                             ***** **    *** ******         ******* ****   *** * **  *

F.ananassa_Rtz-Fa-49c_LTR    AGGGCTAGGCCCGTGACA
NbRZ1(Niben101Scf01887)      GACTCTCGTCCCGTGACA
                                 ** * *********
```

The ribozyme region (gray shading) spans F.ananassa ACTGGTCGGGGTCTGG / AGCCGATCCCCTGACGAGCCCAAGGCAGGGCGAAACCAGT and NbRZ1 ACTGGTCGGGGGCTAG / TGCCGCTCCCCTGATGAGCCCAAGGCAGGGCGAAACCGGT.

Figure S1. Sequence similarity between LTR regions of *F. ananassa* and *N. benthamiana* retrozymes. Sequence alignment of 352-nucleotide-long LTR of the *F. ananassa* retrozyme Rtz-Fa-49c (GeneBank accession number KX281162.1) with LTR of retrozyme identified in *N. benthamiana* genomic scaffold Niben101Scf01887 (https://solgenomics.net) referred to as NbRZ1. The ribozyme region is shown by gray shading. Asterisks indicate identical residues.

>NbRZ1(Niben101Scf01887)
TGTCACGACCCGATTGTCGTGACCGTAAAGACCAAATGAAAGAGCGGGCCATCTGGATTTTCGTCGTTGGGAAATAAAGGGAACGGCATTAGCGATAAGGCGT
TCTCCCTTAGATGCTTCGATCTGGCCGAGTCAGGCAGGCCGACCCGGTTAAGTTTGGGGAGCTTGTCGCTGGTCATGGACTTGACAATGAAGGGGACCGAAGT
AGCCGAAAGGGACAACTGGTCGGGGGCTAGTGCCGCTCCCCTGATGAGCCCAAGGCAGGGCGAAACCGGTTGGCTTCCGCATGTGGTAAGGCTGGTCTGGGGT
GGTATGGGACGCCTCAGACGCCGCACGGGCGCTGTGCGCTGCGCCGGACTCTCGTCCCGTGACAAGTGGTATCAGAGCCGGACGTACTTGCTGTTGTTGCGCG
TTTATGGTGGGGTCCTCGGGGGAGGACTGTGCGGGTGCGCGTTGCCTAGGGGGTGGCTCGCACTGAAGACGGCCCCATCCTGCAATGGTCTGCAGGGGCAAGA
CCGGGATGGCCACCAGCAAGGACGGTTGCACGAGGTGAGGTGCGACTGGGCCAAAACACGCGGCGCGCATAGCAGTATTGTACGGGATCCTTGGGCAGAGTCC
TTCTCCCCCTACAGCACGAGGGCGTGCTGCGTTTTCGGTGGGGGAGAATGTCACGACCCGATTGTCGTGACCGTAAAGACCAAATGAAAGAGCGGGCCATCTG
GATTTTCGTCGTTGGGAAATAAAGGGAACGGCATTAGCGATAAGGCGTTCTCCCTTAGATGCTTCGATCTGGCCGAGTCAGGCAGGCCGACCCGGTTAAGTTT
GGGGAGCTTGTCGCTGGTCATGGACTTGACAATGAAGGGGACCGAAGTAGCCGAAAGGGACAACTGGTCGGGGGCTAGTGCCGCTCCCCTGATGAGCCCAAGG
CAGGGCGAAACCGGTTGGCTTCCGCGTGTGGTAAGGCTGGTCTGGGGTGGTATGGGACGCCTCAGACGCCGCACGGGCGCTGTGCGCTGCGCCGGACTCTCGT
CCCGTGACA

>NbRZ2(Niben101Scf00825)
TGTCACGACCCGATTGTCGTGACCGTAAAGACCAAATGAAAGAGCGGGCCATCTGGATTTTCGTCGTTGGGAAATAAAGGGAACGGCATTAGCGATAAGGCGT
TCTCCCTTAGATGCTTCGATCTGGCCGAGTCAGGCAGGCCGACCCGGTTAAGTTTGGGGAGCTTGTCGCTGGTCATGGACTTGACAATGAAGGGGACCGAAGT
AGCCGAAAGGGACAACTGGTCGGGGGCTAGTGCCGCTCCCCTGATGAGCCCAAGGCAGGGCGAAACCGGTTGGCTTCCGCGTGTGGTAAGGCTGGTCTGGGGT
GGTATGGGACGCCTCAGACGCCGCACGGGCGCTGTGCGCTGCGCCGGACTCTCGTCCCGTGACAAGTGGTATCAGAGCCGGACGTACTTGCTGTTGTTGCGCG
TTTATGGTGGGGTCCTCGGGGGAGGACTGTGCGGGTGCGCGTTGCCTAGGGGGTGGCTCGCACTGAAGACGGCCCCATCCTGCAATGGTCTGCAGGGGCAAGA
CCGGGATGGCCACCAGCAAGGACGGTTGCACGAGGTGAGGTGCGACTGGGCCAAAACACGCGGCGCGCATAGCAGTATTGTACGGGATCCTTGGGCAGAGTCC
TTCTCCCCCTACAGCACGAGGGCGTGCTGCGTTTTCGGTGGGGGAGAATGTCACGACCCGATTGTCGTGACCGTAAAGACCAAATGAAAGAGCGGGCCATCTG
GATTTTCGTCGTTGGGAAATAAAGGGAACGGCATTAGCGATAAGGCGTTCTCCCTTAGATGCTTCGATCTGGCCGAGTCAGGCAGGCCGACCCGGTTAAGTTT
GGGGAGCTTGTCGCTGGTCATGGACTTGACAATGAAGGGGACCGAAGTAGCCGAAAGGGACAACTGGTCGGGGGCTAGTGCCGCTCCCCTGATGAGCCCAAGG
CAGGGCGAAACCGGTTGGCTTCCGCGTGTGGTAAGGCTGGTCTGGGGTGGTATGGGACGCCTCAGACGCCGCACGGGCGCTGTGCGCTGCGCCGGACTCTCGT
CCCGTGACA

>NbRZ3(Niben101Scf04922)
CCGTGACAAGTGGTATCAGAGCCGGACGTACTTGCTGTTGTTGCGCGTTTATGGTGGGGTCCTCGGGGGAGGACTGTGCGGGTGCGTTGCCTAGGGGGTGGCT
CGCACTGAAGACGGCCCCATCCTGCAATGGTCTGCAGGGGCAAGACCGGGATGGCCACCAGCAAGGACGGTTGCACGAGGTGAGGTGCGACTGGGCCAAAACA
CGCGGCGCGCATAGCAGTATTGTACGGGATCCTTGGGCAGAGTCCTTCTCCCCCTACAGCACGAGGGCGTGCTGCGTTTTCGGTGGGGGAGAATGTCACGACC
CGATTGTCGTGACCGTAAAGACCAAATGAAAGAGCGGGCCATCTGGATTTTCGTCGTTGGGAAATAAAGGGAACGGCATTAGCGATAAGGCGTTCTCCCTTAG
ATGCTTCGATCTGGCCGAGTCAGGCAGGCCGACCCGGTTAAGTTTGGGGAGCTTGTCGCTGGTCATGGACTTGACAATGAAGGGGACCGAAGTAGCCGAAAGG
GACAACTGGTCGGGGGCTAGTGCCGCTCCCCTGATGAGCCCAAGGCAGGGCGAAACCGGTTGGCTTCCGCGTGTGGTAAGGCTGGTCTGGGGTGGTATGGGAC
GCCTCAGACGCCGCACGGGCGCTGTGCGCTGCGCCGGACTCTCGTCCCGTGACAAGTGGTATCAGAGCCGGACGTACTTGCTGTTGTTGCGCGTTTATGGTGG
GGTCCTCGGGGGAGGACTGTGCGGGTGCGCGTTGCCTAGGGGGTGGCTCGCACTGAAGACGGCCCCATCCTGCAATGGTCTGCAGGGGCAAGACCGGGATGGC
CACCAGCAAGGACGGTTGCACGAGGTGAGGTGCGACTGGGCCAAAACACGCGGCGCGCATAGCAGTATTGTACGGGATCCTTGGGCAGAGTCCTTCTCCCCCT
ACAGCACGAGGGCGTGCTGCGTTTTCGGTGGGGGAGAATGTCACGACCCGATTGTCGTGACCGTAAAGACCAAATGAAAGAGCGGGCCATCTGGATTTTCGTC
GTTGGGAAATAAAGGGAACGGCATTAGCGATAAGGCGTTCTCCCTTAGATGCTTCGATCTGGCCGAGTCAGGCAGGCCGACCCGGTTAAGTTTGGGGAGCTTG
TCGCTGGTCATGGACTTGACAATGAAGGGGACCGAAGTAGCCGAAAGGGACAACTGGTCGGGGGCTAGTGCCGCTCCCCTGATGAGCCCAAGGCAGGGCGAAA
CCGGTTGGCTTCCGCATGTGGTAAGGCTGGTCTGGGGTGGTATGGGACGCCTCAGACGCCGCACGGGCGCTGTGCGCTGCGCCGGACTCTCGGCCCGTGACA

>NbRZ4(Niben101Scf19517)
TGTCACGACCCGATTGTCGTGACCGTAAAGACCAAATGAAAAAGCGTGCCATCTGGATTTTCGTCGTTGGGAAATAAAGGGAACGACATTAGCGATAAGGCGT
TCTCCCTTAGATGCTTCGATCTGGCCGAGTCAGGCAGGCCGACCCGGTTAAGTTTGGGGAGCTTGTCGCTGGTCATGGACTTGACAATGAAGGGGACCGAAGT
AGCCGAAAGGGACAACTGGTCGGGGGCTAGTGCCGCTCCCCTGATGAGCCCAAGGCAGGGCGAAACCGGTTGGCTTCCGCGTGTGGTAAGGCTGGTCTGGGGT
GGTATGGGACGCCTCAGACGCCGCACGGGCGCTGTGCGCTGCGCCGGACTCTCGTCCCGTGACAAGTGGTATCAGAGCCGGACGTACTTGCTGTTGTTGCGCG
TTTATGGTGGGGTCCTCGGGGGAGGACTGTGCGGGTGCGCGTTGCCTAGGGGGTGGCTCGCACTGAAGACGGCCCCATCCTGCAATGGTCTGCAGGGGCAAGA
CCGGGATGGCCACCAGCAAGGACGGTTGCACGAGGTGAGGTGCGACTGGGCCAAAACACGCGGCGCGCATAGCAGTATTGTACGGGATCCTTGGGCAGAGTCC
TTCTCCCCCTACAGCACGAGGGCGTGCTGCGTTTTCGGTGGGGGAGAATGTCACGACCCGATTGTCGTGACCGTAAAGACCAAATGAAAGAGCGGGCCATCTG
GATTTTCGTCGTTGGGAAATAAAGGGAACGGCATTAGCGATAAGGCGTTCTCCCTTAGATGCTTCGATCTGGCCGAGTCAGGCAGGCCGACCCGGTTAAGTTT
GGGGAGCTTGTCGCTGGTCATGGACTTGACAATGAAGGGGACCGAAGTAGCCGAAAGGGACAACTGGTCGGGGGCTAGTGCCGCTCCCCTAATGAGCCCAAGG
CAGGGCGAAACCGGTTGGCTTCCGCATGTGGTAAGGCTGGTCTGGGGTGGTATGGGACGCCTCAGACGCCGTACGGGCGCTGTGCGCTGCGCCGGACTCTCGG
CCCGTGACA

>NbRZ5(Niben101Scf00321)
TGTCACGACCCAATTGTCGTGACCGTTAAGATCAAAGAAAGAGCGGACCATCTGGATTTTCGTCGTTGGGAAATAAAGGGAATGGCATTAGCAATAAGGGCGT
TCTCCCTAAGATGCTTTGGTCTTGCCGAGTTAGGCAGGCCGACCCGGTTAAGTTTGGGGTGCTTGTCGCTGGTCATGGACTTGACTATGAAGCAGACCGAAGC
AATCCTAAGGGACAACTGGTCGGGAGCTAGTGCCGCTCCCCTGATGAGCCCAAGGCAGGGCGAAACCGGTTGGCTTCCGCATGTGGTAAGGCTGGTCTCGGGG
TGGTATGGGACGCCTCGGGACGCTGCACGGGCGCTGTGTGCTGCGCCGGACTCTCGGCCCGTGACAAGTGGTATCAGAGCAGGACGTACTTGCTGTTGTTGCG
CGTTATGGTGGGATCCTCAGGGGAGGATTGTGCGGGTGCGTTGGCCTAAGTTTAATCCCGGACGGTGAGGGCATCGTGGCACGCACTGAAGACTGCCCCATCC
TGCAAGGTCAACCGGGTGACCGGTGGATAGCTACCAGCAAGGCCGGTTGCACAAGGCGTGATGCGACTGAGCCAAAGGGATGCGGCGCGCAAAGCAGTATTGT
ACGGGATCCTTGGGCAGAGTCCTTCTCCCCAACAGCACGAGGGCGTGCTGGATTTTGGGTGGGGGAGAATGTCACGACCCGATTGTCGTGACTGTTAAGATCA
AATGAAAGAGCGGACCATCTGGATTTTCGTTGTTGGGAAATAAAGGGAACGGCATTAGCAATAAGGGCGTTCTCCCTAAGATGCTTTGGTCTTGCCGAGTTAG
GCAGGCCGACCCGGTTAAGTTTGGGGCGCTTGTCGCTGGTCATGGACTTGACTATGAAGCAGACCGAAGCAATCCTAAGGGACAACTGGTCGGGAGCTAGTGC
CGCTCCCCTGATGAGCCCAAGGCAGGGCGAAACCGGTTGGCTTCCGCATGTGGTAAGGCTGGTCTCAGGGTGGTATGGGACGCCTCGGGACACCGCACGGGCG
CTGTGTGCTGCGCCGGACTCTCGGCCCGTGACA

>NbRZ6(Niben101Scf06068)
TGTCACGACCCGTTTGTCGTGACCGTGTAGTCCAAAGGAAAGAGCGGGCCATCTGGAACTTCGCCATTGGGCTACGGGGACGACATTAGCAATAAGGCGTTGT
TCTCCCTTAGATGCTTCGATCTGGCCGAGTCAGGCAGGTCGACCCGGTTAAGCTTGGGGTGCTTGTCGCTGGTCATGGACTTGACAACGAAGGGGACCAAAGT
AGGCCTAAGGGATAAACTGGTCGGGGGCTAGTTCCGCTCCCCTGATGAGCCCAAGTCAGGGCAAAACCGGTTGGCTTCCGCGTGTGGTAAGGCTTGTCTCGAG

```
GTAGTGGGCGGCCTCAGGACGCCGCACAGGCGCTGTGTGCTGCGCCGGACTCTTAGCCCGTGACAATTGGTATCAGAGCGGGACGTACTTGCTGCTGCGCGTT
ATGGTGGGGTCCTCAAGGGAGGACTGTGCGGGTGCGTTGCCTAGGCGGGGGGGGGGGCTCGTGCCGACGACTGCCCCATCCTGCTATGGTCTGCAGGGGAAAG
ACCGGTATGGCCACCAGCAAGGCCGGTTGCACGAGGTGAGGTGCGATTGAGCAAAGAGACGCGGCGCGTTAAGCAGTATTGTGCGGGTTCCTGGGTGGAGTCC
TTCTTCCCAACAGCACGAGGACGTGCTGGGTTTTGGGTGGGGGAGAATGTCACGACCCGTTTGTCGTGATCGTGTAGTCCAAAGGAAAGAGCGGGCCATCTGG
AACTTTTTCGTTGGGTTACGGGGACGACATTAGCAATAAGACGTTCTCCCTTAGATGCTTCGATCTGGCCGAGTCAGGCGGGCTGACCCGGTTAAGTTTAGGG
TGCTTGTCGCTGGTCATGGACTTGACAATGAAGGGGACCGAAGTAAGCCTAAAGGATCAACTGGTCGGGGGCTAGTGCCGCTCCCCTAATGAGCCCAAGGCAA
GGCGAAACCGGTTGGCTTCCGCGTATGGTAAGGCTTGTCTCGAGGTAGTGGGCTGCCTCGAGACGCTGCACGGGCGCTGTGTGCTGCGCCGAACTCTCAGCCC
GTGACA
```

```
>NbRZ7(Niben101Scf00379)
TGTCACGACCCGATTGTCGTGACCGATGAGATCACATGAAAGAGCGGACCATCTGGCAATTTGTCGTTGGGAATAAGGGAACGGCATTAGCAATAAGGACGTT
CTCCCTAAGATGCTTTGGTCTTGCTGAGTCAGGCAGGCCGACCCGGTTAAGTTTGGGGCGCTTGTCGCTTGTCTTGGAAATGACTATGAAGTTGACCGAAGCA
ATCCTAAGGGACAACTGGTCGGGGGCTAGTGCCGCTCCCCTGACGAGCCCAAGGCAGGGCGAAACCAGTTGGCATCCGCGTACACGATTAGGCTTGTCTCGGG
GTATGGCGGGCCTTGGGACGCCGCACGGGCGCCGCACGCTGTGCCGGACTCTCAGCCCGTGACAAGTGGTATCAGAGCAGGACGTACATTTGCTGGGTCTGCG
CGCTATGGTGGGGGTCCTCACAGAAAGGACTGAGCGGGTGCGTTTGCCTACACACACATTCCGGATGGTGAGCGCAGCACCGTGGCACGCACTGACGACTGCC
CTATCCTGCAAGGTCTTTCCGGACGACCGGTGGATGGCCACCAAGCCATGCCCGTTGCCATCAAGTGACTGGGCACAATCACGCGGCGCGTATAGCAGCATTG
TACGGGATCCTTGGGCAGAGTCCTTCTCCCCATCAGCACGAGGACGTGCTGCGACATGGGTGGGGGAGAATGTCACGACCCGATTGTCGTGAACGATGAGATC
ACATGAAAGAGCAGACCATCTGGCAATTCGTCGTTGGGAATAAGGGAACGGCATTAGCAATAAGGGCGTTCTCCCTAAGATGCTTTGGTCTTGCCGAGTCAGG
CAGGCCGACCCGGTTAAGTTTGGGGCGCTTGTCGCTTGTCTTGGACATGACTATGAAGTTGACCGAAGCAATCCTAAGGGACAACTGGTCGGGGGCTAGTGCC
GCTCCCCTGACGAGCCCAAGGCAGGGCGAAACCAGTTGGCATCCGCGTACACGATTAGGCTTGTCTCGTGGTATGGCGGGGCCTCGGGACGCCGCACGGGCGC
CACACGCTGCGCCGGACTCTCAGCCCGTGACA
```

```
>NbRZ8(Niben101Scf00177)
TGTCACGTCCTCAGAGAAGGGGCAAAGTAAAGCGTGCGGATTGGCATCAAACTGCCTTGGAGAGGGGAGGCGTCTCTAGCCATTAAGGCGCTCTGCCCTAGTT
GCGTATTGGCTTGGCCGAGTTAGGCATGCCGACCCGATTAAGTTTGGGGAGCATGTCGCTTGTCTTGGACATGACTATGAAGGGAGCCGAAGCATACGAGGGG
CACAGCTGGTCGGGAGCTATTGCCGCTTCCCTGATGAGCCCATGGCAGGGCGAAACCAGTCTGCGCGCACAGGCAGGACGGGCGTGTCCGGGGCGGTATGGGA
CGCCTCGGGCGCGGAGCGCCGCGGTCCGTGTCATTTGGTATCAGAGCAAACATGCTCTTCAACTTCTGTGGCGAGGTGTCAAGCTCATGAATGCACAAAGCTA
GCGGAAGGCATAAGTGCAAAGATGGGGGCGCTGAGTTACAGAGTGGGGCATGACGGTCCTTGGGGCAAACATACTACTTCTCCGCCAAAGTAAAGCGGGCTTG
AGCAAATGAAATTGTTGCGAACTCTAGATGAAACCACGAAGTGGACGCGCCGACGAAGTGTGAAGCTCTCCATGGTTTCGTGCCTAAGTCCGAGGTAGGCAGC
ACGAGGAGGTATTGCTAGCATGATGGGTGCAATGTTCCGAGTGGAAGACGAAAGGAGTAGCAGCGAAAGTAATGCGTCGGGTCGCCGCATCAGCCAGGTGGGG
TAGGGTTGTCACGTCCTCAGAGAAGGGGCAAAGTAAAGCGTGCGGATTGGCATCAAACTGCCTTGGAGAGGGGAGGCGTCTCTAGCCATTAAGGCGCTCTGCC
CTAGTTGCGTATTGGCTTGGCCGAGTTAGGCATGCCGACCCGATTAAGTTTGGGGAGCATGTCGCTTGTCTTGGACATGACTATGAAGGGAGCCGAAGCATAC
GAGGGGCACAGCTGGTCGGGAGCTATTGCCGCTTCCCTGATGAGCCCATGGCAGGGCGAAACCAGTCTGCGCGCACAGGCAGGACGGGCGTGTCCGGGGCGGT
ATGGGACGCCTCGGGCGCGGAGCGCCGCGGTCCGTGTCA
```

```
>NbRZ9(Niben101Scf03939)
GTCCGTGACAGAGTGGTATTAGAGCAAACATGCTCTTCAACTTCTCTTGCGAGGTGTCAGCTCATGAATGTAAGAGCTCGAGTGAATGCTACAAGTGCAAAGA
TGGGGGCGCTGAGTGACAGAGTGGAGCATGACGGTCCTTGGGCAAACATACTACTTCTCTGCCAAAGTAAAGCGGGCTTGAGCAATGAAATTGTTGCGAACTC
TAGATGAAACCACGAAGTGGACGCACCGACGAAGTGTGATGCCGTCTCCATGATTACGTGCCTGAGTCCCAAAGTTGGCAGCACGAGGAGGTGTGGTGCAAGC
ATGAATGAGGGTGCAAGTTCCGTGTGGAAGACGAAAGGAGTTGCAGCGAAAGTAATGCGTCGGGTCGCCGCATCATCGGGTGGGGGAGAGTTGTCACGGCCTC
AGAGTAGGGACAAAGAAAAGCGTGCGGATTGGCATCAAACTGCCTTGGGGAGGGGAGGTGCCTCTAGCCATAAAGGCGCTCTGCCCTAGTTGCGTATTGGCTT
GGCCGAGTTAGGCATGCCGACCCGATTAAGTTTGGGGAGCATGTCGCTTGTCTTGGACATGACTATGAAGGGAGCCGAAGCATACGGGGGGCACAGCTGGTCG
GGAGCTATTGCCGCTTCCCTGATGAGCCCATGGCAGGGCGAAACCAGTCTGCGCGCTAGGTCGGACGGGCGTGTCCGGGGCGGTTATGGGACGCCTCGGGCGC
GAAGCGCCGCGGTCCGTCACATTTGGTATCAGAGCAAACATGCTCTTCAACTTCTCTTGCAAGGTGTCAGCTCATGAATGCACAAGCTCGGGTGAATGCTACA
AGTGCAAAGATGGGGGCGCTGAGTGACAGAGTGGAGCATGACGGTCCTTGGGCAAACATACTACTTCTCCGCCAAAGTAAAGCGGGCTTGAGCAATGAAATTG
TTGCGAACTCTAGATGAAACCACGAAGTGGACGCACCGACGAAGTGTGATGCCGTCTCCATGATTACGTGCCTGAGTCCCAAAGTTGGCAGCACGAGGAGGTG
TGGTGCAAGCATGAATGAGGGTGCAAGTTCCGTGTGGAAGACGAAAGGAGTTGCAGCAAAATTAATGCGTCGGGTCGCCGCATCATCGGGTGGGGGAGAGTTG
TCAAGGCCTCAGAGCAGGGACAAAGTAAAGCGTGCGGATTGGCATCAAACTGTCTTGGGAAGGAGAGGTGCCTCTAGCCATAAAGGCGCTCTGCCCTAGTTGC
GTATTGGCTTGGCCGAATTAGGCATGCCGACCCGATTTAGTTTGGGGAGCATATCGCTTGACTTGGACATGACTATGAAGGGAGCCGAAGCATACGGGGGGCA
GAGCTGGTCGGGAGCTATTGCCGCTTCCCTGATGAGCCCATGGCAGGGGCGAAACCAGTCTGCGCGCTAGGTCGGACGAGCGTGTCCGCGGCGGTTATGAGAC
GCCTCGGGCGCGAAGCGCCGCGGTCCGTCACA
```

Figure S2. Sequences of full-length *N. benthamiana* retrozymes. Scaffolds containing the retrozymes are indicated in parentheses (https://solgenomics.net). LTR regions are marked with yellow color.

```
NbRZ1    TGTCACGACCCGATTGTCGTGACCGTAAAGACCAAATGAAAGAGCGGGCCATCTGGATTTTCGTCGTTGGGAAATAAAGGGAACGGCATT
NbRZ2    TGTCACGACCCGATTGTCGTGACCGTAAAGACCAAATGAAAGAGCGGGCCATCTGGATTTTCGTCGTTGGGAAATAAAGGGAACGGCATT
NbRZ3    TGTCACGACCCGATTGTCGTGACCGTAAAGACCAAATGAAAGAGCGGGCCATCTGGATTTTCGTCGTTGGGAAATAAAGGGAACGGCATT
NbRZ4    TGTCACGACCCGATTGTCGTGACCGTAAAGACCAAATGAAAAAGCGTGCCATCTGGATTTTCGTCGTTGGGAAATAAAGGGAACGACATT
NbRZ6    TGTCACGACCCGTTTGTCGTGACCGTGTAGTCCAAAGGAAAGAGCGGGCCATCTGGAACTTCGCCATTGGG---CTACGGGGACGACATT
NbRZ5    TGTCACGACCCAATTGTCGTGACCGTTAAGATCAA-AGAAAGAGCGGACCATCTGGATTTTCGTCGTTGGGAAATAAAGGGAATGGCATT
NbRZ7    TGTCACGACCCGATTGTCGTGACCGATGAGATCACATGAAAGAGCGGACCATCTGGCAATTTGTCGTTGGG-AATAA-GGGAACGGCATT
         ***********  ************   **  **   **** ****  ********   ** * * *****     * ***** * ****

NbRZ1    AGCGAT-AAGGCGT---TCTCCCTTAGATGCTTCGATCTGGCCGAGTCAGGCAGGCCGACCCGGTTAAGTTTGGGGAGCTTGTCGCTGGT
NbRZ2    AGCGAT-AAGGCGT---TCTCCCTTAGATGCTTCGATCTGGCCGAGTCAGGCAGGCCGACCCGGTTAAGTTTGGGGAGCTTGTCGCTGGT
NbRZ3    AGCGAT-AAGGCGT---TCTCCCTTAGATGCTTCGATCTGGCCGAGTCAGGCAGGCCGACCCGGTTAAGTTTGGGGAGCTTGTCGCTGGT
NbRZ4    AGCGAT-AAGGCGT---TCTCCCTTAGATGCTTCGATCTGGCCGAGTCAGGCAGGCCGACCCGGTTAAGTTTGGGGAGCTTGTCGCTGGT
NbRZ6    AGCAATAA-GGCGTTGTTCTCCCTTAGATGCTTCGATCTGGCCGAGTCAGGCAGGTCGACCCGGTTAAGCTTGGGGTGCTTGTCGCTGGT
NbRZ5    AGCAATAAGGGCGT---TCTCCCTAAGATGCTTTGGTCTTGCCGAGTTAGGCAGGCCGACCCGGTTAAGTTTGGGGTGCTTGTCGCTGGT
NbRZ7    AGCAATAAGGACGT---TCTCCCTAAGATGCTTTGGTCTTGCTGAGTCAGGCAGGCCGACCCGGTTAAGTTTGGGGCGCTTGTCGCTTGT
         *** ** * * ***    ******* ******** * *** ** **** ******* ************* ****** ********** **

NbRZ1    CATGGACTTGACAATGAAGGGGACCGAAGTAGCCGAAAG-GGACAACTGGTCGGGGGCTAGTGCCGCTCCCCTGATGAGCCCAAGGCAGG
NbRZ2    CATGGACTTGACAATGAAGGGGACCGAAGTAGCCGAAAG-GGACAACTGGTCGGGGGCTAGTGCCGCTCCCCTGATGAGCCCAAGGCAGG
NbRZ3    CATGGACTTGACAATGAAGGGGACCGAAGTAGCCGAAAG-GGACAACTGGTCGGGGGCTAGTGCCGCTCCCCTGATGAGCCCAAGGCAGG
NbRZ4    CATGGACTTGACAATGAAGGGGACCGAAGTAGCCGAAAG-GGACAACTGGTCGGGGGCTAGTGCCGCTCCCCTGATGAGCCCAAGGCAGG
NbRZ6    CATGGACTTGACAACGAAGGGGACCAAAGTAGGCCTAAGGGATAAACTGGTCGGGGGCTAGTTCCGCTCCCCTGATGAGCCCAAGTCAGG
NbRZ5    CATGGACTTGACTATGAAGCAGACCGAAGCAATCCTAAG-GGACAACTGGTCGGGAGCTAGTGCCGCTCCCCTGATGAGCCCAAGGCAGG
NbRZ7    CTTGGAAATGACTATGAAGTTGACCGAAGCAATCCTAAG-GGACAACTGGTCGGGGGCTAGTGCCGCTCCCCTGACGAGCCCAAGGCAGG
         * ****  **** * ****  **** *** *  *  *** *   *********** ****** ************ ********* ****

NbRZ1    GCGAAACCGGTTGGCTTCCGCATGT--GGTAAGGCTGGTCT-GGGGTGGTATGGGACGCCTCA-GACGCCGCACGGGCGCTGTGCGCTGC
NbRZ2    GCGAAACCGGTTGGCTTCCGCGTGT--GGTAAGGCTGGTCT-GGGGTGGTATGGGACGCCTCA-GACGCCGCACGGGCGCTGTGCGCTGC
NbRZ3    GCGAAACCGGTTGGCTTCCGCGTGT--GGTAAGGCTGGTCT-GGGGTGGTATGGGACGCCTCA-GACGCCGCACGGGCGCTGTGCGCTGC
NbRZ4    GCGAAACCGGTTGGCTTCCGCGTGT--GGTAAGGCTGGTCT-GGGGTGGTATGGGACGCCTCA-GACGCCGCACGGGCGCTGTGCGCTGC
NbRZ6    GCAAAACCGGTTGGCTTCCGCGTGT--GGTAAGGCTTGTCTCGAGGTAGT--GGGCGGCCTCAGGACGCCGCACAGGCGCTGTGTGCTGC
NbRZ5    GCGAAACCGGTTGGCTTCCGCATGT--GGTAAGGCTGGTCTCGGGGTGGTATGGGACGCCTCGGGACGCTGCACGGGCGCTGTGTGCTGC
NbRZ7    GCGAAACCAGTTGGCATCCGCGTACACGATTAGGCTTGTCTCGGGGT---ATGGCGGGCCTTGGGACGCCGCACGGGCGCCGCACGCTGT
         ** ***** ****** ***** *     * * ***** **** * ***      **    ****     **** **** ***** *    ****

NbRZ1    GCCGGACTCTCGTCCCGTGACAAGTGGTATCAGAGCCGGACGTAC--TTGCTGTTGTTGCGCGTTTATGGTGGGG-TCCTCGGGGG-AGG
NbRZ2    GCCGGACTCTCGTCCCGTGACAAGTGGTATCAGAGCCGGACGTAC--TTGCTGTTGTTGCGCGTTTATGGTGGGG-TCCTCGGGGG-AGG
NbRZ3    GCCGGACTCTCGTCCCGTGACAAGTGGTATCAGAGCCGGACGTAC--TTGCTGTTGTTGCGCGTTTATGGTGGGG-TCCTCGGGGG-AGG
NbRZ4    GCCGGACTCTCGTCCCGTGACAAGTGGTATCAGAGCCGGACGTAC--TTGCTGTTGTTGCGCGTTTATGGTGGGG-TCCTCGGGGG-AGG
NbRZ6    GCCGGACTCTTAGCCCGTGACAATTGGTATCAGAGCGGGACGTAC--TTGCTG---CTGCGCGT-TATGGTGGGG-TCCTCAAGGG-AGG
NbRZ5    GCCGGACTCTCGGCCCGTGACAAGTGGTATCAGAGCAGGACGTAC--TTGCTGTTGTTGCGCGT-TATGGTGGGA-TCCTCAGGGG-AGG
NbRZ7    GCCGGACTCTCAGCCCGTGACAAGTGGTATCAGAGCAGGACGTACATTTGCTGGGTCTGCGCGC-TATGGTGGGGGTCCTCACAGAAAGG
         **********   ********** ************ ********  ******   ****** *********  *****   *  ***

NbRZ1    ACTGTGCGGGTGCGCGT--------------------TGCCTAGGGG------GTGGCTCGCACTGAAGACGGCCCCATCCTGCAATGGT
NbRZ2    ACTGTGCGGGTGCGCGT--------------------TGCCTAGGGG------GTGGCTCGCACTGAAGACGGCCCCATCCTGCAATGGT
NbRZ3    ACTGTGCGGGTGCGCGT--------------------TGCCTAGGGG------GTGGCTCGCACTGAAGACGGCCCCATCCTGCAATGGT
NbRZ4    ACTGTGCGGGTGCGCGT--------------------TGCCTAGGGG------GTGGCTCGCACTGAAGACGGCCCCATCCTGCAATGGT
NbRZ6    ACTGTGCGGGTGCGTTG-CCTA---------------GGCGGGGGGG------GGGGCTCGTGCCGACGACTGCCCCATCCTGCTATGGT
NbRZ5    ATTGTGCGGGTGCGTTGGCCTAAGTTTA-ATCCCGGACGGTGAGGGC---ATCGTGGCACGCACTGAAGACTGCCCCATCCTGCAA-GGT
NbRZ7    ACTGAGCGGGTGCGTTTGCCTACACACACATTCCGGATGGTGAGCGCAGCACCGTGGCACGCACTGACGACTGCCCTATCCTGCAA-GGT
         * ** *********                      *    * *        * *** **   * ** *** **** ******* * ***

NbRZ1    CTGCAGGGGCAAGACCGG--GATGGCCA-CCAGCAAGGACGGTTGCACGAGGTGAGGTGCGACTGGG-CCAAAACACGCGGCGCGCATAG
NbRZ2    CTGCAGGGGCAAGACCGG--GATGGCCA-CCAGCAAGGACGGTTGCACGAGGTGAGGTGCGACTGGG-CCAAAACACGCGGCGCGCATAG
NbRZ3    CTGCAGGGGCAAGACCGG--GATGGCCA-CCAGCAAGGACGGTTGCACGAGGTGAGGTGCGACTGGG-CCAAAACACGCGGCGCGCATAG
NbRZ4    CTGCAGGGGCAAGACCGG--GATGGCCA-CCAGCAAGGACGGTTGCACGAGGTGAGGTGCGACTGGG-CCAAAACACGCGGCGCGCATAG
NbRZ6    CTGCAGGGGAAAGACCGG--TATGGCCA-CCAGCAAGGCCGGTTGCACGAGGTGAGGTGCGATTGAG-CAAAGAGACGCGGCGCGTTAAG
NbRZ5    CAACCGGGT---GACCGGTGGATAGCTA-CCAGCAAGGCCGGTTGCACAAGGCGTGATGCGACTGAGCCAAAGGGATGCGGCGCGCAAAG
NbRZ7    CTTTCCGGAC--GACCGGTGGATGGCCACCAAGCCATGCCCGTTGC------CATCAAGTGACTGGG-CACAATCACGCGGCGCGTATAG
         *     **     ******    ** **  * * *** * * * *****                  * ** ** * *  *   * ********    **

NbRZ1    CAGTATTGTACGGGATCCTTGGGCAGAGTCCTTCTCCCCCTACAGCACGAGGGCGTGCTGCGTTTTCGGTGGGGGAGAATGTCACGACCC
NbRZ2    CAGTATTGTACGGGATCCTTGGGCAGAGTCCTTCTCCCCCTACAGCACGAGGGCGTGCTGCGTTTTCGGTGGGGGAGAATGTCACGACCC
NbRZ3    CAGTATTGTACGGGATCCTTGGGCAGAGTCCTTCTCCCCCTACAGCACGAGGGCGTGCTGCGTTTTCGGTGGGGGAGAATGTCACGACCC
NbRZ4    CAGTATTGTACGGGATCCTTGGGCAGAGTCCTTCTCCCCCTACAGCACGAGGGCGTGCTGCGTTTTCGGTGGGGGAGAATGTCACGACCC
NbRZ6    CAGTATTGTGCGGGTTCCT-GGGTGGAGTCCTTCTT-CCCAACAGCACGAGGACGTGCTGGGTTTTGGGTGGGGGAGAATGTCACGACCC
NbRZ5    CAGTATTGTACGGGATCCTTGGGCAGAGTCCTTCTC-CCCAACAGCACGAGGGCGTGCTGGATTTTGGGTGGGGGAGAATGTCACGACCC
NbRZ7    CAGCATTGTACGGGATCCTTGGGCAGAGTCCTTCTC-CCCATCAGCACGAGGACGTGCTGCGACATGGGTGGGGGAGAATGTCACGACCC
         *** ***** **** **** ***  **********  ***  ********* *******     * **********************
```

```
NbRZ1    GATTGTCGTGACCGTAAAGACCAAATGAAAGAGCGGGCCATCTGGATTTTCGTCGTTGGGAAATAAAGGGAACGGCATTAGCGATAA-GG
NbRZ2    GATTGTCGTGACCGTAAAGACCAAATGAAAGAGCGGGCCATCTGGATTTTCGTCGTTGGGAAATAAAGGGAACGGCATTAGCGATAA-GG
NbRZ3    GATTGTCGTGACCGTAAAGACCAAATGAAAGAGCGGGCCATCTGGATTTTCGTCGTTGGGAAATAAAGGGAACGGCATTAGCGATAA-GG
NbRZ4    GATTGTCGTGACCGTAAAGACCAAATGAAAGAGCGGGCCATCTGGATTTTCGTCGTTGGGAAATAAAGGGAACGGCATTAGCGATAA-GG
NbRZ6    GTTTGTCGTGATCGTGTAGTCCAAAGGAAAGAGCGGGCCATCTGGAACTTTTTCGTTGGG---TTACGGGGACGACATTAGCAATAA-GA
NbRZ5    GATTGTCGTGACTGTTAAGATCAAATGAAAGAGCGGACCATCTGGATTTTCGTTGTTGGGAAATAAAGGGAACGGCATTAGCAATAAGGG
NbRZ7    GATTGTCGTGAACGATGAGATCACATGAAAGAGCAGACCATCTGGCAATTCGTCGTTGGGAA--TAAGGGAACGGCATTAGCAATAAGGG
         * *********  *    **   ** * ******** * ********    **   * ******      * *** *** ******* **** *

NbRZ1    CGTTCTCCCTTAGATGCTTCGATCTGGCCGAGTCAGGCAGGCCGACCCGGTTAAGTTTGGGGAGCTTGTCGCTGGTCATGGACTTGACAA
NbRZ2    CGTTCTCCCTTAGATGCTTCGATCTGGCCGAGTCAGGCAGGCCGACCCGGTTAAGTTTGGGGAGCTTGTCGCTGGTCATGGACTTGACAA
NbRZ3    CGTTCTCCCTTAGATGCTTCGATCTGGCCGAGTCAGGCAGGCCGACCCGGTTAAGTTTGGGGAGCTTGTCGCTGGTCATGGACTTGACAA
NbRZ4    CGTTCTCCCTTAGATGCTTCGATCTGGCCGAGTCAGGCAGGCCGACCCGGTTAAGTTTGGGGAGCTTGTCGCTGGTCATGGACTTGACAA
NbRZ6    CGTTCTCCCTTAGATGCTTCGATCTGGCCGAGTCAGGCGGGCTGACCCGGTTAAGTTTAGGGTGCTTGTCGCTGGTCATGGACTTGACAA
NbRZ5    CGTTCTCCCTAAGATGCTTTGGTCTTGCCGAGTTAGGCAGGCCGACCCGGTTAAGTTTGGGGCGCTTGTCGCTGGTCATGGACTTGACTA
NbRZ7    CGTTCTCCCTAAGATGCTTTGGTCTTGCCGAGTCAGGCAGGCCGACCCGGTTAAGTTTGGGGCGCTTGTCGCTTGTCTTGGACATGACTA
         ********** ******** * *** ******* **** *** *************** *** ********** *** ***** **** *

NbRZ1    TGAAGGGGACCGAAGTAGCCG-AAAGGGACAACTGGTCGGGGGCTAGTGCCGCTCCCCTGATGAGCCCAAGGCAGGGCGAAACCGGTTGG
NbRZ2    TGAAGGGGACCGAAGTAGCCG-AAAGGGACAACTGGTCGGGGGCTAGTGCCGCTCCCCTGATGAGCCCAAGGCAGGGCGAAACCGGTTGG
NbRZ3    TGAAGGGGACCGAAGTAGCCG-AAAGGGACAACTGGTCGGGGGCTAGTGCCGCTCCCCTGATGAGCCCAAGGCAGGGCGAAACCGGTTGG
NbRZ4    TGAAGGGGACCGAAGTAGCCG-AAAGGGACAACTGGTCGGGGGCTAGTGCCGCTCCCCTAATGAGCCCAAGGCAGGGCGAAACCGGTTGG
NbRZ6    TGAAGGGGACCGAAGTAAGCCTAAAGGATCAACTGGTCGGGGGCTAGTGCCGCTCCCCTAATGAGCCCAAGGCAAGGCGAAACCGGTTGG
NbRZ5    TGAAGCAGACCGAAGCAATCC-TAAGGGACAACTGGTCGGGAGCTAGTGCCGCTCCCCTGATGAGCCCAAGGCAGGGCGAAACCGGTTGG
NbRZ7    TGAAGTTGACCGAAGCAATCC-TAAGGGACAACTGGTCGGGGGCTAGTGCCGCTCCCCTGACGAGCCCAAGGCAGGGCGAAACCAGTTGG
         *****  ******** *  *    ****   ************ **************** * ************ ********* *****

NbRZ1    CTTCCGCGTGT--GGTAAGGCTGGTCTGGGGTGGTATGG-GACGCCTC-AGACGCCGCACGGGCGCTGTGCGCTGCGCCGGACTCTCGTC
NbRZ2    CTTCCGCGTGT--GGTAAGGCTGGTCTGGGGTGGTATGG-GACGCCTC-AGACGCCGCACGGGCGCTGTGCGCTGCGCCGGACTCTCGTC
NbRZ3    CTTCCGCATGT--GGTAAGGCTGGTCTGGGGTGGTATGG-GACGCCTC-AGACGCCGCACGGGCGCTGTGCGCTGCGCCGGACTCTCGGC
NbRZ4    CTTCCGCATGT--GGTAAGGCTGGTCTGGGGTGGTATGG-GACGCCTC-AGACGCCGTACGGGCGCTGTGCGCTGCGCCGGACTCTCGGC
NbRZ6    CTTCCGCGTAT--GGTAAGGCTTGTCTCGAGGTAGTGGG--CTGCCTCGAGACGCTGCACGGGCGCTGTGTGCTGCGCCGAACTCTCAGC
NbRZ5    CTTCCGCATGT--GGTAAGGCTGGTCTCAGGGTGGTATGGGACGCCTCGGGACACCGCACGGGCGCTGTGTGCTGCGCCGGACTCTCGGC
NbRZ7    CATCCGCGTACACGATTAGGCTTGTCTCGTGGT--ATGGCGGGCCTCGGGACGCCGCACGGGCGCCACACGCTGCGCCGGACTCTCAGC
         * ***** *     * * ***** ****    *        *     *****   *** * * ********     ********* ******  *

NbRZ1    CCGTGACA
NbRZ2    CCGTGACA
NbRZ3    CCGTGACA
NbRZ4    CCGTGACA
NbRZ6    CCGTGACA
NbRZ5    CCGTGACA
NbRZ7    CCGTGACA
         ********
```

Figure S3. Sequence alignment of *N. benthamiana* Group 1 retrozymes. In the NbRZ1 sequence, the LTR regions are marked with yellow color and the ribozyme sequences are shown in bold. Asterisks show invariant residues. Gray shading indicates differences from the NbRZ1 sequence. Blue color denotes a region forming a stem in the NbRZ1 predicted secondary structure and containing insertions in NbRZ5, NbRZ6, and NbRZ7. This region was selected as a site for insertion of an artificial tag sequence into NbRZ1.

```
NbRZ8    TGTCACGTCCTCAGAGAAGGGGCAAAGTAAAGCGTGCGGATTGGCATCAAACTGCCTTGGAGAGGGGAGGCGTCTCTAGCCATTAAGGCG
NbRZ9    TGTCACGGCCTCAGAGTAGGGACAAAGAAAAGCGTGCGGATTGGCATCAAACTGCCTTGGGGAGGGGAGGTGCCTCTAGCCATAAAGGCG
         ******* ******** **** ***** ******************************** ********* * ********** ******

NbRZ8    CTCTGCCCTAGTTGCGTATTGGCTTGGCCGAGTTAGGCATGCCGACCCGATTAAGTTTGGGGAGCATGTCGCTTGTCTTGGACATGACTA
NbRZ9    CTCTGCCCTAGTTGCGTATTGGCTTGGCCGAGTTAGGCATGCCGACCCGATTAAGTTTGGGGAGCATGTCGCTTGTCTTGGACATGACTA
         ******************************************************************************************

NbRZ8    TGAAGGGAGCCGAAGCATACGAGGGGCACAGCTGGTCGGGAGCTATTGCCGCTTCCCTGATGAGCCCATGGCAGGGCGAAACCAGTCTGC
NbRZ9    TGAAGGGAGCCGAAGCATACGGGGGGCACAGCTGGTCGGGAGCTATTGCCGCTTCCCTGATGAGCCCATGGCAGGGCGAAACCAGTCTGC
         ********************* ********************************************************************

NbRZ8    GCGCACAGGCAGGACGGGCGTGTCCGGGGCGG-TATGGGACGCCTCGGGCGCGGAGCGCCGCGGTCCGTGTCATTTGGTATCAGAGCAAA
NbRZ9    GCGCT-AGGTCGGACGGGCGTGTCCGGGGCGGTTATGGGACGCCTCGGGCGCGAAGCGCCGCGGTCCGTCACATTTGGTATCAGAGCAAA
         ****  **** ********************* ******************** ***************  *******************

NbRZ8    CATGCTCTTCAACTTCTGTGGCGAGGTGTCAAGCTCATGAATGCACAAAGCTAGCG-GAAGGC-ATAAGTGCAAAGATGGGGGCGCTGAG
NbRZ9    CATGCTCTTCAACTTCTCTTGCAAGGTGTCA-GCTCATGAATGCACAA-GCTCGGGTGAATGCTACAAGTGCAAAGATGGGGGCGCTGAG
         ***************** * ** ******** **************** *** * * *** ** * ************************

NbRZ8    TTACAGAGTGGGGCATGACGGTCCTTGGGGCAAACATACTACTTCTCCGCCAAAGTAAAGCGGGCTTGAGCAAATGAAATTGTTGCGAAC
NbRZ9    TGACAGAGTGGAGCATGACGGTCCTTGG-GCAAACATACTACTTCTCCGCCAAAGTAAAGCGGGCTTGAGC-AATGAAATTGTTGCGAAC
         * ********* **************** ****************************************** ******************

NbRZ8    TCTAGATGAAACCACGAAGTGGACGCGCCGACGAAGTGTGAAGC--TCTCCATGGTTTCGTGCCTAAGTCCGAG-GTAGGCAGCACGAGG
NbRZ9    TCTAGATGAAACCACGAAGTGGACGCACCGACGAAGTGTGATGCCGTCTCCATGATTACGTGCCTGAGTCCCAAAGTTGGCAGCACGAGG
         ************************** ************** **  ******** ** ******* ***** *  ** ************

NbRZ8    AGGTA----GCTAGCATGA---TGGGTGCAATGTTCCGAGTGGAAGACGAAAGGAGTAGCAGCGAAAGTAATGCGTCGGGTCGCCGCATC
NbRZ9    AGGTGTGGTGCAAGCATGAATGAGGGTGCAA-GTTCCGTGTGGAAGACGAAAGGAGTTGCAGCAAAATTAATGCGTCGGGTCGCCGCATC
         ****     ** *******    ******** ****** ***************** ****** *** **********************

NbRZ8    AGCCAGGTGGGGTAGGGTTGTCACGTCCTCAGAGAAGGGGCAAAGTAAAGCGTGCGGATTGGCATCAAACTGCCTTGGAGAGGGGAGGCG
NbRZ9    ATCG-GGTGGGGGAGAGTTGTCAAGGCCTCAGAGCAGGGACAAAGTAAAGCGTGCGGATTGGCATCAAACTGTCTTGGGAAGGAGAGGTG
         * *  ******* ** ******* * ******** **** ******************************** *****  *** **** *

NbRZ8    TCTCTAGCCATTAAGGCGCTCTGCCCTAGTTGCGTATTGGCTTGGCCGAGTTAGGCATGCCGACCCGATTAAGTTTGGGGAGCATGTCGC
NbRZ9    CCTCTAGCCATAAAGGCGCTCTGCCCTAGTTGCGTATTGGCTTGGCCGAATTAGGCATGCCGACCCGATTTAGTTTGGGGAGCATATCGC
          ********** ************************************* ******************** ************** ****

NbRZ8    TTGTCTTGGACATGACTATGAAGGGAGCCGAAGCATACGAGGGGCACAGCTGGTCGGGAGCTATTGCCGCTTCCCTGATGAGCCCATGGC
NbRZ9    TTGACTTGGACATGACTATGAAGGGAGCCGAAGCATACGGGGGGCAGAGCTGGTCGGGAGCTATTGCCGCTTCCCTGATGAGCCCATGGC
         *** *********************************** ****** *******************************************

NbRZ8    A-GGGCGAAACCAGTCTGCGCGCACAGGCAGGACGGGCGTGTCCGGGGCGG-TATGGGACGCCTCGGGCGCGGAGCGCCGCGGTCCGTGTCA
NbRZ9    AGGGGCGAAACCAGTCTGCGCGCT-AGGTCGGACGAGCGTGTCCGCGGCGGTTATGAGACGCCTCGGGCGCGAAGCGCCGCGGTCCGTCACA
         * *********************  ***  ***** ********* ***** **** *************** ***************  **
```

Figure S4. Sequence alignment of *N. benthamiana* Group 2 retrozymes. In the NbRZ8 sequence, LTR regions are marked with yellow color and ribozyme sequences are shown in bold. Asterisks indicate invariant residues.

<pre>
NbRZ1   CGATCTGGCCGAGTCAGGCAGGCCGACCCGGTTAAGTTTGGGGAGCTTGTCGCTGGTCATGGACTTGACAATGAAGGGGACCGAAGTAGC
NbRZ2   CGATCTGGCCGAGTCAGGCAGGCCGACCCGGTTAAGTTTGGGGAGCTTGTCGCTGGTCATGGACTTGACAATGAAGGGGACCGAAGTAGC
NbRZ3   CGATCTGGCCGAGTCAGGCAGGCCGACCCGGTTAAGTTTGGGGAGCTTGTCGCTGGTCATGGACTTGACAATGAAGGGGACCGAAGTAGC
NbRZ4   CGATCTGGCCGAGTCAGGCAGGCCGACCCGGTTAAGTTTGGGGAGCTTGTCGCTGGTCATGGACTTGACAATGAAGGGGACCGAAGTAGC
NbRZ5   TGGTCTTGCCGAGTTAGGCAGGCCGACCCGGTTAAGTTTGGGGTGCTTGTCGCTGGTCATGGACTTGACTATGAAGCAGACCGAAGCAAT
NbRZ6   CGATCTGGCCGAGTCAGGCAGGTCGACCCGGTTAAGCTTGGGGTGCTTGTCGCTGGTCATGGACTTGACAACGAAGGGGACCAAAGTAGG
NbRZ7   TGGTCTTGCTGAGTCAGGCAGGCCGACCCGGTTAAGTTTGGGGCGCTTGTCGCTTGTCTTGGAAATGACTATGAAGTTGACCGAAGCAAT
NbRZ8   TGGCTTGGCCGAGTTAGGCATGCCGACCCGATTAAGTTTGGGGAGCATGTCGCTTGTCTTGGACATGACTATGAAGGGAGCCGAAGCATA
NbRZ9   TGGCTTGGCCGAGTTAGGCATGCCGACCCGATTAAGTTTGGGGAGCATGTCGCTTGTCTTGGACATGACTATGAAGGGAGCCGAAGCATA
          *   * ** **** ***** * ******* ***** ****** ** ******* *** **** **** * ****   ** *** *

NbRZ1   CGAAAG-GGACAACTGGTCGGGGGCTAGTGCCGCTCCCCTGATGAGCCCAAGGCAGGGCGAAACCGGTTGGCTTCC
NbRZ2   CGAAAG-GGACAACTGGTCGGGGGCTAGTGCCGCTCCCCTGATGAGCCCAAGGCAGGGCGAAACCGGTTGGCTTCC
NbRZ3   CGAAAG-GGACAACTGGTCGGGGGCTAGTGCCGCTCCCCTGATGAGCCCAAGGCAGGGCGAAACCGGTTGGCTTCC
NbRZ4   CGAAAG-GGACAACTGGTCGGGGGCTAGTGCCGCTCCCCTGATGAGCCCAAGGCAGGGCGAAACCGGTTGGCTTCC
NbRZ5   CCTAAG-GGACAACTGGTCGGGAGCTAGTGCCGCTCCCCTGATGAGCCCAAGGCAGGGCGAAACCGGTTGGCTTCC
NbRZ6   CCTAAGGGATAAACTGGTCGGGGGCTAGTTCCGCTCCCCTGATGAGCCCAAGTCAGGGCAAAACCGGTTGGCTTCC
NbRZ7   CCTAAG-GGACAACTGGTCGGGGGCTAGTGCCGCTCCCCTGACGAGCCCAAGGCAGGGCGAAACCAGTTGGCATCC
NbRZ8   CGAGGG-GCACAGCTGGTCGGGAGCTATTGCCGCTTCCCTGATGAGCCCATGGCAGGGCGAAACCAGTCTGCGCGC
NbRZ9   CGGGGG-GCACAGCTGGTCGGGAGCTATTGCCGCTTCCCTGATGAGCCCATGGCAGGGCGAAACCAGTCTGCGCGC
         *   * *   * ********* **** * ***** ****** ******* * ****** ***** ** **   *
</pre>

Figure S5. Conserved region in the LTRs of Group 1 and Group 2 retrozymes. Sequence alignment of region conserved in LTRs of Group 1 and Group 2 retrozymes is presented. Ribozyme sequences are shown in bold. Asterisks indicate invariant residues.

A

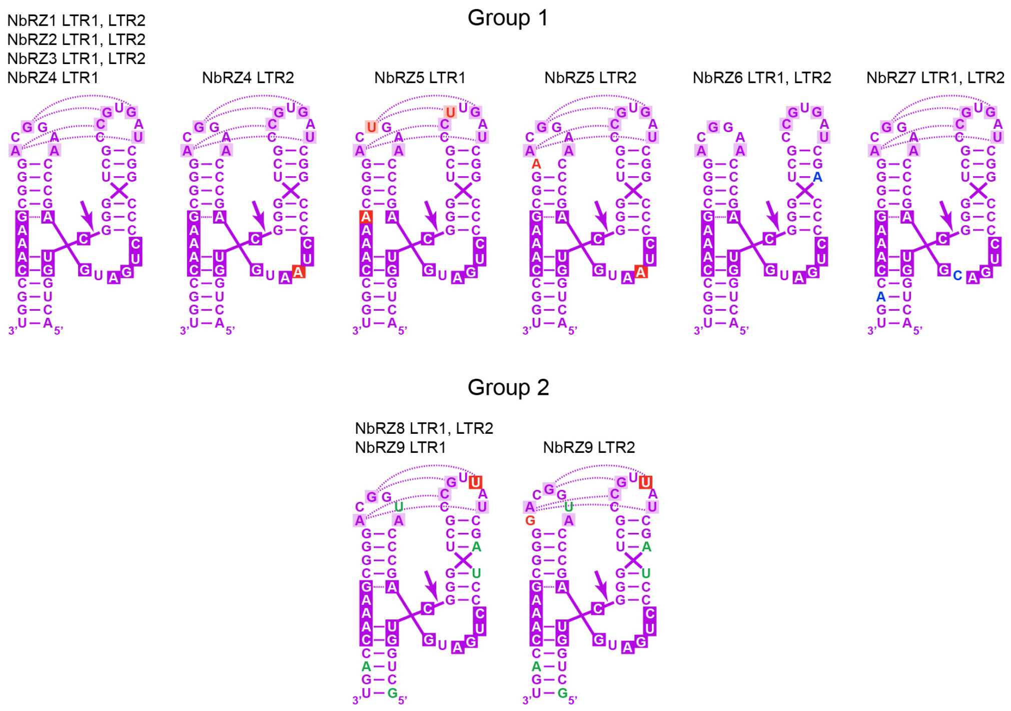

B

Group	Retrozyme	Mismatches between two LTRs
Group 1	NbRZ1	1
	NbRZ2	0
	NbRZ3	2
	NbRZ4	7
	NbRZ5	9
	NbRZ6	32
	NbRZ7	11
Group 2	NbRZ8	0
	NbRZ9	15

Figure S6. Analysis of potential ribozyme structures in *N. benthamiana* retrozymes. (**A**) Predicted structure of ribozymes in two LTR regions of NbRZ1-NbRZ9. For Group 1 retrozymes, substitutions potentially affecting the ribozyme structure/function are shown in red color, those having no effect - in blue color. In NbRZ4 and NbRZ5, the ribozyme sequences did not match in two LTRs, with mutations being located at essential residues, and NbRZ5 ribozyme contained as many as five substitutions in two LTRs. Therefore, potential transcripts of NbRZ4 and NbRZ5 might be incapable of self-cleavage at both LTRs. For Group 2 retrozymes, substitutions affecting non-essential residues and preserving base pairing of the retrozyme fold are shown in green, a substitution of residue involved in non-canonical interactions of two retrozyme loops is shown in red. (**B**) Numbers of mismatches between two LTRs of *N. benthamiana* retrozymes.

GTAATACGACTCACTATAGGGCAAGCAGTGGTATCAACGCAGAGTACGCGGGGGGGGCTAGTGCCGCTCCCCTGATGAGCCCA
AGGCAGGGCGAAACCGGTTGGCTTCCGCATGTGGTAAGGCTGGTCTGGGGTGGTATGGGACGCCTCAGACGCCGCACGGGCGC
TGTGCGCTGCGCCGGACTCTCGTCCCGTGACA**CCATGG**TGAGTAAAGGAGAAGAACTTTTCACTGGAGTTGTCCCAATTCTTG
TTAAATTAGATGGTGATGTTAATGGGCACAAATTTTCTGTCAGTGGAGAGGGTGAAGGTGATGCTACATACGGAAAGCTTACC
CTTAAATTTATTTGCACTACTGGAAAACTACCTGTTCCTTGGCCAACACTTGTCACTACTTTCTCTTATGGTGTTCAATGCTT
TTCCCGTTATCCGGATCATATGAAACGGCATGACTTTTTCAAGAGTGCCATGCCCGAAGGTTATGTACAGGAACGCACTATAT
CTTTCAAAGATGACGGGAACTACAAGACACGTGCTGAAGTCAAGTTTGAAGGTGATACCCTTGTTAATCGTATCGAGTTAAAA
GGTATTGATTTTAAAGAAGATGGAAACATTCTCGGACACAAACTGGAGTACAACTATAA

Figure S7. 5’-RACE analysis of LTR-GFP transcription product. Sequence of 5’-RACE product obtained on RNA from *N. benthamiana* leaves infiltrated with agrobacteria carrying the LTR-GFP construct. Gray color indicates a sequence derived from 5’-RACE adaptor and amplification primers, yellow color - the LTR sequence, green color - the GFP sequence. *NcoI* site used for cloning is shown in bold.

```
NbRZ1          TTGTACGGGATCCTTGGGCAGAGTCCTTCTCCCCCTACAGCACGAGGGCGTGCTGCGTTTTCGGTGGGGGAGAATGTCAC
NbRZ2          TTGTACGGGATCCTTGGGCAGAGTCCTTCTCCCCCTACAGCACGAGGGCGTGCTGCGTTTTCGGTGGGGGAGAATGTCAC
NbRZ3          TTGTACGGGATCCTTGGGCAGAGTCCTTCTCCCCCTACAGCACGAGGGCGTGCTGCGTTTTCGGTGGGGGAGAATGTCAC
NbRZ4          TTGTACGGGATCCTTGGGCAGAGTCCTTCTCCCCCTACAGCACGAGGGCGTGCTGCGTTTTCGGTGGGGGAGAATGTCAC
lx1-423        TTGTACGGGATCCTTGGGCAGAGTCCTTCTCCCCCTACAGCACGAGGGCGTGCTGCGTTTTCGGTGGGGGAGAATGTCAC
2RC-425        TTGTACGGGATCCTTGGGCAGAGTCCTTCTCCCCCTACAGCACGAGGGCGTGCTGCGTTTTCGGTGGGGGAGAATGTCAC
4RC-425        TTGTACGGGATCCTTGGGCAGAGTCCTTCTCCCCCTACAGCACGAGGGCGTGCTGCGTTTTCGGTGGGGGAGAATGTCAC
lx2-423        TTGTACGGGATCCTTGGGCAGAGTCCTTCTCCCCCTACAGCACGAGGGCGTGCTGCGTTTTCGGTGGGGGAGAATGTCAC
lx3RC-423      TTGTACGGGATCCTTGGGCAGAGTCCTTCTCCCCCTACAGCACGAGGGCGTGCTGCGTTTTCGGTGGGGGAGAATGTCAC
lx4RC-423      TTGTACGGGATCCTTGGGCAGAGTCCTTCTCCCCCTACAGCACGAGGGCGTGCTGCGTTTTCGGTGGGGGAGAATGTCAC
5-425          TTGTACGGGATCCTTGGGCAGAGTCCTTCTCCCCCTACAGCACGAGGGCGTGCTGCGTTTTCGGTGGGGGAGAATGTCAC
1RC-425        TTGTACGGGATCCTTGGGCAGAGTCCTTCTCCCCCTACAGCACGAGGGCGTGCTGCGTTTTCGGTGGGGGAGAATGTCAC
               ********************************************************************************

NbRZ1          GACCCGATTGTCGTGACCGTAAAGACCAAATGAAAGAGCGGGCCATCTGGATTTTCGTCGTTGGGAAATAAAGGGAACGG
NbRZ2          GACCCGATTGTCGTGACCGTAAAGACCAAATGAAAGAGCGGGCCATCTGGATTTTCGTCGTTGGGAAATAAAGGGAACGG
NbRZ3          GACCCGATTGTCGTGACCGTAAAGACCAAATGAAAGAGCGGGCCATCTGGATTTTCGTCGTTGGGAAATAAAGGGAACGG
NbRZ4          GACCCGATTGTCGTGACCGTAAAGACCAAATGAAAAAGCGTGCCATCTGGATTTTCGTCGTTGGGAAATAAAGGGAACGA
lx1-423        GACCCGATTGTCGTGACCGTAAAGACCAAATGAAAGAGCGGGCCATCTGGATTTTCGTCGTTGGGAAATAAAGGGAACGG
2RC-425        GACCCGATTGTCGTGACCGTAAAGACCAAATGAAAGAGCGGGCCATCTGGATTTTCGTCGTTGGGAAATAAAGGGAACGG
4RC-425        GACCCGATTGTCGTGACCGTAAAGACCAAATGAAAGAGCGGGCCATCTGGATTTTCGTCGTTGGGAAATAAAGGGAACGG
lx2-423        GACCCGATTGTCGTGACCGTAAAGACCAAATGAAAGAGCGGGCCATCTGGATTTTCGTCGTTGGGAAATAAAGGGAACGG
lx3RC-423      GACCCGATTGTCGTGACCGTAAAGACCAAATGAAAGAGCGGGCCATCTGGATTTTCGTCGTTGGGAAATAAAGGGAACGG
lx4RC-423      GACCCGATTGTCGTGACCGTAAAGACCAAATGAAAGAGCGGGCCATCTGGATTTTCGTCGTTGGGAAATAAAGGGAACGG
5-425          GACCCGATTGTCGTGACCGTAAAGACCAAATGAAAGAGCGGGCCATCTGGATTTTCGTCGTTGGGAAATAAAGGGAACGG
1RC-425        GACCCGATTGTCGTGACCGTAAAGACCAAATGAAAGAGCGGGTCATCTGGATTTTCGTCGTTGGGAAATAAAGGGAACGG
               *********************************** **** * ************************************ 

NbRZ1          CATTAGCGATAAGGCGTTCTCCCTTAGATGCTTCGATCTGGCCGAGTCAGGCAGGCCGACCCGGTTAAGTTTGGGGAGCT
NbRZ2          CATTAGCGATAAGGCGTTCTCCCTTAGATGCTTCGATCTGGCCGAGTCAGGCAGGCCGACCCGGTTAAGTTTGGGGAGCT
NbRZ3          CATTAGCGATAAGGCGTTCTCCCTTAGATGCTTCGATCTGGCCGAGTCAGGCAGGCCGACCCGGTTAAGTTTGGGGAGCT
NbRZ4          CATTAGCGATAAGGCGTTCTCCCTTAGATGCTTCGATCTGGCCGAGTCAGGCAGGCCGACCCGGTTAAGTTTGGGGAGCT
lx1-423        CATTAGCGATAAGGCGTTCTCCCTTAGATGCTTCGATCTGGCCGAGTCAGGCAGGCCGACCCGGTTAAGTTTGGGGAGCT
2RC-425        CATTAGCGATAAGGCGTTCTCCCTTAGATGCTTCGATCTGGCCGAGTCAGGCAGGCCGACCCGGTTAAGTTTGGGGAGCT
4RC-425        CATTAGCGATAAGGCGTTCTCCCTTAGATGCTTCGATCTGGCCGAGTCAGGCAGGCCGACCCGGTTAAGTTTGGGGAGCT
lx2-423        CATTAGCGATAAGGCGTTCTCCCTTAGATGCTTCGATCTGGCCGAGTCAGGCAGGCCGACCCGGTTAAGTTTGGGGAGCT
lx3RC-423      CATTAGCGATAAGGCGTTCTCCCTTAGATGCTTCGATCTGGCCGAGTCAGGCAGGCCGACCCGGTTAAGTTTGGGGAGCT
lx4RC-423      CATTAGCGATAAGGCGTTCTCCCTTAGATGCTTCGATCTGGCCGAGTCAGGCAGGCCGACCCGGTTAAGTTTGGGGAGCT
5-425          CATTAGCGATAAGGCGTTCTCCCTTAGATGCTTCGATCTGGCCGAGTCAGGCAGGCCGACCCGGTTAAGTTTGGGGAGCT
1RC-425        CATTAGCGATAAGGCGTTCTCCCTTAGATGCTTCGATCTGGCCGAGTCAGGCAGGCCGACCCGGTTAAGTTTGGGGAGCT
               ********************************************************************************

NbRZ1          TGTCGCTGGTCATGGACTTGACAATGAAGGGGACCGAAGTAGCCGAAAGGGACAACTGGTCGG-GGGCTAGTGCCGCTCC
NbRZ2          TGTCGCTGGTCATGGACTTGACAATGAAGGGGACCGAAGTAGCCGAAAGGGACAACTGGTCGG-GGGCTAGTGCCGCTCC
NbRZ3          TGTCGCTGGTCATGGACTTGACAATGAAGGGGACCGAAGTAGCCGAAAGGGACAACTGGTCGG-GGGCTAGTGCCGCTCC
NbRZ4          TGTCGCTGGTCATGGACTTGACAATGAAGGGGACCGAAGTAGCCGAAAGGGACAACTGGTCGG-GGGCTAGTGCCGCTCC
lx1-423        TGTCGCTGGTCATGGACTTGACAATGAAGGGGACCGAAGTAGCCGAAAGGGACAACTGGTCGGGGGGCTAGTGCCGCTCC
2RC-425        TGTCGCTGGTCATGGACTTGACAATGAAGGGGACCGAAGTAGCCGAAAGGGACAACTGGTCGGGGGGCTAGTGCCGCTCC
4RC-425        TGTCGCTGGTCATGGACTTGACAATGAAGGGGACCGAAGTAGCCGAAAGGGACAACTGGTCGGGGGGCTAGTGCCGCTCC
lx2-423        TGTCTCTGGTCATGGACTTGACAATGAAGGGGACCGAAGTAGCCGAAAGGGACAACTGGTCGG-GGGCTAGTGCCGCTCC
lx3RC-423      TGTCTCTGGTCATGGACTTGACAATGAAGGGGACCGAAGTAGCCGAAAGGGACAACTGGTCGG-GGGCTAGTGCCGCTCC
lx4RC-423      TGTCTCTGGTCATGGACTTGACAATGAAGGGGACCGAAGTAGCCGAAAGGGACAACTGGTCGG-GGGCTAGTGCCGCTCC
5-425          TGTCTCTGGTCATGGACTTGACAATGAAGGGGACCGAAGTAGCCGAAAGGGACAACTGGTCGG-GGGCTAGTGCCGCTCC
1RC-425        TGTCTCTGGTCATGGACTTGACAATGAAGGGGACCGAAGTAGCCGAAAGGGACAACTGGTCGG-GGGCTAGTGCCGCTCC
               **** ********************************************************** ****************

NbRZ1          CCTGATGAGCCCAAGGCAGGGCGAAACCGGTTGGCTTCCGCATGTGGTAAGGCTGGTCTGGGGTGGTATGGGACGCCTCA
NbRZ2          CCTGATGAGCCCAAGGCAGGGCGAAACCGGTTGGCTTCCGCGTGTGGTAAGGCTGGTCTGGGGTGGTATGGGACGCCTCA
NbRZ3          CCTGATGAGCCCAAGGCAGGGCGAAACCGGTTGGCTTCCGCGTGTGGTAAGGCTGGTCTGGGGTGGTATGGGACGCCTCA
NbRZ4          CCTGATGAGCCCAAGGCAGGGCGAAACCGGTTGGCTTCCGCGTGTGGTAAGGCTGGTCTGGGGTGGTATGGGACGCCTCA
lx1-423        CCTGATGAGCCCAAGACAGGGCGAAACCGGTTGGCTTCCGCATGTGGTAAGGCTGGTCTGGGGTGGTATGGGACACCTCA
2RC-425        CCTGATGAGCCCAAGACAGGGCGAAACCGGTTGGCTTCCGCATGTGGTAAGGCTGGTCTGGGGTGGTATGGGACACCTCA
4RC-425        CCTGATGAGCCCAAGACAGGGCGAAACCGGTTGGCTTCCGCATGTGGTAAGGCTGGTCTGGGGTGGTATGGGACACCTCA
lx2-423        CCTGATGAGCCCAAGGCAGGGCGAAACCGGTTGGCTTCCGCATGTGGTAAGGCTGGTCTGGGGTGGTATGGGACGCCTCA
lx3RC-423      CCTGATGAGCCCAAGGCAGGGCGAAACCGGTTGGCTTCCGCATGTGGTAAGGCTGGTCTGGGGTGGTATGGGACGCCTCA
lx4RC-423      CCTGATGAGCCCAAGGCAGGGCGAAACCGGTTGGCTTCCGCATGTGGTAAGGCTGGTCTGGGGTGGTATGGGACGCCTCA
5-425          CCTGATGAGCCCAAGGCAGGGCGAAACCGGTTGGCTTCCGCATGTGGTAAGGCTGGTCTGGGGTGGTATGGGACGCCTCA
1RC-425        CCTGATGAGCCCAAGGCAGGGCGAAACCGGTTGGCTTCCGCATGTGGTAAGGCTGGTCTGGGGTGGTATGGGACGCCTCA
               ************** ************************** ******************************** *****
```

```
NbRZ1          GACGCCGCACGGGCGCTGTGCGCTGCGCCGGACTCTCGTCCCGTGACAAGTGGTATCAGAGCCGGACGTACTTGCTGTTG
NbRZ2          GACGCCGCACGGGCGCTGTGCGCTGCGCCGGACTCTCGTCCCGTGACAAGTGGTATCAGAGCCGGACGTACTTGCTGTTG
NbRZ3          GACGCCGCACGGGCGCTGTGCGCTGCGCCGGACTCTCGTCCCGTGACAAGTGGTATCAGAGCCGGACGTACTTGCTGTTG
NbRZ4          GACGCCGCACGGGCGCTGTGCGCTGCGCCGGACTCTCGTCCCGTGACAAGTGGTATCAGAGCCGGACGTACTTGCTGTTG
lx1-423        GACGCCGCACGGGCGCTGTGCGCTGCGCCGGACTCTCGGCCCGTGACAAGTGGTATCAGAGCCGGACGTACTTGCTGTTG
2RC-425        GACGCCGCACGGGCGCTGTGCGCTGCGCCGGACTCTCGGCCCGTGACAAGTGGTATCAGAGCCGGACGTACTTGCTGTTG
4RC-425        GACGCCGCACGGGCGCTGTGCGCTGCGCCGGACTCTCGGCCCGTGACAAGTGGTATCAGAGCCGGACGTACTTGCTGTTG
lx2-423        GACGCCGCACGGGCGCTGTGCTCTGCGCCGGACTCTCGGCCCGTGACAAGTAGTATCAGAGCCGGACGTACTTGCTGTTG
lx3RC-423      GACGCCGCACGGGCGCTGTGCTCTGCGCCGGACTCTCGGCCCGTGACAAGTAGTATCAGAGCCGGACGTACTTGCTGTTG
lx4RC-423      GACGCCGCACGGGCGCTGTGCTCTGCGCCGGACTCTCGGCCCGTGACAAGTAGTATCAGAGCCGGACGTACTTGCTGTTG
5-425          GACGCCGCACGGGCGCTGTGCTCTGCGCCGGACTCTCGGCCCGTGACAAGTAGTATCAGAGCCGGACGTACTTGCTGTTG
1RC-425        GACGCCGCACGGGCGCTGTGCTCTGCGCCGGACTCTCGGCCCGTGACAAGTAGTATCAGAGCCGGACGTACTTGCTGTTG
               ********************* **************** ************ ****************************

NbRZ1          TTGCGCGTTTATGGTGGGGTCCTCGGGGGAGGACTGTGCGGGTGCGCGTTGCCTAGGGGGTGGCTCGCACTGAAGACGGC
NbRZ2          TTGCGCGTTTATGGTGGGGTCCTCGGGGGAGGACTGTGCGGGTGCGCGTTGCCTAGGGGGTGGCTCGCACTGAAGACGGC
NbRZ3          TTGCGCGTTTATGGTGGGGTCCTCGGGGGAGGACTGTGCGGGTGCGCGTTGCCTAGGGGGTGGCTCGCACTGAAGACGGC
NbRZ4          TTGCGCGTTTATGGTGGGGTCCTCGGGGGAGGACTGTGCGGGTGCGCGTTGCCTAGGGGGTGGCTCGCACTGAAGACGGC
lx1-423        TTGCGCGTTTATGGTGGGGTCCTCGGGGGAGGACTGTGCGGGT--GCGTTGCCTAGGGGGTGGCTCGCACTGAAGACGGC
2RC-425        TTGCGCGTTTATGGTGGGGTCCTCGGGGGAGGACTGTGCGGGT--GCGTTGCCTAGGGGGTGGCTCGCACTGAAGACGGC
4RC-425        TTGCGCGTTTATGGTGGGGTCCTCGGGGGAGGACTGTGCGGGT--GCGTTGCCTAGGGGGTGGCTCGCACTGAAGACGGC
lx2-423        TTGCGCGTTTATGGTGGGGTCCTCGGGGGAGGACTGTGCGGGT--GCGTTGCCTAGGGGGTGGCTCGCACTGAAGACGGC
lx3RC-423      TTGCGCGTTTATGGTGGGGTCCTCGGGGGAGGACTGTGCGGGT--GCGTTGCCTAGGGGGTGGCTCGCACTGAAGACGGC
lx4RC-423      TTGCGCGTTTATGGTGGGGTCCTCGGGGGAGGACTGTGCGGGT--GCGTTGCCTAGGGGGTGGCTCGCACTGAAGACGGC
5-425          TTGCGCGTTTATGGTGGGGTCCTCGGGGGAGGACTGTGCGGGT--GCGTTGCCTAGGGGGTGGCTCGCACTGAAGACGGC
1RC-425        TTGCGCGTTTATGGTGGGGTCCTCGGGGGAGGACTGTGCGGGT--GCGTTGCCTAGGGGGTGGCTCGCACTGAAGACGGC
               *******************************************  ***********************************

NbRZ1          CCCATCCTGCAATGGTCTGCAGGGGCAAG
NbRZ2          CCCATCCTGCAATGGTCTGCAGGGGCAAG
NbRZ3          CCCATCCTGCAATGGTCTGCAGGGGCAAG
NbRZ4          CCCATCCTGCAATGGTCTGCAGGGGCAAG
lx1-423        CCCATCCTGCAATGGTCTGCAGGGGCAAG
2RC-425        CCCATCCTGCAATGGTCTGCAGGGGCAAG
4RC-425        CCCATCCTGCAATGGTCTGCAGGGGCAAG
lx2-423        CCCATCCTGCAATGGTCTGCAGGGGCAAG
lx3RC-423      CCCATCCTGCAATGGTCTGCAGGGGCAAG
lx4RC-423      CCCATCCTGCAATGGTCTGCAGGGGCAAG
5-425          CCCATCCTGCAATGGTCTGCAGGGGCAAG
1RC-425        CCCATCCTGCAATGGTCTGCAGGGGCAAG
               *****************************
```

Figure S8. Sequence analysis of Group 1 retrozyme (-) strand-specific amplification product obtained with divergent primers. Sequences of eight independent clones were aligned with the corresponding region of NbRZ1-NbRZ4. Mismatches are shown by gray shading.

A

```
NbRZ1-Tag      GATTACAAGGATGACGATGACAAGGGGTGGCTCGCACTGAAGACGGCCCCATCCTGCAATGGTCTGCAGGGGCAAGACCG
1RC_426        GATTACAAGGATGACGATGACAAGGGGAGGCTCGCACTAAAGACGGCCCCATCCTGCAATGGTCTGCAGGGGCAAGACCG
2RC_426        GATTACAAGGATGACGATGACAAGGGGTGGCTCGCACTAAAGACGGCCCCATCCTGCAATGGTCTGCAGGGGCAAGACCG
1RC_430        GATTACAAGGATGACGATGACAAGGGGTGGCTCGCACTAAAGACGGCCCCATCCTGCAATGGTCTGCAGGGGCAAGACCG
3RC_430        GATTACAAGGATGACGATGACAAGGGGTGGCTCGCACTAAAGACGGCCCCATCCTGCAATGGTCTGCAGGGGCAAGACCG
               *************************** ********** *****************************************

NbRZ1-Tag      GGATGGCCACCAGCAAGGACGGTTGCACGAGGTGAGGTGCGACTGGGCCAAAACACGCGGCGCGCATAGCAGTATTGTAC
1RC_426        GGATGGCCACCAGCAAGGACGGTTGCACGAGGTGAGGTGCGACTGGGCCAAAACACGCGGCGCGCATAGCAGTATTGTAC
2RC_426        GGATGGCCACCAGCAAGGACGGTTGCACGAGGTGAGGTGCGACTGGGCCAAAACACGCGGCGCGCATAGCAGTATTGTAC
1RC_430        GGATGGCCACCAGCAAGGACGGTTGCACGAGGTGAGGTGCGACTGGGCCAAAACACGCGGCGCGCATAGCAGTATTGTAC
3RC_430        GGATGGCCACCAGCAAGGACGGTTGCACGAGGTGAGGTGCGACTGGGCCAAAACACGCGGCGCGCATAGCAGTATTGTAC
               ********************************************************************************

NbRZ1-Tag      GGGATCCTTGGGCA
1RC_426        GGGATCCTTGGGCA
2RC_426        GGGATCCTTGGGCA
1RC_430        GGGATCCTTGGGCA
3RC_430        GGGATCCTTGGGCA
               **************
```

B

```
NbRZ1-Tag      GAGGGCGTGCTGCGTTTTCGGTGGGGGAGAATGTCACGACCCGATTGTCGTGACCGTAAAGACCAAATGAAAGAGCGGGC
2RC_438        GAGGGCGTGCTGCGTTTTCGGTGGGGGAGAATGTCACGACCCGATTGTCGTGACCGTAAAGACCAAATGAAAGAGCGGGC
3RC_438        GAGGGCGTGCTGCGTTTTCGGTGGGGGAGGATGTCACGACCCGATTGTCGTGACCGTAAAGACCAAATGAAAGAGCGGGC
4RC_438        GAGGGCGTGCTGCGTTTTCGGTGGGGGAGAATGTCACGACCCGATTGTCGTGACCGTAAAGACCAAATGAAAGAGCGGGC
               ***************************** **************************************************

NbRZ1-Tag      CATCTGGATTTTCGTCGTTGGGAAATAAAGGGAACGGCATTAGCGATAAGGCGTTCTCCCTTAGATGCTTCGATCTGGCC
2RC_438        CATCTGGATTTTCGTCGTTGGGAAATAAAGGGAACGGCATTAGCGATAAGGCGTTCTCCCTTAGATGCTTCGATCTGGCC
3RC_438        CATCTGGATTTTCGTCGTTGGGAAATAAAGGGAACGGCATTAGCGATAAGGCGTTCTCCCTTAGATGCTTCGATCTGGCC
4RC_438        CATCTGGATTTTCGTCGTTGGGAAATAAAGGGAACGGCATTAGCGATAAGGCGTTCTCCCTTAGATGCTTCGATCTGGCC
               ********************************************************************************

NbRZ1-Tag      GAGTCAGGCAGGCCGACCCGGTTAAGTTTGGGGAGCTTGTCGCTGGTCATGGACTTGACAATGAAGGGGACCGAAGTAGC
2RC_438        GAGTCAGGCAGGCCGACCCGGTTAAGTTTGGGGAGCTTGTCGCTGGTCATGGACTTGACAATGAAGGGGACCGAAGTAGC
3RC_438        GAGTCAGGCAGGCCGACCCGGTTAAGTTTGGGGAGCTTGTCGCTGGTCATGGACTTGACAATGAAGGGGACCGAAGTAGC
4RC_438        GAGTCAGGCAGGCCGACCCGGTTAAGTTTGGGGAGCTTGTCGCTGGTCATGGACTTGACAATGAAGGGGACCGAAGTAGC
               ********************************************************************************

NbRZ1-Tag      CGAAAGGGACAACTGGTCGGGGGCTAGTGCCGCTCCCCTGATGAGCCCAAGGCAGGGCGAAACCGGTTGGCTTCCGCATG
2RC_438        CGAAAGGGACAACTGGTCGGGGGCTAGTGCCGCTCCCCTGATGACCCCAAGGCAGGGCGAAACCGGTTGGCTTCCGCGTG
3RC_438        CGAAAGGGACAACTGGTCGGGGGCTAGTGCCGCTCCCCTGATGAGCCCAAGGCAGGGCGAAACCGGTTGGCTTCCGCATG
4RC_438        CGAAAGGGACAACTGGTCGGGGGCTAGTGCCGCTCCCCTGATGAGCCCAAGGCAGGGCGAAACCGGTTGGCTTCCGCATG
               ******************************************** ******************************** **

NbRZ1-Tag      TGGTAAGGCTGGTCTGGGGTGGTATGGGACGCCTCAGACGCCGCACGGGCGCTGTGCGCTGCGCCGGACTCTCGTCCCGT
2RC_438        TGGTAAGGCTGGTCTGGGGTGGTATGGGACGCCTCAGACGCCGCACGGGCGCTGTGCGCTGCGCCGGACTCTCGTCCCGT
3RC_438        TGGTAAGGCTGGTCTGGGGTGGTATGGGACGCCTCAGACGCCGCACGGGCGCTGTGCGCTGCGCCGGACTCTCGTCCCGT
4RC_438        TGGTAAGGCTGGTCTGGGGTGGTATGGGACGCCTCAGACGCCGCACGGGCGCTGTGCGCTGCGCCGGACTCTCGTCCCGT
               ********************************************************************************

NbRZ1-Tag      GACAAGTGGTATCAGAGCCGGACGTACTTGCTGTTGTTGCGCGTTTATGGTGGGGTCCTCGGGGGAGGACTGTGCGGGTG
2RC_438        GACAAGTGGTATCAGAGCCGGACGTACTTGCTGTTGTTGCGCGTTTATGGTGGGGTCCTCGGGGGAGGACTGTGCGGGTG
3RC_438        GACAAGTGGTATCAGAGCCGGACGTACTTGCTGTTGTTGCGCGTTTATGGTGGGGTCCTCGGGGGAGGACTGTGCGGGTG
4RC_438        GACAAGTGGTATCAGAGCCGGACGTACTTGCTGTTGTTGCGCGTTTATGGTGGGGTCCTCGGGGGAGGACTGTGCGGGTG
               ********************************************************************************

NbRZ1-Tag      CGCGTTGCCTAGGGATTACAAGGATGACGATGACAAGGGGTGGCT
2RC_438        CGCGTTGCCTAGGGATTACAAGGATGACGATGACAAGGGGTGGCT
3RC_438        CGCGTTGCCTAGGGGTTACAAGGATGACGATGACAAGGGGTGGCT
4RC_438        CGCGTTGCCTAGGGATTACAAGGATGACGATGACAAGGGGTGGCT
               ************** ******************************
```

Figure S9. Sequence analysis of NbRZ1-Tag amplification products obtained for upper leaves of plants agroinfiltrated for expression of NbRZ1-Tag. (**A**) Clones of the (-) strand-specific PCR product obtained with primers specific for the NbRZ1-Tag CR (Figure 4B). (**B**) Clones of the (+) strand-specific PCR product obtained with divergent primers, one of which was complementary to the tag sequence (Figure 4E). Mismatches to the NbRZ1-Tag sequence are shown by gray shading.

Table S1. Retrozyme-containing *N. benthamiana* genomic scaffolds. Genomic scaffolds that contain sequences exhibiting significant (e-value < 1e-10) similarity to the LTR of *N. benthamiana* retrozyme found in the scaffold Niben101Scf01887.

BLASTN 2.6.0+			
Database: Niben.genome.v1.0.1.scaffolds.nrcontigs.fasta<br>56,094 sequences; 2,969,810,994 total letters			
Sequences producing significant alignments	Coverage (%)	Score (Bits)	E Value
Niben101Scf01887	cov=13.0	689	0.0
Niben101Scf04922	cov=14.0	684	0.0
Niben101Scf00825	cov=13.9	684	0.0
Niben101Scf01870	cov=12.7	678	0.0
Niben101Scf19517	cov=14.4	673	0.0
Niben101Scf09914	cov=12.0	553	3e-155
Niben101Scf00321	cov=13.5	531	1e-148
Niben101Scf08455	cov=13.9	507	2e-141
Niben101Scf05269	cov=13.7	505	8e-141
Niben101Scf01476	cov=13.6	505	8e-141
Niben101Scf00305	cov=13.1	503	3e-140
Niben101Scf01313	cov=13.2	494	2e-137
Niben101Scf04505	cov=14.1	490	2e-136
Niben101Scf22660	cov=13.9	484	1e-134
Niben101Scf04528	cov=13.6	484	1e-134
Niben101Scf00961	cov=14.5	479	5e-133
Niben101Scf17535	cov=6.6	473	2e-131
Niben101Scf20037	cov=13.6	457	2e-126
Niben101Scf02207	cov=14.9	457	2e-126
Niben101Scf01485	cov=12.9	451	1e-124
Niben101Scf16010	cov=13.5	446	5e-123
Niben101Scf06068	cov=13.6	438	8e-121
Niben101Scf03766	cov=14.1	438	8e-121
Niben101Scf29314	cov=5.7	435	1e-119
Niben101Scf12469	cov=13.9	433	4e-119
Niben101Scf00530	cov=14.5	433	4e-119
Niben101Scf11350	cov=17.5	429	5e-118
Niben101Scf08394	cov=14.1	429	5e-118
Niben101Scf07841	cov=14.1	425	7e-117
Niben101Scf00110	cov=13.2	418	1e-114
Niben101Scf00751	cov=13.9	416	4e-114
Niben101Ctg13204	cov=15.0	412	5e-113
Niben101Scf18195	cov=15.8	412	5e-113
Niben101Scf08936	cov=13.4	412	5e-113
Niben101Scf12763	cov=12.0	409	7e-112
Niben101Scf00409	cov=12.8	409	7e-112
Niben101Scf02601	cov=13.2	405	9e-111
Niben101Scf31750	cov=13.2	403	3e-110
Niben101Scf21557	cov=13.1	403	3e-110
Niben101Scf11260	cov=12.5	401	1e-109
Niben101Scf00177	cov=13.9	401	1e-109
Niben101Scf06096	cov=13.5	399	4e-109
Niben101Scf05191	cov=13.4	394	2e-107
Niben101Scf13530	cov=14.0	392	7e-107
Niben101Scf03092	cov=13.2	388	9e-106
Niben101Ctg05563	cov=4.0	385	1e-104
Niben101Scf08627	cov=13.8	385	1e-104
Niben101Scf03457	cov=14.1	385	1e-104
Niben101Scf00291	cov=12.0	385	1e-104
Niben101Scf00542	cov=13.8	383	4e-104
Niben101Scf07034	cov=13.4	379	5e-103
Niben101Scf00379	cov=13.6	379	5e-103
Niben101Scf07364	cov=13.2	377	2e-102
Niben101Scf38783	cov=3.4	370	3e-100
Niben101Scf00420	cov=14.0	370	3e-100
Niben101Scf09236	cov=13.1	368	1e-99
Niben101Scf02182	cov=13.8	359	7e-97
Niben101Scf00690	cov=13.4	350	4e-94

Niben101Scf15824	cov=14.8	346	5e-93
Niben101Scf03128	cov=13.2	346	5e-93
Niben101Scf03432	cov=14.6	331	1e-88
Niben101Scf13344	cov=14.1	329	5e-88
Niben101Scf09015	cov=12.8	327	2e-87
Niben101Scf10940	cov=14.3	316	4e-84
Niben101Scf11496	cov=13.5	311	2e-82
Niben101Scf07849	cov=14.7	303	3e-80
Niben101Scf39306	cov=3.0	302	1e-79
Niben101Scf06158	cov=13.6	296	5e-78
Niben101Scf07320	cov=15.5	294	2e-77
Niben101Scf03243	cov=14.7	289	9e-76
Niben101Scf01596	cov=13.7	289	9e-76
Niben101Scf03253	cov=14.5	281	1e-73
Niben101Scf00381	cov=13.6	270	3e-70
Niben101Scf09960	cov=15.0	267	4e-69
Niben101Scf02136	cov=14.0	261	2e-67
Niben101Scf17891	cov=12.9	257	3e-66
Niben101Scf04976	cov=15.3	257	3e-66
Niben101Scf01599	cov=13.6	257	3e-66
Niben101Scf01538	cov=13.7	252	1e-64
Niben101Scf08374	cov=13.8	213	6e-53
Niben101Scf01506	cov=14.8	207	3e-51
Niben101Ctg08192	cov=10.0	204	3e-50
Niben101Scf39521	cov=17.8	204	3e-50
Niben101Scf13540	cov=13.8	204	3e-50
Niben101Scf34433	cov=5.6	189	9e-46
Niben101Scf07466	cov=13.8	187	3e-45
Niben101Scf07288	cov=13.8	182	2e-43
Niben101Scf02606	cov=14.9	180	6e-43
Niben101Scf09087	cov=14.7	176	7e-42
Niben101Scf25048	cov=8.9	174	3e-41
Niben101Ctg01052	cov=5.0	172	9e-41
Niben101Scf11453	cov=13.3	172	9e-41
Niben101Scf01388	cov=14.3	172	9e-41
Niben101Scf12045	cov=13.4	167	4e-39
Niben101Scf07207	cov=13.1	167	4e-39
Niben101Scf05435	cov=13.1	167	4e-39
Niben101Ctg09397	cov=9.0	165	2e-38
Niben101Scf00090	cov=13.3	165	2e-38
Niben101Scf05417	cov=13.4	163	6e-38
Niben101Scf11399	cov=14.5	161	2e-37
Niben101Scf07896	cov=13.5	161	2e-37
Niben101Scf05621	cov=13.7	161	2e-37
Niben101Scf15391	cov=13.9	159	7e-37
Niben101Scf06611	cov=12.4	159	7e-37
Niben101Scf05997	cov=15.7	159	7e-37
Niben101Scf01502	cov=14.7	159	7e-37
Niben101Scf01063	cov=15.0	159	7e-37
Niben101Scf21165	cov=13.9	158	3e-36
Niben101Scf02461	cov=13.1	158	3e-36
Niben101Scf01319	cov=14.7	158	3e-36
Niben101Scf12790	cov=14.9	156	9e-36
Niben101Scf11106	cov=14.4	156	9e-36
Niben101Scf03939	cov=14.3	156	9e-36
Niben101Scf00246	cov=13.4	156	9e-36
Niben101Scf24096	cov=13.1	154	3e-35
Niben101Scf05884	cov=15.0	154	3e-35
Niben101Scf05809	cov=13.7	154	3e-35
Niben101Scf05348	cov=13.4	154	3e-35
Niben101Scf02852	cov=13.7	154	3e-35
Niben101Scf00998	cov=13.8	154	3e-35
Niben101Scf00460	cov=13.6	154	3e-35
Niben101Scf33522	cov=13.7	152	1e-34
Niben101Scf07035	cov=13.3	152	1e-34
Niben101Scf02844	cov=13.3	152	1e-34
Niben101Scf00823	cov=13.6	150	4e-34

Niben101Scf19986	cov=9.6	148	2e-33
Niben101Scf10322	cov=14.3	148	2e-33
Niben101Scf09648	cov=12.3	148	2e-33
Niben101Scf06465	cov=14.1	148	2e-33
Niben101Scf05549	cov=13.2	148	2e-33
Niben101Scf04286	cov=14.0	148	2e-33
Niben101Scf02870	cov=12.3	148	2e-33
Niben101Scf01241	cov=13.7	148	2e-33
Niben101Scf01994	cov=13.7	145	2e-32
Niben101Scf01154	cov=13.1	145	2e-32
Niben101Scf07712	cov=13.1	143	7e-32
Niben101Scf03326	cov=14.0	143	7e-32
Niben101Scf00797	cov=13.4	143	7e-32
Niben101Scf00492	cov=14.2	143	7e-32
Niben101Scf04451	cov=11.7	141	3e-31
Niben101Scf02085	cov=13.9	141	3e-31
Niben101Scf01658	cov=13.9	139	1e-30
Niben101Scf08564	cov=13.7	137	3e-30
Niben101Scf03340	cov=12.8	137	3e-30
Niben101Ctg12727	cov=16.0	134	4e-29
Niben101Scf01947	cov=14.2	132	2e-28
Niben101Scf01534	cov=13.8	132	2e-28
Niben101Ctg15019	cov=16.0	128	2e-27
Niben101Scf11139	cov=12.7	128	2e-27
Niben101Scf02621	cov=14.1	122	1e-25
Niben101Ctg11055	cov=18.0	121	3e-25
Niben101Scf10336	cov=13.4	115	2e-23
Niben101Scf36875	cov=6.1	113	6e-23
Niben101Scf06898	cov=13.0	108	3e-21
Niben101Scf04477	cov=14.8	108	3e-21
Niben101Scf01013	cov=12.9	108	3e-21
Niben101Scf07129	cov=12.0	102	1e-19
Niben101Scf04448	cov=11.8	102	1e-19
Niben101Scf05226	cov=14.2	100	5e-19
Niben101Scf02174	cov=14.2	100	5e-19
Niben101Scf00620	cov=13.9	100	5e-19
Niben101Scf02285	cov=14.0	99.0	2e-18
Niben101Ctg14852	cov=19.0	97.1	6e-18
Niben101Scf29015	cov=13.6	97.1	6e-18
Niben101Scf03898	cov=14.7	97.1	6e-18
Niben101Scf02195	cov=13.1	95.3	2e-17
Niben101Scf00470	cov=14.0	95.3	2e-17
Niben101Scf39198	cov=12.0	93.5	8e-17
Niben101Scf03652	cov=13.4	93.5	8e-17
Niben101Scf07030	cov=13.1	91.6	3e-16
Niben101Scf06698	cov=14.4	91.6	3e-16
Niben101Scf08368	cov=13.5	89.8	1e-15
Niben101Scf04875	cov=13.0	89.8	1e-15
Niben101Scf04556	cov=13.3	89.8	1e-15
Niben101Ctg02061	cov=10.0	87.9	4e-15
Niben101Scf08018	cov=14.8	87.9	4e-15
Niben101Scf16024	cov=13.8	86.1	1e-14
Niben101Scf02738	cov=15.1	86.1	1e-14
Niben101Scf02111	cov=13.3	84.2	5e-14
Niben101Scf14881	cov=13.0	80.5	6e-13
Niben101Scf03700	cov=13.3	80.5	6e-13
Niben101Scf03450	cov=14.6	80.5	6e-13
Niben101Scf03367	cov=15.6	80.5	6e-13
Niben101Scf04049	cov=14.1	78.7	2e-12
Niben101Scf03396	cov=14.2	78.7	2e-12
Niben101Scf03279	cov=12.2	78.7	2e-12
Niben101Scf14323	cov=13.7	76.8	8e-12
Niben101Scf11770	cov=14.0	76.8	8e-12
Niben101Scf05132	cov=13.5	76.8	8e-12
Niben101Scf03607	cov=14.9	76.8	8e-12
Niben101Ctg13528	cov=12.0	73.1	1e-10

Table S2. Contigs assembled from retrozyme-specific HTS reads. Contigs were obtained by constrained assembly of retrozyme-specific reads generated by high-throughput sequencing of *N. benthamiana* leaf RNA.

Name	Number of reads	Sequence
>contig 1	36	GCGGGCCATCTGGATTTTCGTCGTTGGGTACGGGAACGGCATTAGCGATAAGGCGTTGTTCTTCCTT AGATGCTTTGGTCTTGCCGAGTTAGGCAGGCCGACCCGGTTAAGTTTGGGGCGCTTGTCGCTTGTC
>contig 2	2	GGGCCATCTGGATTTTCGTCGTTGGGATAAAAGGGAACGGCATTAGTGATAAGGCGTTCTCCCTTAG ATGCTTCGATCTGGCCGAGTCAAGCAGGCCGACCCGATTAAGTTTGGGGAGC
>contig 3	59	TTAGCGATAAGGCGTTCTCCCTTAGATGCTTCGATCTGGCCGAGTCAGGCAGGCCGACCCGGTTAAG TTTGGGGAGCTTGTCGCTGGTCATGGACTTGACAATGAAGGGGACCGAAGTAGCCGAAAGGGACAAC TGGTCGGGGGCTAGTGCCGCTCCCCTGATGACCCCAAGGCAGGGCGAAACCGGTTGGCTTCCGCGTG TGGTAAGGCTGGTCTGGGGTGGTATGGGACGCCTCAGACGCCGCACGGGCGCTGTGCGCTGCGCCGG ACTCTCGTCCCGTGACAAGTGGTATCAGAGCCGGACGTACTTGCTGTTGTTGCGCGTTTATGGTGGG GTCCTCGGGGGAGGACTGTGCGGGTGCGCGTTGCCTAGGGGGTG
>contig 4	4	GGGGCGCTTGTCGCTGGTCGTGGACTTGACAATGAAGGGCACCGAAATAGGCCTAAGGGATCAACTG GTTGGGGGCTAGTGCCGCTCCCCTGATGAGCCCATGGCAGCG
>contig 5	16	ACCGAAGCAATCCTAAGGGACAACTGGTTGGGGGCTAGTGCCGCTCCCCTGATGAGCCCAAGGCAGG GCGAAACAGGTTGGCTTCCGCATGTGGTAAGGCTGGTCTGGGGTGGTATGGGACGCCTCAGACGCCG CACGG
>contig 6	2	CCGAAAGGGACAACTGGTCGGGGGCTAGTGCCGCTCCCCTGATGAGCCCAAGGCAGGGCGAAACCGG TTGGCTTCCGCATGTGGTAAGGCTGGTCTGGGGTGGTATGGGACGCCTCA
>contig 7	8	GTTGGGGGCTAGTGCAGCTCCCCTGATGAGCCCAAGGCAGGGCGAAACCGGTTGGCTTCCGCATGTG GTAAGGCTGGTCTAGGGTGGTATGGGACGCCTCAGA
>contig 8	4	GTCTAGGGTGGTATGGGACGCCTCAGACGCCGCACGGGCGCTGTGCGCTGCGCCGGACTCTCAGCCT GTGACAAGTGGTATCAGAGCGGGACGTACTTGCTGCTGTT
>contig 9	38	CCGGACTCTCAGCCTGTGACAAGTGGTATCAGAGCGGGACGTACTTGCTGCTGTTGTGCGTTATGGT GGGATTCTCAGGGGAGGACTGTGCGGGTGCGTTGCCTGGGGGGGCTCGCGCCGACGACTGCCCCATC CTGCAATGGTCTGCAGGGGCAAGACCGGGATGGCTACCAGCAAGGCCGGTTGCACGAGG
>contig 10	14	CGTGCTGCGTTTTGGGTGGGGGAGAATGTCACGACCCGATTGTCGTGACCGTTAAAGATCAAATGAA AGAGCGGGCCATCTGGCAATTCGTCGTTGGGTACGGGAACGGCATTAGCGATA
>contig 11	2	GTGGGGGAGAATGTCACGACCCGATTTTCGTGACCGTTAAGGTCAAATGAAAGAGCGGGCCATCTGG AAATTCGTCGTTGGGATACGGGAACGGCATTAGCG
>contig 12	27	GTTTGGGGTGCTTGTCGCTGGTCATGGACTTGACAATGAAGGGGACCGAAGTAGGCCAAAGGGATCA ACTGGTCGGGGGCTAGTGCCGCTCCCCTGATGAGCCCAAGGCAGGGCGAAAACGGTTGGCTTCCGCG TGTGGAAAGG
>contig 13	5	GGGCGCTTGTCGCAGGTCATAGACTTGACAATGAAGGGGACCGAAGTAGGCAAAAGGGATCAACTGG TCGGGGGCTAGTGCTGCTCCCCTGATGAGCCCAA
>contig 14	27	GGGGGTGCTTGTCGCTGGTCATGGACTTGACAATGAAGGGGACCGAAGTAGGCCAAAGGGATCAACT GGTCGGGGGCTAGTGCCGCTCCCCTGATGAGCCCAAGGCAGGGCGAAAACGGTTGGCTTCCGCGTGT GGAAAGG
>contig 15	2	GTTCATACTCAATGTGATGTGTCACGACCCGATTGTCGTGACCGTTAAAGATCAAATGAAAGAGCGG GCCATCTGGATTTTCGTCGTTGGGTACGGGAACGGCATTA
>contig 16	2	GGGCGCTTGTCGCTGGTCATGGACTTGACAATGAAGGGGACCGAAGTAGGCAAAAGGGATCAACTGG TCGGGGGCTAGTGCCGCTCCCCTGATGAGCCCA
>contig 17	2	GACCGGGATGGCTACCAGCAAGGCCGGTTGCACGAGGTGAGGTGCGCTTGGGCAAAACACGCGGCGC GTATAGCAGTATTGTACGTGATCCTTGGGCAGAGTCCTTCTCCCCAACAGCACGA
>contig 18	2	GTGGGGGAGAATGTCACGACCCGATTGTCGTGACCGTGAAGATCAAAAGAAAGAGCGGGCCATCTGG ATTTTCGTCGTTGGGATAAAAGGGAACGGCATTAG
>contig 19	5	GGGGGGAGAATGTCACGACCCGATTGTCGTGACCGTTAAAGATCAAATGAAAGAGCGGGCCATCTGG CAATTCGTCGTTGGGTACGGGAACGGCATTAGCGATA
>contig 20	33	GGGGGTGCTTGTCGCTGGTCATGGACTTGACAATGAAGGGGACCGAAGTAGGCCTAAGGGATCAACT GGTCGGGGGCTAGTGCCGCTCCCCTGATGAGCCCAAGGCAGAGCGAAACCGGTTGGCTTCCGCGTGT GGTAA
>contig 21	4	AGGGGACCGAAGTAGGCCTAAGGGATCAACTGGTCGGGGGCTAGTGCCGCTCCCCTGATGAGCCCAC GGCAGGGCGAAACTGGTTGGCTTCCGCGTGTGGTAAGGCTTGTCTCG
>contig 22	2	GGCCAAAACACGCGGCGCGCATAGTAGTATTGTACGGGATCCTTGGGTAGAGTCCTTCTCCCCAACA GCACGAGGGCGTGCTGCGTTTTGGGTGGGGGAGA
>contig 23	3	GCGCTGTGTGCTGCGCCGGACTCTCAGCCCGTGACAAGTGGTATCAGAGCGGGACATACTTGCTGCT GTTGCGATGATTGGGTCTTAGAGGAGAGGACTG
>contig 24	2	GGCGCGTATAGCAGTATTGTACGTGATCCTTGGGCAGAGTCCTTCTCCCCAACAGCACGAGGACGTG CTGCGTTTTGGGTGGGGGAGAATGTCACGACCCGATTGTCGTGACCGATAA
>contig 25	13	GGCGCGTATAGCAGTATTGTACGTGATCCTTGGGCAGAGTCCTTCTCCCCAACAGCACGAGGACGTG CTGCGTTTTGGGTGGGGGAGAATGTCACGACCCGATTGTCGTGACCGTTAAAGATCAAATGAAAG

>contig 26	11	CGGGCAGAGTCCTTCTCCCCAACAGCACGAGGACGTGCTGCGTTTTGGGTGGGGGAGAATGTCACGACCCGATTGTCGTGACCGTTAAAGATCAAATGAAAG
>contig 27	11	AGGGCAGAGTCCTTCTCCCCAACAGCACGAGGACGTGCTGCGTTTTGGGTGGGGGAGAATGTCACGACCCGATTGTCGTGACCGTTAAAGATCAAATGAAAG
>contig 28	26	GCGGGCCATCTGGCAATTCGTCGTTGGGTACGGGAACGGCATTAGCGATAAGGCGTTGTTCTTCCTTAGATGCTTTGGTCTTGCCGAGTTAGGCAGGCCGACCCGGTTAAGTTTGGGGCGCTTGTCGCTTGTC
>contig 29	7	GCGGGCCATCTGGCAATTCGTCGTTGGGTACGGGAACGGCATTAGCGATAAGGCGTTGTTCTTCCTTAGATGCTTTGGTCTTGCCGAGTTAGGCAGGCCGACCCGGTGAAGTTTGGGGC
>contig 30	3	GCCGCACGGGCGCTGTGTGCTGCGCCGGACTCTCAGCCCGTAACATTACGCTGTTGAATACAATAGGATAAGAAGCACAAAATTAAACAATATTACTCTGTCTTCTTGA
>contig 31	4	CCCTGTAGTCCAAAGGAAAGAGCGGGCCATCTGGAACATTGCCGTTGGGTTATGGGGACGACATTAGCATTAAGGCGTTGTTCTCCCTTAGATGCTTCGATCTGGCCGAGTCAGGCAGGCCGACCCGGTT
>contig 32	19	GTCCGAAGGAAAGAGCGGGCCATCTGGAACATTGTCGTTGGGTTATGGGGACGATATTAGCATTAAGGCGTTGTTCTCCCTTAGATGCTTCGATCTGGCCGAGTCAGGTAGGCCGACCCGGTTAAGTTTGGGGTGCTTGTCGCTGGTC
>contig 33	11	CGGGCCATATGGAACTTCGTCGGTTGGGTTATAGGGTCGACATTAGCATTAAGGCGTTGTTCTCCCTTAGATGCTTCGATCTGGCCGAGTCAGGCAGGCCGACCCGGTTAAGTTTGGGGTGCTTGTCGCTGGTC
>contig 34	2	GGGCCATCTGGAACTTCGTCGTTGGGTTATGGGGACGATAATAGCATTAGGGCGTTGTTCTCCCTTAGATGCTTCGATCTGGCCGAGTCAGGCTGGACGACCCGGTT
>contig 35	8	GTCATATGGAACTTCGTCGGTTGGGTTATAGGGTCGACATTAGCATTAAGGCGTTGTTCTCCCTTAGATGCTTCGATCTGGCCGAGTCAGGCAGGCCGACCCGGTTAAGTTTGGGGTGCTTGTCGCTGGTC
>contig 36	2	GCTCCCCTGATGAGCCCAAGGCAGAGCGAAACCGGTTGGCTTCCGCGTGTGGTAAGGCTTGTCTCGAGGTAGTGGGCGGCCTCGGGACGCCGCACGGGCGCTGT
>contig 37	21	CCGGACTCTCAGCCTGTGACAAGTGGTATCAGAGCGGGACGTACTTGCTGCTGTTGTGCGTTATGGTGGGATTCTCAGGGGAGGACTGTGCGGGTGCGTTGCCTGGGGGGGCTCGCGCCGACGACTGCCCCATCCTGCAATGGTCTGCAGGGGCAAGACCGGGATGGCTACCAGCAAGACCGGTTGC
>contig 38	2	GTCGGCTATTTTCGTGAGTTGGTGCTTGGTTGCCGCGCGGGCTGTTTTAGAGTGACATAAAACGAGCCCGTGACAATTGGTATCAGAGCGGGCCAGGTTTCATGACTAGGTCGAGATGGAC
>contig 39	2	CCCGTGAAAAGTGGTATCAGAGAGGGACGTACTTGCTGCTGCGCGTTATGGTGGGGTCCTCGAGGGGAGGACTGTGCGGGTGCGTTGCTAGGCGGGGGGC
>contig 40	2	GCTGCTGCGCGATATGGTGGGGTCCTCGGGGGAGGACTGTGCGGGTGCGTTGCCTAGGCGGGGGGCTCGCGCCGACGACTGCCCCATCCTGCTAAGGTCTGCAAGG
>contig 41	4	GCGGGTGCGTTGCCTAGGCGGGGGGCTCGCGCCGACGACTGCCCCATCCTGCTAAGGTCTGCAAGGGCAAGACCGGGATGGCCACCAGCAAGGCCGGTTG
>contig 42	5	TGGTGCGTTGCCTGGGGGGGCTCGCGCCGACGACTGCCCCATCCTGCAATGGTCTGCAGGGGCAAGACCGGGATGGCTACCAGCAAGGCCGGTTGCACGAGG
>contig 43	2	GCCGGGATGGCCACCAGCAAGGCCGGTTGCACAAAGTGAGGTGCGACTGAGCGAAGATACGCGGCGCGTTAAGCAGTATTGTGCGGGTTCCTGGGCGGAG
>contig 44	3	GCGACTGAGCGAAGAGACGCGGTGCGTTAAGCAGTATTATGCGGGACCCTGGGCGGAGTCCTTCTCCCCAACAGCACGAGGACGTGCTGGGTTTTGAGTGGGGGAGAATGTCACGA
>contig 45	2	GGCGCTTTAGCAGTATTGTGCGGGATCCTGGGCGGAGTCCTTCTCCCCAACAGCACGAGGACGTGCTGGGTTTTGGGTGGGGGAGAATGTCACGGCCTGT
>contig 46	5	GTATTGTGCGGGTTCCTGGGCGGAGTCCTTCTCCCCAACAGCACGAGGACGTGCTGGGTTTTGGGTGGGGGAGAATGTCACGACCCGTTTGTCGTGACCGTGTAGATCAAAAGAAAGAGCGGGC
>contig 47	5	GGTGCGGGTTCCTGGGCGGAGTCCTTCTCCCCAACAGCACGAGGACGTGCTGGGTTTTGGGTGGGGGAGAATGTCACGACCCGTTTGTCGTGACCGTGTAGATCAAAAGAAAGAGCGGGC
>contig 48	3	GGACGTGCTGGGTTTTGGGTGGGGGAGAATGTCACGACCCGTTTGTCGTGACCATATAGTCCAAAGGAAAGAGCGGGCCATCTGGAACTTCGTCGTTGGGTCATGGGGACGACATTAG
>contig 49	4	GCTGGGTTTTGGGTGGGGGAGAATGTCACGACCCGTTTGTCGTGACCGTGTAGTCCGAAGGAAAGAGCGGGCCATCTGGAACATTGTCGTTGGGTTATGGGGACGATATTAGCATTA
>contig 50	3	GTTTTGGGTGGGGGAGAATGTCACGACCCGTTTGTCGTGACCGTGTAGTCCAAAGGAAAGAGCGGGCTATCTGGAACTTCGTCGTTGACTCATGGGGACGACATTAGCAT
>contig 51	4	ACCCGGTTAAGTTTGGGGTGCTTGTCGCTGGTCATGGACTTAACAATGAAGGGGACCGAAGTAGGCCTAAGGGATCAACTGGTCGGGGGCTAGTGCCGCT
>contig 52	3	GGGGTGCTTGTTGCTGGTCATGGACTTGACAATGATGGGGACCGAAGTAGGCCTAAGGGATCAACTGGTCGGGGGCTAGTGCCGCTCCCCTGATGAACCCA
>contig 53	2	GGTTGGCTTCCGCATGTGGTAAGGCTGGTCTAGGGTGGTATGGGACGCCTCAGACGCCGCACGGGCGCTGTGCGCTGCGCCGGACTCTCAGCCTGTGACAA
>contig 54	34	GTTGGCTTCCGCATGTGGTAAGGCTGGTCTGGGGTGGTATGGGACGCCTCAGACGCCGCACGGGCGCTGTGCGCTGCGCCGGACTCTCAGCCTGTGACAGATGTCATGGATGACATTATTAGGACAATTTCCCTTC
>contig 55	25	GGTTTCCGCATGTGGTAAGGCTGGTCTGGGGTGGTATGGGACGCCTCAGACGCCGCACGGGCGCTGTGCGCTGCGCCGGACTCTCAGCCTGTGACAGATGTCATGGATGACATTATTAGGACAATTTCCCTTC
>contig 56	24	GTCCGCATGTGGTAAGGCTGGTCTGGGGTGGTATGGGACGCCTCAGACGCCGCACGGGCGCTGTGCGCTGCGCCGGACTCTCAGCCTGTGACAGATGTCATGGATGACATTATTAGGACAATTTCCCTTC
>contig 57	2	GGGGTGGTATGGGACGCCTCAGACGCCGCACGGGCGCTGTGCGCTGCGCCGGACTCTCGGCCCGTGACATTATAATGAGACAAGTACAATAGCCTTAAAAGAC
>contig 58	9	CCGTCGTTGGGTACGGGAACGGCATTAGCGATAAGGCGTTGTTCTTCCTTAGATGCTTTGGTCTTGCCGAGTTAGGCAGGCCGACCCGGTTAAGTTTGGGGCGCTTGTCGCTTGTC

>contig 59	4	AGGCGTTGTTCTTCCTTAGATGCTTTGGTCTTGCCGAGTTAGGCAGGCCGACCCGGTTAAGTTTGGGGAGCTTGTCGCTTGTCTTGGACATGACTATGAAGTGGACCGAAGCAATCCTAAGG
>contig 60	20	AGTTAGGCAGGCCGACCCGGTTAAGTTTGGGGCGCTTGTCGCTTGTCTTGGACATGACTATGAAGTTGACCGAAGCAATCCTAAGGGACAACTGGTTGGGGGCTAGTGCCGCTCCCCTGATGAGCCCAAGGCAGGGC
>contig 61	24	AGTTAGGCAGGCCGACCCGGTTAAGTTTGGGGCGCTTGTCGCTTGTCTTGGACATGACTATGAAGTTGACCGAAGCAATCCTAAGGGACAACTGGTTGGGGGCTAGTGCAGCTCCCCTGATGAGCCCAAGGCA
>contig 62	2	CCGGTTAAGTTTGGGGAGCTTGTCGCTTGTCTTGGACATGACTATGAAGTGGACCGAAGCAATCCTAAGGGACAACTGGTCGGGGGCTAGTGCCGCTCCC
>contig 63	2	GGGGCGCTTGTCGCTTGTCTTGGACATGACTATGAAGTTGACCGAAGCAATCCTAAGGAACAACTGGTTGGGGGCTAGTGCCGCTCCCCTGATGAGCCCA
>contig 64	2	GGGGCGCTTGTCGCTTGTCTTGGACATGACTATGAAGTTGACCGAAGCAATCCTAAGGGACAACTGGTCGGGGGCTAGTGTCGCTCCCCTGACGAGCCCA
>contig 65	56	GCAGCGAAAGTAATGCGTCGGGTCGCCGCATCATCGGGTGGGGGAGAGTTGTCACGGCCTCAAAGTAGGGACAAAGCGTGCGTGCGGATTGGCATCAAACTGCCTTGGGGAGGGGAGGCGCCTCTAGCCATAAAGGCGCTCTGCCCTAGTTGCGTATTGGCTTGGCCGAGTTAGGCATGCCGACCCGATTAAGTTTGGGGAG
>contig 66	12	GCGGAAACTTTTGAAGGGAGTATGGAGTGAAAAGGTCGAAATGAAGACTGAAGTGTCACGGCCTCAGAGTAGGGACAAAGCGTGCGTGCCGATTGGCATCAAACTGCCTTGGGGAGGGGAGGCGCCTCTAGCCATAAAGGCGCTCTGCCCGAGT
>contig 67	2	CGGGTGGGGGAGAGTTGTCACGGCCTCAAAGTAGGGACAAAGCGTGCGTGCGGATTGGCATCAAACTGCCTTGGGGAGGGGAGGCGCCTCTAGCCATAATGGCGCT
>contig 68	37	CGGGGGAGAGTTGTCACGGCCTCAAAGTAGGGACAAAGCGTGCGTGCGGATTGGCATCAAACTGCCTTGGGGAGGGGAGGCGCCTCTAGCCATAAAGGCGCTCTGCCCTAGTTGCGTATTGGCTTGGCCGAGTTAGGCATGCCGACCCGATTAAGTTTGGGGAG
>contig 69	17	GTCACGGCCTCAGAGTAGGGACAAAGCGTGCGTGCCGATTGGCATCAAACTGCCTTGGGGAGGGGAGGCGCCTCTAGCCATAAAGGCGCTCTGCCCTAGTTGCGTATTGGCTTGGCCGAGTTAGGCATGCCGACCCGATTAAGTTTGGGGAG
>contig 70	16	CCACGGCCTCAGAGTAGGGACAAAGCGTGCGTGCCGATTGGCATCAAACTGCCTTGGGGAGGGGAGGCGCCTCTAGCCATAAAGGCGCTCTGCCCTAGTTGCGTATTGGCTTGGCCGAGTTAGGCATGCCGACCCGATTAAGTTTGGGGAG
>contig 71	11	GGACGGCGTTGTATTGGCGTCGAAGCGTGCGTGCTGACTGGCATCAAACTGCCTTGGGGAGGGGAGGCGTATCTAGCAATAAGACGCTCTGCCCTAGTTGCGTATTGGCTTGGCCGAGTTAGGCAT
>contig 72	10	CGGCCTCAGAGTAGTGACTATGGACGGCGTTGTATTGGCGTCGAAGCGTGCGTGCTGACTGGCAATCAAACTGCCTTGGGGAGGGGAGGCGTCTCTAGCCATAAGGCGCTCTGCCCTAGTTGCGTATTGGCTTGGC
>contig 73	6	GTGCTTGCGGATTGGCATCAAACTGCCTTGGGGAGGGGAGGCGCCTCTAGCCATAAAGGCGCTCTGCCCTAGTTGCGTATTGGCTTGGCCGAGTTAGGCATGCCGACCCGATTAAGTTTGGGGAG
>contig 74	8	CTCTGCCCTAGTTGCGTATTGGCTTGGCCGAGTTAGGCATGCCGACCCGATTAAGTTTGGGGAGCATGTCGCTTGTCTTGAACATGACTATGAAGGGAGCCGAAGCATACGGGGGGGCACAGCTGG
>contig 75	26	CTCAGAGAAGGGGCAAAGTAAAGCGTGCGGATTGGCATCAAACTGCCTTGGAGAGGGGAGGCGTCTCTAGCCATTAAGGCGCTCTGCCCTAGTTGCGTATTGGCTTGGCCGAGTTAGGCATGCCGACCCGATTAAGTTTGGGGAGCATGTCGCTTGTCTTGGACATGACTATGAAGGGAGCCGAAGCATACGGGGGGGCACAGCTGG
>contig 76	2	GTATTGGCTTGGCCGAGTTAAGCATGCCGACCCGATTAAGTTTTGGGAGCATGTCGCTTGTCTTGGACAGGACTATGAAGGGAGCTGAAGCATACGGGGG
>contig 77	2	ATTGGCTTGGCCGAGTTAGGCATGCCGACTCGATTAAGTTTGGGGAGCATGTCGCTTGTCTTGGACATGACTATGAAGGGAGCTGAAGCATACGGGGGGC
>contig 78	4	GTTTGGGGAGCATGTCGCTTGTCTTGAACATGACTATGAAGGGAGCCGAAGCATACGGGGGGGCACAGCTGGTCGGGAGCTATTACCGCTTCCCTGATGAGCCC
>contig 79	2	CGCTTGTCTTGGACATGACTATGAAGGGAGCCGAAGCATACGGGGGGCTCAGCTGGTCGGGAGCTATTGCCGCTTCCCTGATGAGCCCATGGCAGGGCGA
>contig 80	7	TGAAGGGAGCCGAAGCATACGGGGGGGCACAGCTGGTCGGGAGCTATTGCCGCTTCCCTGATGAGCCCATGGCAGGGCGAAACCAGTCTGCGCGCTAGGTCGGACGGGCGTGTCCGGGGCGGT
>contig 81	10	GGGAGCTGAAGCATACGGGGGGCACAGTTGGTCGGGAGCTATTGCCGCTTCCCTGATGAGCCCATGGCAGGGCGAACCAGTCTGTACGCTATTCGGACGGGCGAGTCCGGGGTGGTATGGGACGCCTCGGGCGCCGCGGG
>contig 82	6	GGAGCTGAAGCATACGGGGGCACAACTGGTCGGGAGCTATTGCCGCTTCCGTAATGAGCCCATGGCAGGGCGAAACCAGTCTGTACGCTATTCGGACGGGCGAGTTCGGGGTGGTATGGGACGCCTCG
>contig 83	15	AGCCGAAGCATACGGGGGGGCACAGCTGGTCGGGAGCTATTACCGCTTCCCTGATGAGCCCATGGCAGGGCGAAACCAGTCTGCGCGCTAGGTCGGACGGGCGTGTCCGGGGCGTTTATGGGACGCCTCGGGCGCGAAGCGCCGCGGTCCGTCACAGAAGGATCAGGAAATATAAGCACTCAAAAGTTCAAATGGATTATAAAGAATT
>contig 84	5	AGCTGAAGAATACGGGGGGCACAGTTGGTCGGGAGCTATTGCCGCTTCCCTGATGAGCCCATGGCAGGGCGAACCAGTCTGTACGCTATTCGGACGGGCGAGTCCGGGGTGGTATGGGACGCCTCGGGCGCCGCGGG
>contig 85	6	GGCCGAAGCATACGGGGGGGCACAGCTGGTCGGGAGCTATTGCCGCTTCCCTGATGAGCCCATGGCAGGGCGAAACCAGTCTGCGCGCTAGGTCGGACGGGCGTGTCCGGGGCGGT
>contig 86	2	GCATACGGGGGCACAGCTGGTTGGGAGCTATTGCCGCTTCCCTGATGAGCCCATGGCAGGGCGAAAC

		CTGTCTGTACGCTATTTGGACGGGCGAGTCTGGGGTGGTAT
>contig 87	13	CGGGGGGGGCACAGCTGGTCGGGAGCTATTACCGCTTCCCTGATGAGCCCATGGCAGGGCGAAACCA GTCTGCGCGCTAGGTCGGACGGGCGTGTCCGGGGCGTTTATGGGACGCCTCGGGCGCGAAGCGCCGC GGTCCGTCACAGAAGGATCAGGAAATATAAGCACTCAAAAGTTCAAATGGATTATAAAGAATT
>contig 88	10	ACCCAGTCTGCGCGCTAGGTCGGACGGGCGTGTCCGGGGCGTTTATGGGACGCCTCGGGCGCGAAGC GCCGCGGTCCGTCACAGAAGGATCAGGAAATATAAGCACTCAAAAGTTCAAATGGATTATAAAGAAT T
>contig 89	25	CCAGTCTGCGCGCTAGGTCGGACGGGCGTGTCCGGGGCGGTTATGGGACGCCTCGGGCGCGAAGCGC CGCGGTCCGTCACATATGGTATCAGAGCAAACATGCTCTTCAACTTCTCTTGCGAGGTGTCAGCTCA TG
>contig 90	22	CGTCGGACGGGCGTGTCCGGGGCGGTTATGGGACGCCTCGGGCGCGAAGCGCCGCGGTCCGTCACAT ATGGTATCAGAGCAAACATGCTCTTCAACTTCTCTTGCGAGGTGTCAGCTCATG
>contig 91	2	GTCAGCTCATGAATGCACAAGCTCGGGTGAATGCTACAAGTGCAAAGATGGGGGCGCTGAGTGACAG AGTGGAGCATGACGGTCCTTGGGCAAACATACTACTTCTCCGCCAAAGTAAAGC
>contig 92	2	CTTGGGTGAATGCTACAAGTGCAAAGATGGGGGCGCTGAGTGACAGAGTGGAGCATGACGGTCCTTG GGCAAACATACTACTTCTCCGCCAAAGTAAAGC
>contig 93	5	GGATGCTATAAGTGCAAAGATGGGGATGCTGAGTGACAGAGTGGAGCATGACGATTTTGGGCAAAGA GACTACTTCTCCGCCAAAGTAAAGCGGCTTGAGCAATGAAATTGTTGTGAACTCTAAATGAAACCAC GAAGTGGACACACCGACGAAGTGCG
>contig 94	43	TGGGGGCGCTGAGTGACAGAGTGGAGCATGACGGTCCTTGGGCAAACATACTACTTCTCCGCCAAAG TAAAGCGGGCTTGAGCAATGAAATTATTGTGAACTCTAGATGAAACCACGAAGTGGACGCACCGACG AAGTG
>contig 95	32	TGGGGCGCTGAGTGACAGAGTGGAGCATGACGGTCCTTGGGCAAACATACTACTTCTCCGCCAAAGT AAAGCGGGCTTGAGCAATGAAATTATTGTGAACTCTAGATGAAACCACGAAGTGGACGCACCGACGA AGTG
>contig 96	22	CTGGGCGCTGAGTGACAGAGTGGAGCATGACGGTCCTTGGGCAAACATACTACTTCTCCGCCAAAGT AAAGCGGGCTTGAGCAATGAAATTATTGTGAACTCTAGATGAAACCACGAAGTGGACGCACCGACGA AGTG
>contig 97	11	GCTCCTTGGGCAAACATACTACTTCTCCGCCAAAGTAAAGCGGGCTTGAGCAATGAAATTATTGTGA ACTCTAGATGAAACCACGAAGTGGACGCACCGACGAAGTG
>contig 98	17	AGCGGGCTTGAGCAATGAAATTATTGTGAACTCTAGATGAAACCACGAAGTGGACGCACCGACGAAG TGTGATGCCGTCTCCATGATTACGTGCCTGAGTCCCAAAGTTGG
>contig 99	3	GCACCGACGAAGTGTGATGCCGTCTCCATGATTACGTGCCTGAGTCCCAAAGTTGGCAGCACGAAGA GGTGTGGTGCAAGCATGAATGAGGGTGCAAGTTCCGTGTGGAACACGAAAGGAGTT
>contig 100	13	GGCAGCACGAAGAGGTGTGGTGCAAGCATGAATGAGGGTGCAAGTTCCGTGTGGAACACGAAAGGAG TTGCAGCGAAAGTAATGCGTCGGGTCGCCGCATCATCGGGTGGGGGAGAGTTGTCACGGCCTCAAAG T
>contig 101	13	CGTGGTGCAAGCATGAATGAGGGTGCAAGTTCCGTGTGGAACACGAAAGGAGTTGCAGCGAAAGTAA TGCGTCGGGTCGCCGCATCATCGGGTGGGGGAGAGTTGTCACGGCCTCAAAGT
>contig 102	7	GGGGTGCAAGCATGAATGAGGGTGCAAGTTCCGTGTGGAACACGAAAGGAGTTGCAGCGAAAGTAAT GCGTCGGGTCGCCGCATCATCGGGTGGGGGAGAGTTGTCACGGCCTCAAAGT
>contig 103	7	GTTAGCATGAATTAGGGTGCAAGATCCGTGCGGAAACGAAAGGAGTTGCAGCGAAAGTAATGCGTCG GGTCGCCGCATCATCGGGTGGGGGAGAGTTGTCACGGCCTCAGAGTAGTGACTATGGACGGCGTTGT ATTGGCGTCGAAGCGTGCGTGCTGACTGGCATCAAAC
>contig 104	7	GCATGAATTAGGGTGCAAGTTCCGTGTGGAAACGAAAGGAGTTGCAGCGAAAGTAATGCGTCGGGTC GCCGCATCATCGGGTGGGGGAGAGTTGTCACGGCCTCAGAGTAGTGACTATGGACGGCGTTGTATTG GCGTCGAAGCGTGCGTGCTGACTGGCATCAAAC
>contig 105	2	CATGAATTAGGGTGGAAGTTCCGTGTGGAAACAAAAGGGGTTGCAGCGAAAGTAATGCGTCGGGTCG CCGCATCATCGGGTGGGGAAGAGTTGTCACGGCCTCAGAGAAGT
>contig 106	18	CTCAGAGAAGGGGCAAAGTAAAGCGTGCGGATTGGCATCAAACTGCCTTGGAGAGGGGAGGCGTCTC TAGCCATTAAGGCGCTCTGCCCTAGTTGCGTATTGGCTTGGCCGAGTTAGGCATGCCGACCCGATTA AGTTTGGGGAGCATGTCGCTTGTCTTGGACATGACTATGAAGGGAGCCGAAGCATACGGGGGGC
>contig 107	3	GGGGAGGGGGAGGCGTCTCTAGCCATAAGGCGCTCTGCCCTAGTTGCGTATTGGCTTGGCCGAGTTAG GCATGCCTACCCGATTAAGTTTGGGGAGCATGTC
>contig 108	5	GCTTGGCCGAGTTAGGCATGCCGACCCGATTAAGTTTGGGGAGCTTGTCGCTTGTCTTGGACATGAC TATGAAGGGAGCTGAAGCATACGGGGGCACAGCTGGT
>contig 109	2	GGGGAGCAAGTCGCTTGTCTTGGACATGACTATGAAGGGAGCTGAAGCATACGGGGGCACAACTGGT CGGGAGCTATTGCCGCTTCCGTAATGAGCCCATG
>contig 110	2	GGCAGGACGGGCGTGTCCGGGGCGGTATGGGACGCCTCGGGCGCGGAGAGCCGCGGTCCGTGTCATT TGGTATCAGAGCAAACATGCTCTTCAACTTCTGTGGCGAG
>contig 111	3	GGCGTGTCCGGGGCGGTATGGGACGCCTCGGGCGCGAAGCGCCGCGGTCCGTGTCACTTGGCTCAAG CTTATTTTAATCAATTATCAAGAAGTAGAAAGACACAGCGTAAT
>contig 112	2	GTCCAGGGAGGTATGGGACCCTCGGGCGCGGAGCGCCGCGGTCCGTGTCATTATGCTAGGGAATGTT TCAAAGCTGGACTTGAGCACAGTGGTGGAAAGTATG
>contig 113	2	ACCGGGGCGGTATGGGACGCCTCGGGCGCGAAGCGCCGCGGTCCGTGTCACTTGGCTCAAGCTTATT TTAATCAATTATCAAGAAGTAGAAAGACACAGCGTAAT
>contig 114	2	GGCGGATTGTGACTAAGGCCGCACGGAAGATAGCTAGGGAGTTATCCGGTCCGTGACAGTTGGTATC AGAGCAAACATGCTCTTCAACTTCTGTGGCGAGGTGCC
>contig 115	3	CCGTGTCATTTGGTACCAGAGCAAACATGCTCTTCAACTTCTGTGGCGAGGTGTCAAGCTCATGAAT

		GCACAAAGCTAGCGGAAGGCGTAAGTGCAAAGA
>contig 116	3	TGGCGAGGTGTCAAGCTCATGAATGCACAAAGCTAGCGGAAGGCGTAAGTGCAAAGATGGGGGCGCTGAGTTACAGAGTGGGGCATGACGGTCCTTGGGGC
>contig 117	4	GTGCAAAGATGGGGGCGCTGAGTTACAGAGTGGGGCATGACGGTCCTTGGGGAAAACATACTACTTCTCCGCCAAAGTAAAGCGGGCTTGAGCAAATGAAATTGTTGCGAACTCTAGATGAAACCACGAAGTGG
>contig 118	50	TTGCACTTCAACTTCTGTGGCGAGGTGTCAAGCTCATGAATGCACAAAGCTAGCGGAAGGCACAAGTGCAAAGATGGGGGCGCTGAGTTACAGAGTGGGGCATGACGGTCCTTGGGGCAAACATACTACTTCTCCGCCAAAGTAAAGTGGGCTTGAGCAAATGAAATTGTTGCGAACTCTAAAAGAAACCACGAAG
>contig 119	6	AGGGCGCTGAGTTACAGAGTGGGGCATAACGGTCCTTGGGGAAAACATACTACTTCTCCGCCAAAATAAAGCGGGCTTGAGCAAATGAAATTGTTGCGAACTCTAGATGAAACCACGAAGTG
>contig 120	51	GGCTTGAGCAAATGAAATTGTTGCGAACTCTAGATGAAACCACGAAGTGGACGCGCCGACGAAGTGTGAAGCTCTCCATGGTTTCGTGCCTAAGTCCGAGGTAGGCAGCACGAGGAGGTATTGCTAGCATGATGGGTGCAATGTTCCGAGTGGAAGACGAAAGGAGTAGCAGCGAAAGTAATGCGTCGGGTCGCCGCATCAGCCAGGTGGGGGAGGGTTGTCACGTCCTCAGAGAAGGGGC
>contig 121	2	GGCTTGAGCAAATGAAATTGTTGCGAACTCTAAAAGAAACCACGAAGTGGACGCGCCGACGAAGTGTGAAGCTCTCCATCGTTTCGTGCCTAAGTCCGAG
>contig 122	2	GTCCGAGGTAGGCAGCACGAGGAGGTATCGCTTGCATGATGGGTGCAATGTTCCGAGTGGAAGACGAAAGGAGTAGCAGCGAAAGTAATGCGTCGGGTCG
>contig 123	52	TTGTTGCGAACTCTAGATGAAACCACGAAGTGGACGCGCCGACGAAGTGTGAAGCTCTCCATGGTTTCGTGCCTAAGTCCGAGGTAGGCAGCACGAGGAGGTATTGCTAGCATGATGGGTGCAATGTTCCGAGTGGAAGACGAAAGGAGTAGCAGCGAAAGTAATGCGTCGGGTCGCCGCATCAGCCAGGTGGGGGAGGGTTGTCACGTCCTCAGAGAAGAGGCAAAGTAAAGCGTGCGGATTGGCATCAAACTGCCAGATCGGA
>contig 124	10	GGTGGGGGAGGGTTGTCACGTCCTCAGAGAAGAGGCAAAGTAAAGCGTGCGGATTGGCATCAAACTGCCTTGGAGAGGGGAGGCGTCTCTAGCCATTAAGGCGCT
>contig 125	11	CGGGGGAGGGTTGTCACGTCCTCAGAGAAGAGGCAAAGTAAAGCGTGCGGATTGGCATCAAACTGCCTTGGAGAGGGGAGGCGTCTCTAGCCATTAAGGCGCT
>contig 126	3	GGGGAGGGTTGTCACGTCCTCAGAGAAGGGGCAAAGTAAAGCGTGCGGATTGGCATCAAACTGCCTTAGAGAGGGGAGGCGTCTCTAGCCATTAAGGCGC
>contig 127	4	GGGGAGCTTGTCGCTTGTCTTGGACATGACTATGAAGGGAGCTGAAGCATACGGGGGCACAGCTGGTTGGGAGCTATTGCCGCTTCCCTGATGAGCCCATG
>contig 128	3	AGACCAAATGAAAGAGCGGGCCATCTGGATTTTCGTCGTTGGGAAATAAAGGGAACGGCATTAGCGATAAGGCGTTCTCCCTTAGATGCTTCGATCTGGCCGAGTCAGGCAGGC
>contig 129	12	CTGTTTGTCGTGATCGTGTAGATCAAAAGAATGAACGGGCCATCTGTAACTTCGTCGTTGGGATACGGGAACGGCATTAGCGATAAGGCTGTTGGAAGAGGATTGCTATTAATATTTGTAGGGAG
>contig 130	12	TTAGCGATAAGGCGTTCTCCCTTAGATGCTTCGATCTGGCCGAGTCAGGCAGGCCGACCCGGTTAAGTTTGGGGAGCTTGTCGCTGGTCATGGACTTGACAATGAAGGGGACCGAAGTAGCCGAAAGGGACAACTGGCCGGG
>contig 131	3	CTTAGATGCTTCGATCTGGCCGAGTCAGGCAGGCCGACCCGGTTAAGTTGACTGTATTTATGTTTCCTATGTTTTCATAATTATTTACGTTATTTCTGCGGG
>contig 132	2	GGCCGAGTCAGGCAGGCCGACCCGATTAAGTTTGGGGTGCTTGTCGCTGGTCATGGACTTGACAATGAAGGGGACTGAAGTAGACCTAAGGGATCAACTA
>contig 133	2	AAGGGGACCGAAGTAGCCGAAAGGGACAACTGGTCGGTGGCATCGGAATCGAGGCGTCAGACGTCGAATCGACGCGGGAAACCAAGCAACAGAAAGGTCC
>contig 134	2	AGGCAGGGCGAAACCGGTTGGCTTCCGCATGTGGTAAGGCTGGTCTGGGGTGGTATGGGACGCCTCAGACGCCGCACGAGCGCTGTGCGCTGCGCCGGACC
>contig 135	42	ATGTGGTAAGGCTGGTCTGGGGTGGTATGGGACGCCTCAGACGCCGCACGAGCGCTGTGCGCTGCGCCGGACTCTCAGCCCGTGACAAGTGGTATCAGAGCGGGACGTAATTGCTGTTGTTGCGCGTTATGGTGGGGTCCTCGGGGGAGG
>contig 136	16	TGCTCTTCCGATCTGGGGTGGTATGGGACGCCTCAGACGCCGCACGAGCGCTGTGCGCTGCGCCGGACTCTCAGCCCGTGACAAGTGGTATCAGAGCGGGACGTAATTGCTGTTGTTGCGCGTTATGGTGGGGTCCTCGGGGGAGG
>contig 137	2	CCGTGACAAGTGGTATCAGAGCGGGACGTAATTGCTGTTGTTGCGCGTTATGGTGGGGTCCTCGGGGGAGGACTGTGCGGGTGCGTTGCCTAGGCGGGAG
>contig 138	4	CGCGTTATGGTGGGGTCCTCGGGGGAGGACTGTGCGGGTGCGTTGCCTAGGCGGGAGGGCTCGCACTGAAGACGGCCCCATCTTGCAATGGTCTGCAGGGGCAAGACCGGGAT
>contig 139	5	GTTTATGGTGGGGTCCTCGGGGGAGGACTGTGCGGGTGCGCGTTGCCTAGGGGGTGGCTCGCACTGAAGACGGCCCCATCCTGCAATGGTCTGCAGGGGCG
>contig 140	19	GGTCTGCAGGGGCAAGACCGGGATGGCCACCAGCAAGGACGGTTGCACGAGGTGAGGTGCGACTGGGCCAAAACACGCGGCGCGCATAGCAGTATTGTACGGGATCCTTGGGC
>contig 141	3	TGCAGGGGCAAGACCGGGATGGCCACCAGCAAGGCCGGTTGCACGAGGTGAGGTGCGACTGAGCAAAGTGACGCGGCGTGCTTTAGCAGTATTGTACGGGATCCTTGGGC
>contig 142	9	CCCATCTTGCAATGGTCTGCAGGGGCAAGACCGGGATGGCCACCAGCAAGGCCGGTTGCACGAGGTGTGGTGCGACTGGGCAAAAACACGCGGCGCGCATAGCAGTATTGTACGTGATCCTTGGGCC
>contig 143	9	CCCATCTTGCAATGGTCTGCAGGGGCAAGACCGGGATGGCCACCAGCAAGGCCGGTTGCACGAGGTGTGGTGCGACTGGGCAAAAACACGCGGCGCGCATAGCAGTATTGTACGTGATCCTTGGGCG
>contig 144	2	TTGCACGAGGTGTGGTGCGACTGGGCAAAAACACGCGGCGCGCATAGCAGTATTGTACGTGATCCTTGGGCAGAGTCCTTCTCCCCTACAGCACGAGGGCG
>contig 145	40	GCAGAGTCCTTCTCCCCTACAGCACGAGGGCGTGCTGCGTTTTGGGTGGGGGAGAATGTCACGACCCGATTGTCGTGACCGTGAAGATCAAAAGAAATAGCGGACCATCTGGATTTTCGTCGTTGGGAAATAAG

		GGAAC
>contig 146	32	AGAGTCCTTCTCCCCCTACAGCACGAGGGCGTGCTGCGTTTTCGGTGGGGGAGAATGTCACGACCCGATTGTCGTGACCGTAAAGACCAAATGAAAGAGCGGGCCATCTGGATTTTCGTCGTTGGGAAATAAAGG
>contig 147	8	ATTAAGGCGTTGTTCTCCCTTAGATGCTTCGATCTGGCCGAGTCAGGCAGGCCGACCCGATTAAGTTTGGGGTGCTTGTCGCTGGTCATGGACTTGACAATGAAGGGG
>contig 148	3	CTGACTGTATGCTAGGCAGGGCAACAGGACGACCCGAGTTCAGGGGCAACGTGTCACGACCCGATTGTCGTGACCGTAAAGACCAAATGAAAGAGCGGGC
>contig 149	6	TTAGCGATAAGGCGTTCTCCCTTAGATGCTTCGATCTGGCCGAGTCAGGCAGGCCGACCCGGTTAAGTTTGGGGAGCTTGTCGCTGGTCATGGACTTGACAATGAAGGGGG
>contig 150	2	GCGTTGTTCTCCCTTAGATGCTTCGATCTAGCCGAGTTAGGCAGGCCGACCCGGTTAAGTTTGGGGTGGTTGTCGCTGGTCATGGACTTGACAATGAAGGGGAC
>contig 151	27	GTGGTAAGGCTGGTCTGGGGTGGTATGGGACGCCTCAGACGCCGCACGAGCGCTGTGCGCTGCGCCGGACTCTCAGCCCGTGACAAGTGGTATCAGAGCGGGACGTAATTGCTGTTGTTGCGCGTTATGGTGGGGTCCCCGG
>contig 152	6	CCGATGACTGCCCCATCCTGCTAAGGTCTGGGGTAAGACCTGGATGGCCATCAGCGAGGCCCGGTTGCACGAGGTGAGGTGCGACTGAGCGAAGAAATACGGCGC
>contig 153	4	TCCTTCTCCCCCTACAGCACGAGGGCGTGCTGCGTTTTTGGTGGGGGAGAATGTCACGACCCGATTGTCGTGACCGTAAAGACCAAATGAAAGAGCGGGC
>contig 154	4	GTTGTTCTCCCTTAGATACTTCGATCTGGCCGAGTCAGGCAGGCCGACCCGGTTAAGTTTGGGGTGCTTGTCGCTGGTCATGGACTTGACAATGAAGGGGAC
>contig 155	2	AGTCAGGCAGGCCGACCCGGTTAAGTTTGGGGTGCTTGTCGCTGGTCATGGACTTGACAATGAAGGAGACCGAAGTAGGCCAAAGGGATCAACTGGTCGGGG
>contig 156	10	CTTCCGATCTGGTCTGGGGTGGTATGGGACGCCTCAGACGCCGCACGAGCGCTGTGCGCTGCGCCGGACTCTCAGCCCGTGACAAGTGGTATCAGAGCGGGACGTAATTGCTGTTGTTGCGCGTTATGGTGGGGTCCTCGGGGGAGG
>contig 157	2	CGAGATCATGGGCGAGTGGCAAGGCATGATCGAGATCATGGGCGAGTGACAAGTGGTATCAGAGCCGGACGTACTTGCTGTTGTTGCGCGTTTATGGTGGGGTCCTCGGGGGAG
>contig 158	2	TCTGCAGGGGCAAGACCGGGATGGCCACCAACAAGGCCGTTGCACGAGGTGAGGTGCGACTGAGCAAAGTGACGCGGCGTGCTTTAGCAGTATTGTACGGGATCCTTGGG
>contig 159	2	GAAACGTTTTTATAGAGCTTGTTGTCACGACCCGATTGTCGTGACCGTGAAGATCAAAAGAAATAGCGGACCATCTGGATTTTCGTCGTTGGGAAATAAGG
>contig 160	3	CCTCGGGAGGCCGCACGGGCGCTGTGTGCTGCGCCGGACTCTTAGCCCGTGACAGTGGTATCAGAGCGGTACGTACTTGCTGCTGTTGCGCGTTATGGTG
>contig 161	5	TCGGGACGCCGCACGGGCGCTGTGTGCTGCGCCGGACTCTAAGCCCGTGACAAGTGGTATCAGAGCGGGACGTACTTGCTGCTGTTGCGCGTTATGGTGGGG
>contig 162	3	AGTATTGTACGGGATCCTTGGGCAGAGTCCTTCTCCCCAACAGCACGAGGACGTGCTGGGTTTTGGGTGGGGGAGAATGTCACGACCCGTTTGTCGTGACCGTGTAGATCAAAAGAAAGAGCGGGC
>contig 163	2	AGTCCTTCTCCCCAACAGCACGAGGACGTGCTAGATTTTGGGTGGGGGAGAATGTCACGACCCGTTTGTCGTGACCGTGAGATCAAAAGAAAGAGCGGGCCAATTGGAACTTCGTCGATG
>contig 164	2	TCGTTGGGAAATAAGGGAACGGCATTAGCGATAAGGCGTTGTTCTTCCTTAGATGCTTTGGTCTTGCCGAGTTAGGCAGGCCGACCCGGTTAAGTTTGGG
>contig 165	20	ATATTTGTACATATTTAGTAGATTTCTAAACACATGTACAGGTTTGAGCCGAATGTCACGACCCGTTTGTCGTGACCGTGTAGATCAAAGGAAAGAGCGGGCC
>contig 166	5	ATATTTGTACATATTTAGTAGATTTCTAAACACATGTACAGGTTTGAGCCGAATGTCACGACCCGTTTGTCGTGACCGTGTAGATCAAAGGAAAGAGCGGAC
>contig 167	2	CAATGATTCTGTGTAAAATTACAGAAACATTACTTTAAAACTACGAGCCGTTTGTCACGACCCGTTTGTCGTGACCGTGAGATCAAAAGAAAGAGCGGGCC
>contig 168	2	CCAAGGCAGGGCGAAATCGGTTGGCTTCCGCATGTGGTAAGGCTTGTCTCGAGGTAGTGGGCGGCCTCGGGACGCCGCACGGGCACTGTGTGCTGCGCCGGAC
>contig 169	3	TAAGGCTTGTCTCGAGGTAGTGGGCGGCCTCGGGACGCCGCACGGGCGCTGTGTGCTGCGCCGGACTCTAAGCCCGTGACAAGTGGTATCAGAGCGGGAC
>contig 170	2	TCTCGAGGTAGTGGGCGGCCTCGGGATGCCGCACGGGCGCTGTGTGCTGCGCCGGACTCTAAGCCCGTGACACTAAGCTTGAGTGATCTATGAAAAGTAC
>contig 171	3	TGCGCGTTATGGTGGGGTCCTCGAGGGAGGACTGTGCGGGTGCGTTCCCTAGGGGGGCTCGCGCCGACGACTGCCCCATCCTGCAATGGTCTGCAGGGGC
>contig 172	3	CTAGGCGGGGGGCTCGTGCCGTTGACTGCCCCATCCTGCTAAGGTCTGGGGTAAGACCTGGATGGCCATCAGCGAGGCCCGGTTGCACGAGGTGAGGTGCGACTGAGCGAAGAAATACGGCGC
>contig 173	5	GAGTCCTTCTCCCAAACAGCACGAGGACGTGCTGGGTTTTGGGTGGGGGAGAATGTCACGACCCGTTTGTCGTGACCGTGTAGATCAAAGGAAAGAGCGGGC
>contig 174	2	CCCAACAGCACGAGGACGTGCTGGGTTTTGGGTGGGGGAGAATGTCACGACAAACGGGTCGGGGGCTAGTGCCGCTCCCCTGATAAGTCCAAGGCAGGGC
>contig 175	2	GGGTTTTGGGTGGGGGAGAATGTCACGACCCGTTTGTCGTGACCGTGTAGATCAAAGGAAAGAGCGGGCCATCTGGAACTTCGTCGTTGGGTTATTGGGGAG
>contig 176	4	GGTTTTGGGTGGGGGAGAATGTCACGACCCGTTTGTCGTGACCGTGTAGTCCAAAGGAAAGAGCGGTCCATCTGGAACTTCGTCGTTGGGTTATGGGGACGACATTAGCATTAAGG
>contig 177	8	CATGTACAGGTTTGAGCCGAATGTCACGACCCGTTTGTCGTGACCGTGTAGATCAAAGGAAAGAGCGGGCCATCTGGAACTTCGTCGTTGGGTTATTAGGGAG
>contig 178	2	GATCAACTGGTCGGGGCTAGTGCCGCTCCCCTGATGAGCCCAAGGCAGGGCGAAACCGGTTGGCTTC

		CGCGTGTGGTAAGGCTTGTCTTGAGGTAGTGGGC
>contig 179	5	CTCAGACGCCGCACGAGCGCTGTGCGCTGCGCCGGACTCTCAGCCCGTGACAAGTGGTATCAGAGCG GGACGTAATTGCTGTTGTTGCGCGTTATGGTGGGGTCCTCGGGGGTGG
>contig 180	2	GGGGTCCTCGGGGGAGGACTGTGCGGGTGCGCGTTGCCTAGGGGGTGGCTCGCACTGAAGACGGCCC CATCCTGCAATGGTCTGCAGGGGCAAGACCGGGATGGC
>contig 181	18	GGCGTTGTTCTTCCTTAGATGCTTTGGTCTTGCCGAGTTAGGCAGGCCGACCCGGTTAAGTTTGGGG AGCTTGTCGCTTGTCTTGGACATGACTATGAAGGGAGCCGAAGCATACGAGGGGCACAGCTGGTCGG GG
>contig 182	34	ATTAAGGCGCTCTGCCCTAGTTGCGTATTGGCTTGGCCGAGTTAGGCATGCCGACCCGATTAAGTTT GTGGAGCATGTCGCTTGTCTTGGACATGACTATGAAGGGAGCCGAAGCATACGAGGGGCTCAGCTGG T
>contig 183	2	GCTCTGCCCTAGTTGCGTATTGGCTTGGCCGAGTTAGGCATGCCGACCCGATTAAGTTTGGGGAGCA TGTCGCTTGTCTTGGACATGACTATGAGGGGGAG
>contig 184	2	GACATGACTATGAAGGGAGCCGAAGCATACGGGGGGCACAGCTGGTCTTGCCTAGAATCGTACTAAG GGAATGCTGGATAGCAACACAGAAATTCAAGAG
>contig 185	3	AAAGCGTGCGGATTGGCATCAAACTGCCCTGGAGAGGGGGAGGCGTCTTTAGCCATTAAGGCGCTCTG CCCTAGTTGCGTATTGGCTTGGCCGAGTTAGGCATGCCGACCCGATTAAGTTTGTGGAGC
>contig 186	16	AGGCGCTCTGCCCTAGTTGCGTATTGGCTTGGCCGAGTTAGGCATGCCGACCCGATTAAGTTTGGGG AGCATGTCGCTTGTCTTGGACATGACTATGAAGGGAGCCGAAGCATACGAGGGGCACAGCTGGTCGG G
>contig 187	3	ATTAAGGCGCTCTGCCCTAGTTGCGTATTGGCTTGGCCGAGTTAGGCATGCCGACCCGATTAAGTTT GTGGAGCATGTCGCTTGTCTTGGACATGACTATGGAGGG
>contig 188	2	AGGGAGCCGAAGCATACGAGGGGCACAGCTGGTCGGGAGCTATTGCCGCTTCCCTGATGAGCCCATG GCAGGGCGAAACCAGTCTGCGCGCACAGGCAGGTCGGG
>contig 189	4	GACACTATATAGAAGTGGTATTGGATTTGATTCGACATATGCCGCTTCCCTGATGAGCCCATGGCAG GGCGAAACCAGTCTGCGCGCACAGGCAGGACGGGCGG
>contig 190	3	CACTATATAGAAGTGGTATTGGATTTGATTCGACATATGCCGCTTCCCTGATGAGCCCATGGCAGGG CGAAACCAGTCTGCGCGCACAGGCAGGACGGGCGTGTCCGGG
>contig 191	2	CAGGACGGGCGTGTCCGGGGCGGTATGGGACGCCTCGGGCGCGGAGCGCCGCGGTCCGTGTCATTTG GTATCAGAGCAAACATGCTCTTCAACTTCTGTGGCGAG
>contig 192	4	GGACGGGCGTGTCCGGGGCGGTATGGGACGCCTCGGGCGCTGAGCGCCGCGGTCCGTGTCATTTGGT ATCAGAGCAAACATGCTCTTCAACTTCTGTGGCGAGGTGTCAAGCTCATGAATGCACC
>contig 193	3	GATCTCGGGCGTGTCCGGGGCGGTATGGGACGCCTCGGGCGCTGAGCGCCGCGGTCCGTGTCATTTG GTATCAGAGCAAACATGCTCTTCAACTTCTGTGGCGAGGTGTCAAGCTCATGAATGCACC
>contig 194	11	GCTGAGCGCCGCGGTCCGTGTCATTTGGTATCAGAGCAAACATGCTCTTCAACTTCTGTGGCGAGGT GTCAAGCTCATGAATGCACAAAGCTAGCGGAAGGCACAAGTGCAAAGATGGGGGC
>contig 195	2	CAGGCGCCGCGTAGGCGCGCTGCGCCACGGTCGGTTCCAGGTCCGTTTCATATGGTATCAGAGCAAA CATGCTCTTCTACTTCTGTTGCAAGGTGTGGGC
>contig 196	33	TTGCACTTCAACTTCTGTGGCGAGGTGTCAAGCTCATGAATGCACAAAGCTAGCGGAAGGCACAAGT GCAAAGATGGGGGCGCTGAGTTACAGAGTGGGGCATGACGGTCCTTGGGGCAC
>contig 197	32	CGTCTGTGGCGAGGTGTCAAGCTCATGAATGCACAAAGCTAGCGGAAGGCACAAGTGCAAAGATGGG GGCGCTGAGTTACAGAGTGGGGCATGACGGTCCTTGGGGCAC
>contig 198	5	TGGCGAGGTGTCAAGCTCATGAATGCACAAAGCTAGCGGAAGGCACAAGTGCAAAGATGGGGGCGCT GAGTTACAGAGTGGAGCATGACGGTCCTTGGGGC
>contig 199	2	GGCGAGGTGTCAAGCTCATGAATGCACAAAGCTAGCGGAAGGCACAAGTGCAAAGATGGGGGCGCTG AGTTACAGAGTGGAGCATGACGGTCCTCGGGGC
>contig 200	14	GGCTAGGTGTCAAGCTCATGAATGCACAAAGCTAGCGGAAGGCACAAGTGCAAAGATGGGGGCGCTG AGTTACAGAGTGGGGCATGACGGTCCTTGGGGCAAACATACTACTTCTCCGCCAAAGTAAAGTGGGC TTGAGC
>contig 201	14	ATCTCGAGGTGTCAAGCTCATGAATGCACAAAGCTAGCGGAAGGCACAAGTGCAAAGATGGGGGCGC TGAGTTACAGAGTGGGGCATGACGGTCCTTGGGGCAAACATACTACTTCTCCGCCAAAGTAAAGTGG GCTTGAGC
>contig 202	3	GCGGAAGGCACAAGTGCAAAGATGGGGGCGCTGAGTTACAGAGTGGAGCATGACGGTCCTTGGGGCA AACATACTACTTCTCCGCCAAAGTAAAGTGGGC
>contig 203	2	GCTTGAGCAAATGAAACTGTTGCGAACTCTAGAAGAAACCACGAAGTGGACGCGCCGACGAAGTGTG AAGCTCTCCATGGTTTCGTGCCTAAGTCCGAGG
>contig 204	2	ACAGCTGGTCCTCTCCATGGTTTCGTGCCTAAGTCCGAGGTAGGCAGCACGAGGAGGTATTGCTAGC ATGATGGGTGCAATGTTCCGAGTGGAAGACGAG
>contig 205	5	CCAGGGTTGGCAGCACGAGGAAGTATGGCTGTTAGCATGATACTGGGTGCAAGTTCCGTGCGGAGAC GAAAGGAGTTGCAGCGAAAGTAATGCGTCGGGTCGCCGTATCATCTAGTGGGGGAG
>contig 206	27	TCTCGAGGAGGTATTGCTAGCATGATGGGTGCAATGTTCCGAGTGGAAGACGAAAGGAGTAGCAGCG AAAGTAATGCGTCGGGTCGCCGCATCAGCCAGGTGGGGGAGGGTTGTCACGTCCTCAGAGAAGGGGC
>contig 207	7	CAATGTTCCGAGTGGAAGACGAAAGGAGTAGCAGCGAAAGTAATGCGTCGGGTCGCCGCATCAGCCA GGTGGGGTAGGGTTGTCACGTCCTCAGAGAAGGGGC
>contig 208	10	AATGTTCCGAGTGGAAGACGAAAGGAGTAGCAGCGAAAGTAATGCGTCGGGTCGCCGCATCAGCCAG GTGGGGGAGTGTTGTCACGTCCTCAGAGAAGGGGCAAAGTAAAGCGTGC
>contig 209	4	CGTCGGGTCGCCGCATCAGCCAGGTGGGGGAGGGTTGTCACGTCCTCAGAGAAGGGGCAAAGTAAAG CGTGCGGATTGGCATCAAACTGCCTTGGAGAGGGGC
>contig 210	4	GTCGGGTCGCCGCATCAGCCAGGTGGGGGAGTGTTGTCACGTCCTCAGAGAAGGGGCAAAGTAAAGC

		GTGCGGATTGGCATCAAACTGCCTTGGAGAGGGGAGGC
>contig 211	6	ATCTATTGGCTTGGCCGAGTTAGGCATGCCGACCCGATTAAGTTTGGGGAGCATGTCGCTTGTCTTG GACATGACTATGAAGGGAGCCGAAGCATACGAGGGGC
>contig 212	4	GGAGCCGAAGCATACGAGGGGCACAGCTGGTCGGGAGCTATTGCCGCTTCCCTGATGAGCCCATGGC GGGGCGAAACCAGTCTGCGCGCACAGGCAGGACGGGCG

Table S3. Retrozyme-specific small RNAs in *N. benthamiana* plants. Small RNAs sequence reads, which exactly match the retrozyme sequences, were identified in publicly available libraries of HTS reads obtained for small RNA fractions isolated from different organs of *N. benthamiana* plants.

Dataset	Tissue	Run	Number of matches	Total for dataset
**Experiment 1**				
SRX1081187	Root	SRR2087151	92	214
		SRR2087152	122	
SRX1081185	Leaf	SRR2087149	132	215
		SRR2087150	83	
SRX1081184	Seedling	SRR2087147	74	306
		SRR2087148	232	
SRX1081183	Flower	SRR2087143	369	450
		SRR2087144	81	
SRX1081188	Stem	SRR2087153	47	267
		SRR2087154	220	
**Experiment 2**				
SRX2502227	Leaf	SRR5186275	114	114
SRX2502225	Root	SRR5186273	59	59
SRX2502223	Stem	SRR5186271	105	105

Table S4. Primers used in this study.

NbRZ1 cloning		
NbRZ-P-Xho	CGCTCGAGATGTTAACCTTTGTTATAAAAGGAGC	
NbRZ-3-M	GCTCTAGAGCAGTGGGAGGAGCCAGGTGT	
LTR-GFP cloning		
RZ-LTR-P	CGCGAATTCTGTCACGACCCGATTGTCGTG	
RZ-LTR-M	CCGCCATGGTGTCACGGGACGAGAGTCCG	
35S-NbRZ1-Tag cloning		
NbRz-FLAG1-P	GATTACAAGGATGACGATGACAAGGGGTGGCTCGCACTGAAGA	
NbRz-FLAG1-M	CTTGTCATCGTCATCCTTGTAATCCCTAGGCAACGCGCACCCG	
Bisulfite sequencing		
RZ-BisS-P1	ATAAGATGTTAAYYTTTGTTATAAAAGGAG	
RZ-BisS-M1	CCCACCATAAACRCRCAACAACA	
prPDSb-P320	GTGGGAGATGTGTGYAAAGTGGT	
prPDSb-M	CTRTTACCCACAAAAARATACCCCT	
Detection of Group 1 retrozyme RNA in *N. benthamiana* plants		
RZ-PP4-F	TGCCCAAGGATCCCGTACAA	Expected product size: 117 bp
RZ-PP4-R	AATGGTCTGCAGGGGCAAG	
Detection of Group 2 retrozyme RNA in *N. benthamiana* plants		
RZ8-PP5-F	TCAACTTCTGTGGCGAGGTG	Expected product size: 217 bp
RZ8-PP5-R	AGAGCTTCACACTTCGTCGG	
RZ9-PP1-F	GAAGTGTGATGCCGTCTCCA	Expected product size: 111 bp
RZ9-PP1-R	CCTTTCGTCTTCCACACGGA	
Detection of NbRZ1-Tag		
NbRZ-int-M	TCTGCCCAAGGATCCCGTACAA	Expected product size: 176 bp
FLAG-det-P	GATTACAAGGATGACGATGACAAG	
Detection of circular (+) strand of NbRZ1-Tag in upper leaves		
RZ(-)circ-det-D1	GAGGGCGTGCTGCGTTTTC	Expected product size: 525 bp
FLAG-det-M2	AGCCACCCCTTGTCATCGTC	
Detection of NbRZ circular (+) strand		
RZcirc-det-C1	AGTCCTTCTCCCCCTACAGC	Expected product size: 665 bp
RZ-PP4-R	AATGGTCTGCAGGGGCAAG	
Detection of NbRZ circular (-) strand		
RZcirc-det-A1	CTTGCCCCTGCAGACCATT	Expected product size: 588 bp
RZcirc-det-A2	TTGTACGGGATCCTTGGGCA	
Detection of GFP mRNA		
GFP-C3-PP1-F	TCAGTGGAGAGGGTGAAGGT	Expected product size: 240 bp
GFP-C3-PP1-R	GCGTCTTGTAGTTCCCGTCA	
Detection of F-box protein gene and mRNA		
F-Box-F	GGCACTCACAAACGTCTATTTC	Expected product size: 127 bp
F-Box-R	ACCTGGGAGGCATCCTGCTTAT	
Retrozyme 5'-RACE		
RZ-RACE-OUT	ACGCGCAACAACAGCAAGTAC	
RZ-RACE-IN	TCCGGCTCTGATACCACTTGT	
LTR-GFP 5'-RACE		
GFP-C3-DetR (outer)	ATGTGTAATCCCAGCAGCAGT	
GFP-C3-ovlX-M (inner)	ATAGTTGTACTCCAGTTTGTGTCCG	
